# Early hospital discharge following delivery during wartime is not associated with increased rates of postpartum hospital referrals

**DOI:** 10.1371/journal.pone.0335688

**Published:** 2026-10-16

**Authors:** Shlomit Blumenfeld, Lital Shaham, Noa Brenner, Gad Segal, Michal J. Simchen, Keren Zloto, Mordechai Dulitzky, Eyal Sivan, Nirit Agay, Alina Weissmann-Brenner

**Affiliations:** 1 Department of Obstetrics and Gynecology, Sheba Medical Center, Ramat Gan, Israel; 2 Faculty of Health Sciences and Medicine, Tel-Aviv University, Tel Aviv, Israel; 3 Education Authority, Chaim Sheba Medical Center, Ramat Gan, Israel; 4 Biostatistics & Biomathematics Unit, Data & Analytics Division, Sheba Medical Center, Ramat Gan, Israel; Australian National University Medical School, AUSTRALIA

## Abstract

**Background:**

During war, significant adaptations take place in hospitals to improve the treatment of soldiers and civilians. For example, low-risk women were discharged earlier from hospitals following delivery. The aim of the study was to compare the rate of postpartum-visits to the gynecological-emergency-room (GER) between wartime to a similar period in the previous year.

**Methods:**

A retrospective study compared the visits of postpartum-women in the GER to a similar period in the previous year.

**Results:**

The study included 801 women who delivered in the first month of the war in 2023 and 913 who delivered in the parallel period in the previous year. There were no significant changes in maternal age, gravidity, parity, mode of delivery, birthweight, meconium, low Apgar scores, and the rate of admissions to the neonatal intensive care unit. There were significantly less elective cesarean sections during wartime (57% vs. 92%; p < 0.0001). The duration of postpartum hospitalization was significantly shorter during wartime [median length of 53.2 [Interquartile-range (IQR] 42.4–72.4) hours vs. 64.1 (IQR 47.4–82.8) in the previous year (p < 0.0001)]. There were no significant differences in the rate of total referrals of postpartum-women to the GER (8.7% vs. 8.8%; p > 0.05), and no differences in referrals due to infections or bleeding. There was no difference in the rate of admission of postpartum-women (17% vs. 14%; p = 0.73).

**Conclusions:**

Shortening of hospitalization following delivery during war is feasible, does not cause increase postpartum visits to the GER, and does not increase GER visits due to infections or bleeding.

## Introduction

Armed conflicts place exceptional strain on healthcare systems, necessitating rapid adaptation by both institutions and practitioners to maintain effective care delivery amid pervasive uncertainty and resource constraints. Common responses to these pressures include reallocating medical staff, modifying patterns of patient referral and hospitalization to accommodate surges of injured military personnel and civilians, and reorganizing hospital operations to balance urgent demands with routine healthcare services [1–11]. One frequent strategy employed during wartime is the reduction of hospital length of stay for select groups, such as low-risk postpartum women. This approach is intended to maximize bed availability and minimize patients’ exposure to conflict-related risks. However, the potential impacts of shortened hospitalization on postpartum patient outcomes within this context remain insufficiently understood. The importance of examining healthcare responses in wartime contexts has gained prominence, especially given the increasing frequency and complexity of conflicts worldwide.

On October 7, 2023, Hamas organization launched a war against Israel including massive missile attacks and territory invasion, resulting in over 1,400 lives lost and the abduction of 251 hostages. On October 8^th^ 2023 Hezbollah subsequently joined, launching rockets at Israel, including attacks targeting civilian populations, leading to declaration of war by Israel [12–18]. In addition to the immediate casualties and individuals injured in the battlefield, many civilians’ lives were severely impacted. About 143000 civilians were evacuated from their homes in the north and south of Israel along the borders with Lebanon and Gaza [19]. Consequently, the healthcare system has been compelled to address significant demographic changes resulting from these circumstances. Israel is a high-income setting with mature emergency and trauma care infrastructure, shaped both by repeated exposure to armed conflict and by substantial domestic investment in trauma medicine. In this context, hospitals transitioned to emergency operation modes, including the shortening of postpartum hospitalizations for low-risk women—both following vaginal and cesarean deliveries. Such measures raise important questions about their impact on postpartum follow-up, particularly visits to gynecological emergency rooms (GER), where early detection and management of postpartum complications are critical. Currently, there is a paucity of data evaluating whether these rapid adjustments—especially shortened postpartum hospitalization—affect postpartum health-seeking behavior and clinical outcomes. The present study aims to fill this knowledge gap by investigating whether the expedited discharge policies implemented during the war influenced postpartum GER visits compared to a similar period in the previous year.

## Methods

The study was performed in a tertiary hospital in the center of Israel. During the study period of the first month of the war, from October 7^th^ 2023 and November 5^th^ 2023 there were 44 clusters of hundreds of missile attacks on the Tel Aviv area [13]. During that period the Israeli Ministry of Health revised its guidelines and permitted hospitals to shorten the length of postpartum hospitalization.

Following delivery, low-risk patients were offered to be discharged earlier from the hospital after careful examination of them and their newborns, after 24–36 hours for vaginal deliveries and after 48 hours for cesarean sections.

Low risk patients were defined as generally healthy women, with no delivery complications such as profound vaginal bleeding, hypertension or infection.

Each postpartum woman received detailed explanations from both an obstetrician and a maternity nurse, as well as a discharge letter specifying when to seek medical attention (for example, in cases of fever, bleeding, abnormal discharge, unusual appearance of the surgical scar, etc).

Study cohort and patients’ characteristics.

This study’s cohort of patients’ data was accessed from their electronic medical records (EMRs) which are routinely used for clinical purposes. All deliveries during the study period were included with no exclusions.

The study was conducted in accordance with the Strengthening the Reporting of Observational Studies in Epidemiology (STROBE) guidelines.

### Ethics approval and consent to participate

Approval for the study was granted by the Institutional Review Board (IRB) of the Chaim Sheba Medical Center, no. SMC 23-0820. Data was retrieved on 22/02/24 only after an approval was given by our institutional review board.

The authors had no access to information that could identify individual participants during or after data collection

Patients’ data from the first month of war (7.10.23–5.11.23) was compared with the corresponding month in the previous year (17.10.22–15.11.22). The shift in dates stem from the need to relate to the Jewish holidays affecting patients’ routines and the healthcare system and avoid the potential resulting bias.

For each of these two periods, a dataset containing all deliveries was created, including delivery characteristics, postpartum referrals to the GER and re-admissions during the postpartum period.

Postpartum period was defined as the first 42 days following delivery.

### Outcome measures

The study evaluates several clinical outcomes: rate of postpartum referrals to the GER, measured as a binary variable, rate of postpartum readmissions, measured as a binary variable, time (days) from delivery to GER referral, reason for GER referral, and duration of postpartum hospitalization.

Potential confounders were maternal age at delivery, a continuous variable, number of previous births (parity), categorized as 1, 2, 3, 4, or 5 + , gestational week, categorized as a binary variable (below or above 37 weeks), and fetus count, categorized as singleton or multiple births (one or more).

Delivery characteristics (meconium, mode of delivery, Apgar score, birthweight, fetus gender, need for intensive care unit hospitalization for the newborn, cases of non-reassuring monitor, length of hospital stay after deliveries) are possible mediators of the association between period and postpartum readmissions.

### Statistical analysis

Mean and standard deviation (relating to normally distributed continuous variables), median and IQR (relating to non-normally distributed continuous variables) of the aforementioned patients’ characteristics (delivery characteristics and length-of-stay in hospital following delivery) were computed for each period. Normality was assessed by Kolmogorov-Smirnov tests.

Categorical variables were compared by Chi-square tests or by Fisher exact tests where a Chi-square test was not valid, and continuous variables were compared via Wilcoxon non-parametric tests where distribution was non-normal, or by or via t-tests where distribution was normal.

In order to compare post-partum visits to the GER in the two periods while controlling for potential confounders, a multivariable logistic regression model was fitted to the data, with period as the exposure of interest, and age at delivery, gestational week (below or above 37), fetus count (one or more), and number of previous births (1,2,3,4, 5+) as confounders. Delivery characteristics and hospital length-of-stay are mediators so they were not included in the model.

In each cohort, a Kaplan-Meier curve for the time until a post-partum referral to the GER was fitted, and the two curves were compared via a log-rank test. A Multivariable Cox Proportional Hazards model was fitted to the data, with time to post-partum referral as the dependent variable, period as the exposure of interest, and age at delivery, gestational week (below or above 37), fetus count (one or more), and number of previous births (1,2,3,4, 5+) as potential confounders. This modes’ results served as a sensitivity analysis for the Multivariable Logistic regression model.

Statistical significance was determined whenever the p value was < 0.05. All analytics were done using R software (version 3.6.2; R Core Team, 2019).

A post hoc power analysis demonstrated that with a total sample of 1,714 deliveries, the study had ~ 80% power to detect a 4.4% absolute increase in the rate of GER referrals (alpha = 0.05).

## Results

### Birth and newborn characteristics

During the first month of the war, 801 women gave birth in our medical center, compared to 913 in the parallel period in the previous year. Characteristics of the study population are presented in Table 1. There were no significant differences in maternal age, gravidity, parity, the rate of meconium, mode of deliveries, rate of low Apgar scores, birthweight and the rate of admissions to the Newborn Intensive Care Unit (NICU). The percentage of elective cesarean sections of the total cesarean sections was significantly lower during wartime (57% vs. 92%; p < 0.0001).

**Table 1:** Characteristics of study population.

	Wartime period		Control		p-value
	07/10/2023-05/11/2023		17/10/22-15/11/22		
	N = 801	%	N = 913	%	
**Maternal age (median; [IQR])**	32.3 [28.6, 35.8]		32.6 [29.2, 36.5]		0.23
**Nuligravida (N)**	290	36%	301	33%	0.36
**Parity (median; [IQR])**	2		2		0.93
**No. of fetuses (N)**					
1	779	97.3%	867	95.0%	0.02
2	21	2.6%	45	4.9%	
3	1	0.1%	0	0.0%	
4	0	0.0%	1	0.1%	
**Gestational age at delivery (median; [IQR])**	39.2 [38.2, 40.2]		39.1 [38.0, 40.1]		0.01
**Meconium**	10	1.2%	19	2.1%	0.25
**Mode of delivery**	475	59.3%	576	63.1%	0.16
normal vaginal delivery	475	59.3%	576	63.1%	0.16
vacuum extraction	52	6.5%	42	4.6%	
forceps delivery	4	0.5%	2	0.2%	
cesarean section	211	26.3%	284	31.1%	
**Precipitated labor**	10	1.2%	9	1.0%	0.16
**Cesarean delivery**					
1- elective (% of total cesarean section)	126	57%	277	92%	<0.0001
2- non elective (% of total cesarean section)	76	34%	8	3%	
3- urgent (% of total cesarean section)	21	9%	15	5%	
total	223		300		
**Birthweight**	3159 ± 606.1		3130 ± 590		0.33
**NRHFR**	184	23.0%	200	21.9%	0.64
**NICU**	8	1.0%	7	0.8%	0.8
**Duration of hospitalization (hours; median; [IQR])**	53.2 [42.4, 72.4]		61.4 [47.4, 82.8]		<0.0001
**Duration of hospitalization after Cesarean after cesarean (median; [IQR])**	68.6 [49.3, 93.7]		89.1 (64.6, 110.3]		<0.0001
**Duration of hospitalization after vaginal delivery (median; [IQR])**	49.3 [40.6, 63.3]		54.4 [44.8, 69.5]		<0.0001

NRFHR – non reassuring fetal heart rate.

NICU – neonatal intensive care unit.

Non elective cesarean section – cesarean section not planned in advance, performed due to complications that arise during pregnancy or labor, such as breech presentation that presented in labor, failure to progress in labor, etc.

Urgent Cesarean section – cesarean section performed due to immediate maternal and/or fetal risk, such as NRFHR, eclampsia etc.

Meconium – presence of meconium (early stool) in amniotic fluid.

*P-value comparing 1 versus 2 + fetuses.

Length of postpartum hospitalization and clinical outcomes.

The length of postpartum hospitalizations was significantly shorter by 13.4% during wartime in all patients, by 9.4% for patients following vaginal delivery and by 23% for patients following cesarean deliveries (p < 0.0001).

There were overall 70 postpartum women who visited the GER during the war (8.7%) and 80 in the control group (8.8%) with no statistical significance.

Postpartum women who were discharged earlier tended to present 1.6 days earlier to the GER, with no statistical difference. There was no significant difference between the two periods in the time until post-partum referral to the GER (p = 0.93), (Fig 1).

**Fig 1:**
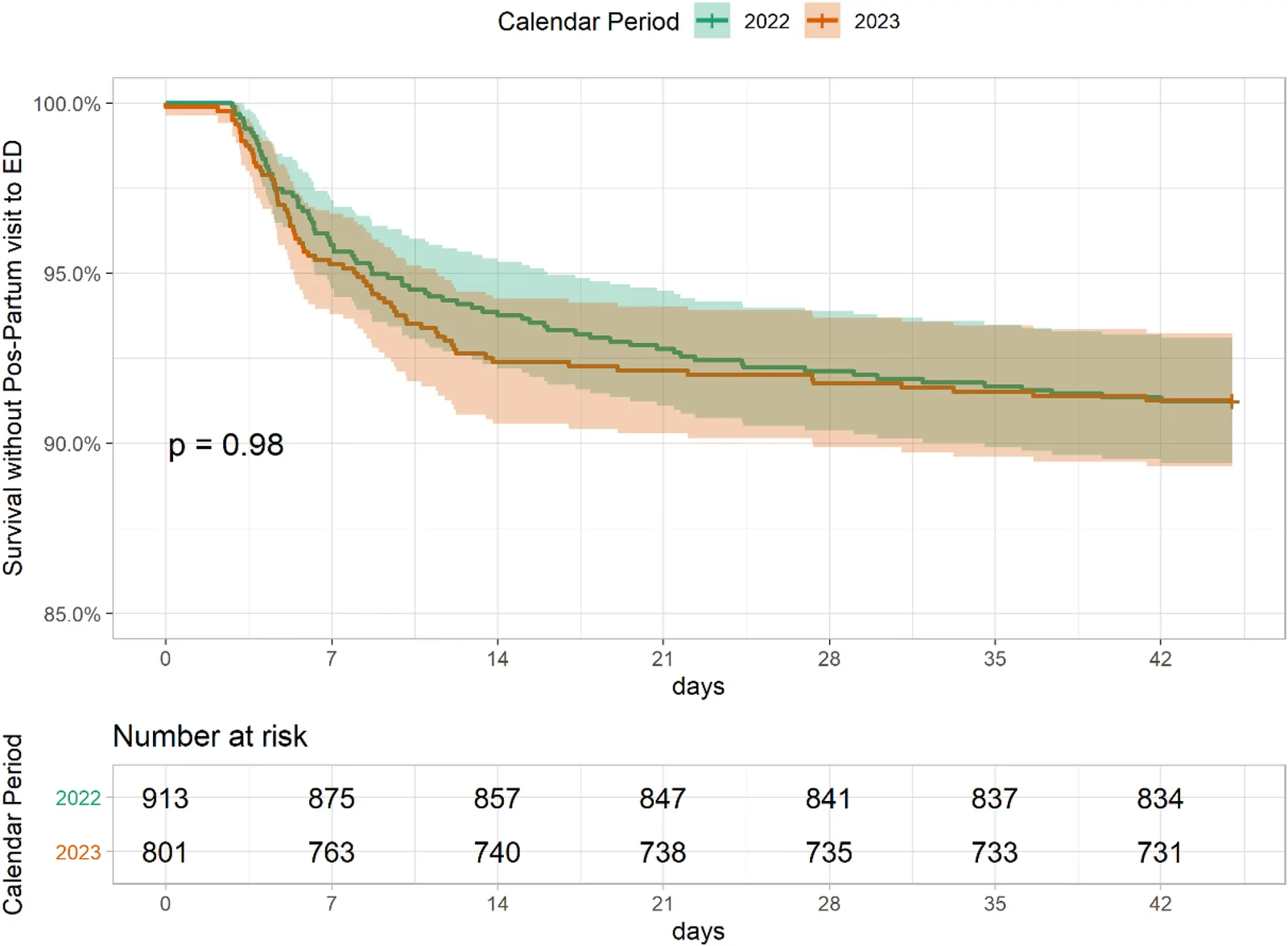
Time from postpartum discharge to postpartum referral to gynecological emergency room in the 42 days following delivery.

There was no statistically significant difference in the rate of postpartum visits due to infection (15.7% during the was compared to 16.3% in the control group) or bleeding (0 during the war compared to 5% in the control group). There was also no difference in the rehospitalization rate (17% during the was compared to 14% in the control group).

Similarly, multivariable logistic regression found no evidence of a difference between the wartime and control periods, with confidence intervals compatible with moderate increases or decreases in postpartum GER risk (OR = 1.01, 95% CI 0.72–1.41, p = 0.98). In the sensitivity analysis, the Multivariable Cox model showed a similar result (HR = 1.01, 95% CI 0.73, 1.40).

## Discussion

The present study indicated that reduced length of postpartum of hospitalization following uncomplicated vaginal or cesarean delivery during wartime, which did not lead to increased postpartum referrals to the GER or hospital readmissions. Women who were discharged earlier tended to visit the GER sooner, with an average of 1.6 days earlier presentation. This shift in timing may reflect increased vigilance among recently discharged mothers, possibly due to the heightened sense of uncertainty associated with the wartime conditions, and maybe reduced access to routine postpartum support.

Quantitatively, the median postpartum duration of hospitalization decreased by 13.4%, with an average shortening of 5.1 hours following vaginal delivery and 20.5 hours following cesarean delivery compared to a similar period in the previous year. These findings suggest that, even under extreme circumstances such as war, safe early discharge protocols can be implemented without compromising maternal health outcomes.

Prior to the Covid-19 pandemic there was a trend of shortening of postpartum hospitalizations in order to promote a more family-centered approach to birth, improved rest for the mothers, and less exposure to nosocomial infections [20–23]. A systematic review from 2017 on the impact of early postpartum discharge after vaginal delivery reported no statistical difference in maternal and neonatal morbidity and maternal and neonatal readmission rates. The authors concluded that there was lack of clinical evidence and economic evaluation to support or evidence against the widespread use of early discharge [24]. The Covid-19 pandemic introduced a need for significant changes in patient care. Hospitals implemented early discharge of low-risk postpartum patients following uncomplicated vaginal and cesarean deliveries, in order to optimize their care, accommodate the high influx of non-obstetrical COVID-19 admissions that required increased hospital resources, as well as to decrease the potential exposure of both patients and medical staff in the hospital [25–30].

Correspondingly, a study conducted in France reported that early postpartum discharge within 48–72 hours following vaginal delivery or 72–96 hours after cesarean did not increase emergency room visits or readmission rates during the first 28 postpartum days during the pandemic [31].

In the context of wartime, hospitals are compelled to make even more extensive adjustments. Among these are transfer of entire departments to sheltered places, shifting patients between departments in order to make room for injured soldiers and injured civilians, and shortening of hospitalization in selected cases. Given that most postpartum women are typically healthy and low risk, earlier discharge during conflict offers several advantages: it reduces overcrowding in postpartum wards, thereby lowering potential casualties if the hospital comes under attack; and it allows for the redistribution of medical resources—such as beds and staff—to other critical care areas facing increased demand. Our findings demonstrate that shortening postpartum hospitalization is feasible and does not increase the burden on gynecological emergency services, including visits related to infections or bleeding. An interesting finding in the present study is the significant reduction in the proportion of elective cesarean sections during wartime (57% vs. 92% of total cesareans, p < 0.0001). This pronounced decline likely reflect significant shifts in obstetric decision-making, potentially driven by maternal concerns over prolonged hospitalization and disruptions in postnatal care amid the war. Resource limitations or logistical constraints may also have contributed However, as our hospital is a tertiary medical center, stuffing changes did not result in cancellations of elective cesarean sections. Another possibility may be that the population delivering during wartime differed from the control population due to regional evacuations or altered referral patterns. A nationwide investigation would be needed to explore this further. Alternatively, the wartime setting may have led to an increase in obstetrical complications, thereby elevating the proportion of urgent and non-elective cesarean sections. Importantly, despite the reduction in elective cesarean sections, there were no significant differences in neonatal outcomes, including rates of meconium-stained amniotic fluid, low Apgar scores or rate of admissions to the Newborn Intensive Care Unit. These findings suggest that the shift in cesarean section practice during wartime did not adversely impact neonatal health.

The study possesses several strengths. To our knowledge, this is the first study to examine the feasibility of early postpartum discharge of uncomplicated deliveries in the setting of wartime, providing valuable insights from a large, tertiary medical center with diverse maternal demographics, enhancing the generalizability of our findings. The medical system in Israel is developed and advanced, facilitating comparison to the western world. In low socioeconomic countries it is even more critical to carefully evaluate whom to discharge earlier and under what conditions.

A post hoc power analysis demonstrated that with a total sample of 1,714 deliveries, the study had ~ 80% power to detect a 4.4% absolute increase in the rate of GER referrals (alpha = 0.05). The fact that no such increase was observed despite this power reinforces the conclusion that early discharge during wartime is a safe and feasible strategy.

This study shares the limitations inherent to retrospective investigations and the findings are interpreted without randomization and strict control of all study aspects. Future research should include a nationwide study on data in the entire country is recommended as well as a comparison between low and high-risk pregnancies, and between women with different comorbidities.

Another limitation is the irreproducibility of the study in other countries, because of the unique condition of Israel: Relatively small population, where military service and reserve military service are mandatory, leading to a high rate of recruitment among the general population as well as among medical staff. Another limitation is the lack of data regarding whether women who were discharged sought care at other medical centers following delivery. It is important to note that not all clinics were operational during the war. Given that our hospital is a tertiary care facility situated in central Israel—rather than in the northern or southern regions, which experienced significantly more frequent missile attacks—we assume postpartum women requiring medical attention, would have presented to our hospital.

Focusing on the early phase of the war—an unforeseen attack involving extensive missile strikes—allowed us to evaluate hospital preparedness and responsiveness under extreme conditions. Our findings highlight the importance of swift and effective emergency protocols maintaining healthcare services in unpredictable wartime situations.

## Conclusions

In conclusion, our data suggest that shortening of postpartum hospitalization for low-risk women during wartime does not increase the rate of postpartum referrals to the GER. This approach can inform policy decisions at both national and international levels, aiding efficient resources allocation during crises. Clear communication and patient education at discharge are essential to ensure postpartum women remain healthy and well-monitored during such challenging times.

## Supporting information

S1 Data Exel file with manuscript data. (CSV)
