# Supplementary material for: Early hospital discharge following delivery during wartime is not associated with increased rates of postpartum hospital referrals
Source: PLoS One. 2026 Oct 16;21(10):e0335688. doi: 10.1371/journal.pone.0335688 (PMC13674708; doi:10.1371/journal.pone.0335688)
Supplement: Supplementary file 1 [file pone.0335688.s002.docx]

**Early Hospital Discharge Following Delivery During Wartime is not Associated with Increased Rates of Postpartum Hospital Referrals**

**Reviewer Comments**

**A very interesting study on a subject less studied.**

**Written well and concise. Comprehensible.**

**I have 2 minor comments. I noticed a few misspelled words such as “holyday” and a few grammatical errors, therefore English editing can further improve the manuscript. Secondly, consider adding to the limitations that the external validity of the study is very limited due to the unique conditions that characterize a certain country, with a relatively small population, where military service and reserve military service are mandatory, leading to a high rate of recruitment among the general population as well as among medical staff. The study design may be irreproducible. Otherwise, a very enjoyable and educative article.**
