# Supplementary material for: Early hospital discharge following delivery during wartime is not associated with increased rates of postpartum hospital referrals
Source: PLoS One. 2026 Oct 16;21(10):e0335688. doi: 10.1371/journal.pone.0335688 (PMC13674708; doi:10.1371/journal.pone.0335688)
Supplement: Supplementary file 2 [file pone.0335688.s004.docx]

Date: Wednesday, June 11, 2025

To: **Ahmed Mohamed Maged, MD**

#### Academic Editor PLOS ONE

Re: PONE-D-24-57057 entitled:

Early Hospital Discharge Following Delivery During Wartime is not Associated with Increased Rates of Postpartum Hospital Referrals

PLOS ONE

Dear Dr. Ahmed Mohamed Maged,

We would like to thank you, the editor of PLOS ONE Journal, and the reviewers for considering our manuscript for PLOS ONE Journal and for your time and efforts to review this manuscript. We feel that your insightful comments and suggestions have helped to improve the quality of our manuscript.

We have addressed each comment in the following reply and have revised the manuscript accordingly. We hope that the revised version of the manuscript is considered suitable for publication in PLOS ONE Journal.

An itemized reply to the comments of the Reviewers is provided below.

An Exel file with the data is attached.

Yours sincerely,

Prof. Alina Weissmann-Brenner.

Reviewers’ comment:

**Reviewer #1:** *This study evaluates the impact of war on postpartum hospitalization and maternal outcomes in a unique setting where an active conflict coexists with a highly developed and advanced medical system. The study highlights operational adaptations in obstetric care, particularly the shortening of postpartum hospitalization, and assesses its implications for maternal health. While this research provides valuable insights into healthcare adjustments in crisis settings, several aspects require further scrutiny, particularly regarding the clinical significance of the reported hospitalization reduction, differentiation between low- and high-risk patients, and shifts in obstetric decision-making.
The study reports a 13.4% reduction in postpartum hospitalization, with a 9.4% decrease after vaginal delivery and a 23% decrease after cesarean section (p < 0.0001). However, in absolute terms, the reduction translates to a median of approximately 5 hours for vaginal births and 20 hours for cesarean sections. Given that postpartum length of stay in many high-resource settings, including Israel, is already longer than international standards, the clinical impact of this reduction remains questionable.*
 **Reply to comment:** We thank the reviewer for his/her favorable assessment of the manuscript. Indeed, some medical centers do offer postpartum discharge 24 hours following delivery, while others discharge postpartum patients following several days even after vaginal delivery.

Attached please find a google search regarding the hospitalization time in several medical centers. We believe that our study emphasizes the possibility to safely discharge postpartum patients earlier in specific cases such as national crisis.

*Clinical Relevance of Reduced Hospitalization Duration
The study does not define a threshold for what constitutes “short” hospitalization. Prior research, such as Bernstein et al. (2016) (PMC4783077), defined 24 hours as the threshold for short hospitalization and found that reducing postpartum length of stay to 36 hours did not increase maternal morbidity, provided that appropriate follow-up care was in place. In contrast, the median postpartum hospitalization in this study remained 49.3 hours after vaginal delivery and 68.6 hours after cesarean section, even during wartime.
While the reduction is statistically significant, it is unclear whether it reflects a meaningful clinical adaptation or a minor logistical adjustment that had little impact on patient outcomes. A more substantial decrease in hospitalization duration, such as a shift toward 24-hour postpartum discharge, would have raised concerns about potential gaps in maternal care. However, the observed reduction—while indicative of an operational shift—may not represent a major change in postpartum management, particularly when compared to global trends in early discharge.
Additionally, it remains uncertain whether this reduction was imposed as a hospital-wide mandate or was selectively applied to patients based on medical criteria. If all postpartum women were uniformly discharged earlier, including high-risk patients, this could have serious clinical implications. Conversely, if early discharge was limited to low-risk women with stable postpartum courses, the impact on maternal and neonatal health would likely be minimal. A subgroup analysis distinguishing low- vs. high-risk women would help clarify whether earlier discharge affected outcomes differently across patient populations.*
**Reply to comment:** We thank the reviewer for this comment. As pointed at the attached photo from google some hospitals discharge postpartum women 24 hour following delivery, while others discharge them following a few days. The present study emphasizes that it is safe to consider earlier postpartum discharge in specific conditions. The early discharge took place after careful medical examination.

We agree with the reviewer that the comparison between low risk patients and high- risk patients is interesting, therefore we intend to perform such a study.

*Changes in Cesarean Section Practices and Obstetric Decision-Making
The study also reports a significant reduction in elective cesarean sections during wartime (57% vs. 92% of total cesareans, p < 0.0001). This dramatic shift suggests a major change in obstetric decision-making, but the study does not explore the reasons behind it.
Several factors could explain this trend:
1. Resource Allocation: During wartime, hospital resources may have been reallocated toward emergency and trauma care, limiting the availability of scheduled elective surgeries, including cesareans.
2. Maternal Preferences: Women may have actively chosen to avoid elective cesareans due to concerns about prolonged hospitalization or potential disruptions in postnatal care.
3. Clinical Prioritization: Obstetric teams may have prioritized vaginal delivery over elective cesareans to reduce surgical burden and hospital stay.
While a shift away from elective cesareans might be expected in a crisis setting, the study does not examine whether this change had clinical consequences. Elective cesarean sections are typically performed under controlled conditions, allowing for better preparation and lower emergency risks. If wartime conditions led to an increase in non-elective or emergency cesareans, this could have implications for maternal and neonatal outcomes. The study should assess whether the reduction in elective cesareans led to an increase in labor complications, emergency deliveries, or neonatal distress.*
**Reply to comment:** We thank the reviewer for the comment. As requested, we added a discussion regarding the elective cesarean section. Our study found no significant differences in the rate of meconium, rate of low Apgar scores and the rate of admissions to the Newborn Intensive Care Unit between the wartime and the similar period in the previous year. Therefore, the shift seen in the rate of elective cesarean section had no clinical impact.

*Postpartum Emergency Room Visits and Alternative Care-Seeking Behavior
Despite the reduction in hospitalization duration, the study found no significant increase in postpartum referrals to the gynecological emergency room (GER) (8.7% vs. 8.8%). This is an important finding, as one might expect an increase in postpartum complications if early discharge led to inadequate follow-up care. The lack of a rise in GER visits suggests that women may have sought postpartum care in alternative settings rather than returning to the hospital.
Given Israel’s well-developed outpatient care system, it is plausible that postpartum women preferred to seek care in HMO clinics rather than returning to the hospital, particularly during wartime. The study does not examine whether there was a corresponding increase in postpartum clinic visits, which would support the hypothesis that care-seeking behavior shifted from hospital-based to community-based settings.*
**Reply to comment:** We thank the reviewer for this favorable comment.

**Reviewer #2:** *A very interesting study on a subject less studied.
Written well and concise. Comprehensible.
I have 2 minor comments. I noticed a few misspelled words such as “holyday” and a few grammatical errors, therefore English editing can further improve the manuscript. Secondly, consider adding to the limitations that the external validity of the study is very limited due to the unique conditions that characterize a certain country, with a relatively small population, where military service and reserve military service are mandatory, leading to a high rate of recruitment among the general population as well as among medical staff. The study design may be irreproducible. Otherwise, a very enjoyable and educative article.*

**Reply to comment:** We thank the reviewer for his/her favorable comment.

We corrected grammatical errors and added the suggested limitation to the Discussion section.

**Reviewer #3:** *The manuscript's validity and conclusions would be strengthened by addressing the following key issues:

1. Matching and Control: To ensure comparability between groups, the authors should employ matching techniques, such as propensity score matching, to balance cases and controls based on relevant covariates.
2. In-depth Statistical Analysis: Consultation with a statistician is recommended to perform a more comprehensive analysis, including multivariate models like logistic regression or Poisson regression, to account for potential confounders and draw robust conclusions.
3. Data Cleaning and Transparency: The methodology should include a detailed description of data cleaning processes, including handling of missing values, variable selection, and reasons for data exclusion.*
**Reply to comment:** We thank the reviewer for the comment. A multivariable

logistic regression model was performed as requested. Data was added to the Method section and to the Result section.

*Specific Suggestions for Improvement
1. Introduction: Enhance the introduction by citing relevant publications on healthcare during wartime, highlighting hospitalization needs, and providing statistics. Clearly articulate the research gap and objectives.*

**Reply to comment:** We added references as requested, and made clarification of the research gap and objectives as requested.

*2. Analysis: Explore potential confounders, such as age categories, occupation, residence, parity, comorbidities, and type of deliveries, and adjust for them in the multivariate analysis.*

**Reply to comment:** We thank the reviewer for the comment. A multivariable logistic regression model was performed as requested. Data was added to the Method section and to the Result section. We do not have information about the occupation of our patients. We agree that knowledge of the place of residence is important. However, because of the missile attacks on Israel about 64,000 Israelis were evacuated from their homes in the southern parts of Israel, and about 61,000 Israelis were evacuated from their homes in the northern parts of Israel. They all moved to temporary residence during the war. In the computerized data base we have information about the permanent residence. Because of the changes during the war this information may not be correct, therefore we did not include it in our analysis.

*3. Methodology: The authors should also provide context of the hospital and the location of the hospital*
**Reply to comment:** The Chaim Sheba Medical Center is a tertiary hospital in the center of Israel, that treated many wounded soldiers and civilians. Many of the hospital workers and/or their spouses were recruited to the army during the war. The hospital made many changes and adaptations in personnel and in equipment in all departments, including transfer of staff between departments and reinforcement of the departments according to the specific needs.

*Conclusion
By addressing these concerns and incorporating the suggested improvements, the manuscript can provide more robust insights into the impact of early hospital discharge during wartime on postpartum hospital referrals. With revisions, the study's validity and contribution to the field can be significantly enhanced.*

**Reply to comment:** We thank the reviewer for his/her comments.

**Reviewer #4:** Summary & Contribution:
This paper presents a timely and persuasive data-driven analysis of postpartum outcomes among women discharged early following delivery during wartime. The authors convincingly show that early discharge during emergency conditions—specifically war—did not lead to increased gynecological emergency room (GER) visits or readmissions. This is an important and novel contribution, particularly given the paucity of evidence on obstetric care adaptations during conflict scenarios. The work offers actionable insights for public health policy and emergency preparedness protocols in regions experiencing war or similar large-scale disruptions.

Strengths:
*Novelty and Relevance: To my knowledge, this is the first study examining early postpartum discharge outcomes during a wartime context, filling an important gap in obstetric literature.
Robust Dataset: The study uses electronic medical records from a large tertiary hospital, includes a sizable patient population (N=1,714), and compares wartime data with a well-matched control period.
Clear Methodology: The authors used appropriate statistical comparisons and provided well-structured tables that allow easy interpretation of the results.
Policy Implications: The findings are especially useful for informing emergency response protocols in healthcare systems during times of conflict.
Areas for Improvement and Suggestions:
Data Accessibility for Future Research:
While the paper states that data is available on request, I strongly recommend that a de-identified version of the dataset be made available in a public repository (e.g., Dryad or Figshare). This would greatly enhance transparency and facilitate future meta-analyses or cross-country comparisons.*
**Reply to comment:** We thank the reviewer for his/her favorable comments.

Data Visualization:
The authors could strengthen their argument by adding visual summaries, such as:
A boxplot comparing hospitalization durations by delivery mode (vaginal vs cesarean) across the two time periods.
A bar chart or stacked histogram of GER referral reasons by group (e.g., infections, bleeding).
A time-to-event curve (e.g., Kaplan-Meier) for GER visits or readmissions to assess timing distributions post-discharge.

**Reply to comment:** We thank the reviewer for the comment. A time to event curve (Kaplan Meyer) was added (Figure 1) as requested. Data was added to the Method section and to the Result section.

Clarification of Elective vs Non-Elective Cesarean Patterns:
*The sharp drop in elective cesareans (from 92% to 57%) is striking. While this is acknowledged, it would be valuable to include a short discussion on potential implications—e.g., patient autonomy, clinical risk-taking, or resource prioritization in wartime.*

**Reply to comment:** We thank the reviewer for his/her favorable comments. We added a discussion regarding the elective cesarean section as requested.

Discussion on External Validity:
The paper mentions generalizability due to population diversity. However, a short paragraph elaborating on how these findings might translate to low-resource or conflict-affected regions with weaker health infrastructure would enhance its utility for global health readers.

**Reply to comment:** We thank the reviewer for his/her comment. We added a paragraph in the Discussion section as requested.

Call for Further Research:
The authors suggest a nationwide study, which is appropriate. To guide this future work, they could briefly propose potential variables or methods (e.g., linking hospital discharge data with community midwife visits or primary care outcomes) to enhance comprehensiveness.

**Reply to comment:** We added data to the Method section with information about the Chaim Sheba Medical Center. We added limitation why the study is not reproducible in other countries to the Discussion section

Minor Edits:

The manuscript occasionally uses passive constructions ("data was retrieved...")—minor revisions for readability and active voice would improve flow.

Figure/table legends could be more descriptive for stand-alone clarity.

**Reply to comment:** We made grammar changes.

**Reviewer #5:** Early Hospital Discharge Following Delivery During Wartime is not Associated with Increased Rates of Postpartum Hospital Referrals

The topic is very interesting and addresses an issue that could hardly be addressed except in a state of exception such as war.

**Reply to comment:** We thank the reviewer for his/her favorable review.

Abstract: The abstract needs better contextualization about war and risk locations. It is necessary to consider the bias of patients not returning because readmission may not have occurred also due to access issues and risks inherent to war.

**Reply to comment:** We added information regarding changes and adaptations in hospitals during war, with the limitation of the 250 words allowed buy the PLOS ONE journal.

*The introduction is clear, but it could provide more data and information from studies on the topic, especially when dealing with states of exception such as floods, wars and the COVID period itself, which could serve as a comparison.*

**Reply to comment:** We added more references to the Introduction section. We added information about the lack of knowledge regarding shortening of postpartum hospitalization and its influence on postpartum visits to the gynecological emergency room during war.

*Regarding the methods, it would be interesting if the authors described the indicator related to infection after cesarean section compared between the groups.*

**Reply to comment:** We added definition of infection to the Method section.

The results are clear, but the tables are polluted and should be better adjusted.
The discussion is well-prepared and brings rich elements to discuss a new approach. I miss the text's approach to home strategies in the postpartum assessment period, with the aim of preventing patient readmission.

**Reply to comment:** We added a take home message to the Conclusion section as suggested.

*The references are adequate, but I miss historical references from the past about the text and its contextualization.*

**Reply to comment:** We added additional references as suggested.

Therefore, I consider the article interesting, but it should be adjusted with the recommendations made here to be eligible for publication.

**Reply to comment:** We thank the reviewer for his/her favorable review.

**Reviewer #6:** The topic studied by the authors is how the frequency of postpartum complications and other medical issues varies upon the shortening of hospitalization, in the context of the aftermath of a traumatic national event. Sadly, this is a worthwhile and relevant topic of study, which may also be applicable to other environments in which postpartum stays have been shortened due to adverse circumstances, such as active warzones.
The study design used by the authors involves comparing a data from a cohort of people who gave birth in the month after the dreadful October 7 attacks on Israel in 2023 with a cohort of people from the same area who gave birth in the same month of the previous year. The authors have treated the two groups are comparable and have not made statistical adjustments such as regression.
*A different study design would have been preferable to use. A pre-post study design in which data are compared from those who gave birth in the month before the attacks and the month after, which would likely reduce the effect from other confounders, relative to choosing the corresponding time period in the previous year as the comparator group.*

**Reply to comment:** We thank the reviewer for his/her favorable review. We chose to compare data to a similar period in the previous year due to the Jewish holiday that took place during the war. October 7^th^ 2023 was a Jewish holiday of SIMCHAT TORA. During holidays outpatient clinics are closed, and this may affect the referrals to the hospital. This is the reason we chose to make comparisons to a similar period from SIMCHAT TORA 2022, and not to the previous months, that included other Jewish holydays.

A method such as regression discontinuity or interrupted time series could then be used. Use of data from the two month-long periods, separated by a year, should have been accompanied by adjustments for possible differences in the cohort using appropriate statistical regression approaches and/or, a propensity score-based approach such as matching or IPTW, including additional data on appropriate factors that may affect the length of postpartum hospital stay.
Attention should also be given the description of the circumstances and environment of the individuals in the cohort under study.

**Reply to comment:** We thank the reviewer for the comment. A multivariable logistic regression model was performed as requested. Data was added to the Method section and to the Result section.

*I am uncomfortable with the use of the word “warzone” being applied to the area of Ramat Gan or even the greater Tel Aviv region (it seems to me as being implied) as it is 60-70 km from the nearest part of Gaza, which is unambiguously a warzone. The circumstances seem to me to be those of living in the traumatic aftermath of a terrorist with the reasonable worry of further attack rather than civilians being directly affected by war fighting (c.f. parts of Gaza). I could not find reports that this area was attacked (e.g. by rockets) in the month after October 7, 2023, and it had been, it is highly likely the authors would have (appropriately) mentioned this in the manuscript.*

**Reply to comment:** During the study period of October 7^th^ 2023 and November 5^th^ 2023 there were 44 **clusters** of missile attacks on the Tel Aviv area, making the Tel Aviv area a true warzone. This information was taken from the Home Front Command internet portal. We added this information to the Method section and the References section.

*Also, each sentence in lines 85-88 (second paragraph) requires a reference, to news articles or government statements if necessary. The use of the word “wartime” is also not without issue – the circumstances necessitating the shorter postpartum stays should be described using more generally applicable language. Accurate, and specific description of the cohort and relevant circumstances is important for any epidemiological study.*

**Reply to comment:** We thank the reviewer for this important suggestion. We added references 13-18.

We hope that the revised manuscript is accepted by PLOS ONE Journal. We will be happy to answer any further questions.

Sincerely,

Prof. Alina Weissmann-Brenner

Department of Obstetrics and Gynecology, The Chaim Sheba Medical Center, Tel

HaShomer
