# Supplementary material for: Early hospital discharge following delivery during wartime is not associated with increased rates of postpartum hospital referrals
Source: PLoS One. 2026 Oct 16;21(10):e0335688. doi: 10.1371/journal.pone.0335688 (PMC13674708; doi:10.1371/journal.pone.0335688)
Supplement: Supplementary file 3 [file pone.0335688.s006.docx]

1. Title and Abstract

The title is clear and accurately reflects the study's main finding. The abstract provides a concise summary of the background, methods, key results, and conclusion.

- However, the results section within the abstract is densely packed with statistical values, which can be challenging to read quickly. For instance, it states, "There were significantly less elective cesarean sections during wartime (57% vs. 92%; p<0.0001). The duration of postpartum hospitalization was significantly shorter during wartime [median length of 53.2 (IQR 42.4 – 72.4) hours vs. 64.1 (IQR 47.4 – 82.8) in the previous year (p<0.0001)]".
- To improve readability, the abstract could be streamlined by focusing on the primary outcomes. While the detail is valuable, presenting the most critical findings (e.g., no increase in GER referrals despite shorter stays) more prominently and perhaps simplifying the statistical presentation would enhance clarity.

2. Introduction

The introduction effectively sets the context by describing the challenges healthcare systems face during wartime, citing the specific events in Israel on October 7, 2023, and stating the study's objective. It correctly identifies a gap in the literature regarding the impact of early postpartum discharge during conflict.

- The section is well-written. A minor addition could be to briefly mention why early discharge is a standard practice even in non-wartime scenarios (e.g., COVID-19 pandemic, patient preference) to provide a broader context before narrowing down to the wartime situation. The paper does this later in the discussion, but a brief mention here could strengthen the rationale.

3. Methods

This section describes the retrospective study design, study periods, patient data sources, and variables assessed. It mentions that low-risk patients were offered early discharge, but it's unclear what criteria defined "low-risk" or what the acceptance rate of this offer was. The statistical methods, including the use of Chi-square, Wilcoxon, t-tests, and a multivariable logistic regression model, are appropriate for the data types.

- The manuscript should explicitly define the clinical criteria used to classify a patient as "low-risk" and eligible for early discharge. This is crucial for the study's reproducibility and for understanding the specific patient population.
- The rationale for choosing the specific dates for the control period (17.10.22-15.11.22) to align with Jewish holidays is sound. However, explicitly naming the relevant holidays could add clarity for an international audience.
- The definition of infection is described as "clinically and laboratory in patients with fever, chills, erythema in scar, purulent secretions, leukocytosis, etc." The "etc." is vague. The authors should specify all criteria used to define an infection to ensure transparency.

4. Results

The results are presented clearly, supported by two tables. Table 1 compares population characteristics, and Table 2 focuses on postpartum GER referrals. The finding that elective cesarean sections dropped significantly (57% vs. 92.5%) is highlighted. The primary outcome—no significant difference in GER referrals (8.7% vs. 8.8%) or readmissions (17% vs. 14%) despite shorter hospitalization—is clearly stated.

- The table and notes provide definitions for "Urgent" vs. "Non-Elective" Cesarean Sections categories. However, ensuring these are mutually exclusive and consistently applied is important. The dramatic increase in non-elective sections and decrease in elective sections is a key finding and warrants precise categorization.

5. Discussion

Analysis: The discussion effectively interprets the findings, contextualizing them with previous literature from the COVID-19 pandemic. It rightly points out the advantages of early discharge during war, such as reducing crowding and freeing up resources. It also thoughtfully analyzes the significant reduction in elective cesarean sections. The authors appropriately acknowledge the study's limitations, including its retrospective nature and the unique context of Israel, which may affect generalizability.

- The discussion is strong. It could be slightly enhanced by speculating on the potential role of enhanced community-based postnatal follow-up, if any, that might have supported the early discharge policy. If no formal changes were made, mentioning this would also be valuable information.

6. Conclusions

The conclusion is direct and aligns with the study's findings: shortening hospitalization for low-risk women after delivery in wartime is feasible and does not increase GER referrals. It appropriately suggests this could inform policy-making and emphasizes the need for careful patient communication.

- The conclusion is well-formulated and requires no significant changes. It is a concise summary of the study's key takeaway message.

Major revision

The "Results" section mentions that a multivariate analysis was performed, but it only describes the outcome in the text without a corresponding table. Specifically, the manuscript states: "Similarly, multivariable logistic regression showed no significant difference between the wartime and the previous year (OR = 1.01, 95% CI 0.72-1.41, p=0.98)."

The authors does describe how the model was fitted in the "Methods" section, stating that it was used to compare post-partum visits to the GER while controlling for potential confounders.

- However, the detailed output of this analysis is not displayed in a table format, unlike the descriptive statistics in Table 1 and the clinical outcomes in Table 2. Those the results were not significant, I suggest the output should still be presented

Minor revision

- The manuscript presents conflicting information on data availability. The formal review checklist and a data availability statement claim that all data is fully available within the paper and its supporting files. In contrast, the "Declarations" section states the data is not publicly available due to privacy/ethical restrictions and can only be obtained upon request from the corresponding author. This inconsistency needs to be resolved by the authors to provide clear and accurate information on data access.
