# Supplementary material for: Early hospital discharge following delivery during wartime is not associated with increased rates of postpartum hospital referrals
Source: PLoS One. 2026 Oct 16;21(10):e0335688. doi: 10.1371/journal.pone.0335688 (PMC13674708; doi:10.1371/journal.pone.0335688)
Supplement: Supplementary file 4 [file pone.0335688.s008.docx]

Date: Tuesday, January 13^th^, 2026

To: **Ahmed Mohamed Maged, MD**

#### Academic Editor PLOS ONE

Re: PONE-D-24-57057 entitled:

Early Hospital Discharge Following Delivery During Wartime is not Associated with Increased Rates of Postpartum Hospital Referrals

PLOS ONE

Dear Dr. Ahmed Mohamed Maged,

We would like to thank you, the editor of PLOS ONE Journal, and the reviewers again for considering our manuscript for PLOS ONE Journal and for your time and efforts to review this manuscript.

We have addressed each comment in the following reply and have revised the manuscript accordingly. We hope that the 4^th^ revised version of the manuscript is considered suitable for publication in PLOS ONE Journal.

An itemized reply to the comments of the Reviewers is provided below.

Yours sincerely,

Prof. Alina Weissmann-Brenner.

**Reviewers' comments:**
1. If the authors have adequately addressed your comments raised in a previous round of review and you feel that this manuscript is now acceptable for publication, you may indicate that here to bypass the “Comments to the Author” section, enter your conflict of interest statement in the “Confidential to Editor” section, and submit your "Accept" recommendation.

Reviewer #3: All comments have been addressed

Reviewer #7: All comments have been addressed

Reviewer #8: (No Response)

Reviewer #9: All comments have been addressed

**Reply to comment:** We thank the reviewers.

2. Is the manuscript technically sound, and do the data support the conclusions?

The manuscript must describe a technically sound piece of scientific research with data that supports the conclusions. Experiments must have been conducted rigorously, with appropriate controls, replication, and sample sizes. The conclusions must be drawn appropriately based on the data presented.

Reviewer #3: Yes

Reviewer #7: Yes

Reviewer #8: Partly

Reviewer #9: No

**Reply to comment:** We thank the reviewers.

3. Has the statistical analysis been performed appropriately and rigorously?

Reviewer #3: Yes

Reviewer #7: No

Reviewer #8: Yes

Reviewer #9: No

**Reply to comment:** We thank the reviewers.

4. Have the authors made all data underlying the findings in their manuscript fully available?

The [PLOS Data policy](http://track.editorialmanager.com/CL0/http:%2F%2Fwww.plosone.org%2Fstatic%2Fpolicies.action%23sharing/1/010f019add84b2dc-6b8a7afc-c891-45ac-9587-f6925e3e344d-000000/aIQe2DRXtKnjyf17S29lcErmM6st0rhoLAIJxoijvmc=239) requires authors to make all data underlying the findings described in their manuscript fully available without restriction, with rare exception (please refer to the Data Availability Statement in the manuscript PDF file). The data should be provided as part of the manuscript or its supporting information, or deposited to a public repository. For example, in addition to summary statistics, the data points behind means, medians and variance measures should be available. If there are restrictions on publicly sharing data—e.g. participant privacy or use of data from a third party—those must be specified.

Reviewer #3: No

Reviewer #7: (No Response)

Reviewer #8: No

Reviewer #9: (No Response)

**Reply to comment:** We thank the reviewers.

5. Is the manuscript presented in an intelligible fashion and written in standard English?

PLOS ONE does not copyedit accepted manuscripts, so the language in submitted articles must be clear, correct, and unambiguous. Any typographical or grammatical errors should be corrected at revision, so please note any specific errors here.

Reviewer #3: Yes

Reviewer #7: Yes

Reviewer #8: Yes

Reviewer #9: (No Response)

**Reply to comment:** We thank the reviewers.

6. Review Comments to the Author

Please use the space provided to explain your answers to the questions above. You may also include additional comments for the author, including concerns about dual publication, research ethics, or publication ethics. (Please upload your review as an attachment if it exceeds 20,000 characters)

Reviewer #3: This manuscript seems okay for submission as the author's have attended to the comments as seen from the abstract.I don't have access to the rest of the manuscript.

**Reply to comment:** We thank the reviewers.

Reviewer #7: Dear Editor,
I write to submit my review on the manuscript titled “Early Hospital Discharge Following Delivery During Wartime is not Associated with Increased Rates of Postpartum Hospital Referrals.”
The study compared the rate of postpartum visits to the gynecological-emergency-room (GER) between wartime to a similar period in the previous year.
Here are my comments
Methods: What was the entire duration of the war? This must be clearly stated in the manuscript. Did the war only last for one month? Why did the authors decide to only compare outcomes within just one month of war? This could explain why no significant differences are seen in most of the outcomes. There may be a lagged effect of war, that is, the true effect of the war may even be experienced post-war. Since authors are only analyzing secondary data, a sensitivity analysis could have been conducted by expanding the scope to include 6 months before and 6 months after the war to assess whether the effect would be the same as evaluating it within one month. The reasoning behind selecting one month as the duration of assessment should be clearly elaborated in the manuscript if further analyses cannot be performed

**Reply to comment:**

The war started with a surprise attack launched by Hamas on Saturday morning, Simchat Torah, October 7, 2023. In this attack, Hamas terrorists invaded IDF bases and civilian localities in the Gaza perimeter region, while firing thousands of missiles at Israel. About a day after the outbreak of the war, the Hezbollah terrorist organization fired missiles from Lebanon to the northern part of Israel. Iran also launched missile attacks on Israel on 04/24, 10/24 and 06/25. Ceasefire was achieved on 19/01/25.

On October 2023 there were 8500 missiles that were launched towards Israel, on November 2023 there were 2000missiles, and on December 2023 there were 2000 missiles launched towards Israel.

We chose to concentrate on the first month of the war mainly because of the surprising nature of this war, necessitating rapid adaptations of the hospitals, and the intensity of the missile attacks in that month. This data was added to the Introduction, methods and Discussion sections.

Indeed, additional adaptations were made during the missile attacks from Iran, including transfer of the delivery rooms to underground secure locations. However, the main focus of the present study was to assess the shortening of the duration of hospitalization following delivery, that was relevant mainly in the first month of the war.

Methods: Kindly define low-risk patients
Methods: The primary exposure of this study is war ( a binary indicator, before coded 0 and after war coded 1). Please include a section that outlines the outcome measures of interest and their corresponding measurement scales (e.g., continuous, binary, count, nominal, multinomial). Another section, called 'confounders, mediators,' should be clearly labeled. This will help assess whether the statistical methods chosen were appropriate. The variables are lumped together, including the sociodemographic factors which I believe are potential confounding factors.

**Reply to comment:** The section about the variables assessed in the study was changed as requested.

Methods: The section titled “Descriptive statistics and analysis” are all analyses. Kindly title that section “Statistical Analysis”. Descriptive and inferential statistics are part of statistical analysis.
Methods: The statement “In order to compare post-partum visits to the GER in the two periods while controlling for potential confounders, a multivariable logistic regression model was fitted to the data, with age at delivery, gestational week (below or above 37), fetus 137 count (one or more), number of previous births (1,2,3,4, 5+)”. The statement looks incomplete. That is ...... with age at delivery, gestational week (below or above 37), fetus 137 count (one or more), number of previous births (1,2,3,4, 5+) as what? Confounders

**Reply to comment:** Correction was made. Thank you.

?. Kindly check
Methods: The methods sound a bit confusing. Some outcomes were treated as time to an event, “time until a post-partum referral,” and the authors compared the distribution of survival time (time until a post-partum referral) using the log-rank test after the Kaplan-Meier curve. Ideally, the Cox proportional hazard model would have been fitted to determine the hazard of war on postpartum referral, controlling for other potential confounders. The odds ratio from the multivariable logistic regression model could then serve as a sensitivity analysis. But the Cox proportional hazard model was not fitted.

**Reply to comment:** We thank the reviewer. Changes were made in the Method section as requested.

Results: Table 1: Some of the expected frequencies were less than one (No. of fetuses vs wartime status) but reported p-values from the Chi-square test of independence instead of Fisher’s exact test.

**Reply to comment:** A footnote was added to Table 1 about the comparison for number of fetuses as requested.

Table 2: Must be restructured. Very confusing. Is it 70(8.7%) and 80(8.8%). Creating another column for the percentages makes it difficult to understand. No need.

**Reply to comment:** We thank the reviewer. Changes were made in the Results section as requested. The columns in Table 2 were changed.

Reviewer #8: (No Response)

Reviewer #9: Comments.
*Title
* Running title
*Abstract
- All abbreviations should have been provided in full on first mention and this applies to the title, running (short) title, abstract, impact statement, main text and each table / figure independently as they will be read independently. Please use the abbrevations correctly and effectively so all the abbrevations should be checked.

**Reply to comment:** We thank the reviewer. We explained the abbreviations in the Abstract, Declaration and Results sections as requested.

- The sstudy groups should be defined and also should be given the number of the participants for each group.

**Reply to comment:** 801 women delivered in the first month of the war in 2023 and 913 delivered in the parallel period in the previous year.

- Please do not to start to sentences with figures or numbers.

**Reply to comment:** Changes were made as requested.
- Please determine the statistically significant of p values (p<0.05 or p<0.001 or p<0.0001).
- The real p values should be given.

**Reply to comment:** Due to limitations of the number of words in the Abstract section we cannot add the sentence that p value of <0.05 was considered statistically significant. This sentence appears in the method section.
- The abstract must contain enough data (figures) to enable the reader (researcher) to judge its content and therefore relevance. It can not include undefined terms or contradictory statements.

**Reply to comment:** Tables and Figure are embedded in the manuscript itself. If the Editor wishes we provide an Abstract with Figures we can changes the entire Abstract as requested.
- The manuscript should have ended with a clear conclusion that sums up the value of the study within its limitations.

**Reply to comment:** The Abstract ends with the conclusions of the manuscript. Due to limitations of the word count of the Journal the Limitations were specified in the Discussion section.
*Keywords
- The keywords should be added according to alphabetical order and they should be compatible with MeSH.

**Reply to comment:** We arranged the Key Words in alphabetical morder.

* Introduction
- An introduction should include information on why the subject under investigation is important, what is currently known on the topic and how the current investigation will improve our knowledge.
**Reply to comment:** The Introduction section was editted as requested.

*Materials and Methods
- Statistical analysis method should be detailed. "Which test used for to determine distribution?, which test used for normal distribution and non-normal distribution. (Ideally, the distribution type and the statistical test used should be specified for each parameter). Kolmogorov-Smirnov test should be used to determine the distribution of the variables.
- The parameters distributed with normally distribution t test should be used, the parameters distributed with non-normally distribution should be used Mann-Whitney U test should be used.
- Which tests were used for categorical variables? The conditions for a valid chi-squared test and the circumstances when Fisher’s exact test can be used should have been specified in the method as well.
- The parameters distributed with normally distribution should be explained Mean+SD, the parameters distributed with non-normally distribution should be explained Median (min-max) or ideally median (25th-75th percentiles).
- Multivariate analysis should be performed to exclude possible confounding fctors.

*Results
- This section must contain enough data (figures) to enable the reader (researcher) to judge its content and therefore relevance. It can not include undefined terms or contradictory statements.

**Reply to comment:** The Result section contains data of the results of the study including statements and Tables and Figure.

*Tables and Figures

* Discussion
- Practical implications and future research direction are not mentioned.
- In the discussion the authors should summarise and qualify their findings, compare outcomes with other publications and draw conclusion. The implications of the study and the future direction of study should be discussed. Importantly the study’s limitations should be considered.
- The manuscript should have ended with a clear conclusion that sums up the value of the study within its limitations.

**Reply to comment:** Discussion and conclusion sections were edited and changed as requested, including English corrections.

- Post-hoc power analysis should be performed to exclude small sample size as a limitation.

**Reply to comment:** Due to the retrospective nature of the study we did not perform a power analysis. Explanations about why the first 2 months of the war were chosen are explained in details in both manuscript and in the response to the reviewer's previous questions.

*References
- The references should be cited the articles published in the last five years to progress the science.

**Reply to comment:** There is lack of data regarding the subject of the manuscript, therefore references before 2020 were also added to the manuscript.

***** All abbreviations should have been provided in full on first mention and this applies to the title, running (short) title, abstract, impact statement, main text and each table / figure independently as they will be read independently. Please use the abbrevations correctly and effectively so all the abbrevations should be checked.

**Reply to comment:** Abbreviations were specified as requested.

***** Language and presentation require improvement.

***** Need several grammar/wording corrections (There are a lot of words with no space between them, there are missing spaces after commasin the manuscript and also, there are a lot of gramatical error. Language could be improved).

**Reply to comment:** Changes were made as suggested.
