# Supplementary material for: Early hospital discharge following delivery during wartime is not associated with increased rates of postpartum hospital referrals
Source: PLoS One. 2026 Oct 16;21(10):e0335688. doi: 10.1371/journal.pone.0335688 (PMC13674708; doi:10.1371/journal.pone.0335688)
Supplement: Supplementary file 5 [file pone.0335688.s009.docx]

Date: Thursday, February 19, 2026

To: **Ahmed Mohamed Maged, MD**

#### Academic Editor PLOS ONE

Re: PONE-D-24-57057 entitled:

Early Hospital Discharge Following Delivery During Wartime is not Associated with Increased Rates of Postpartum Hospital Referrals

PLOS ONE

Dear Dr. Ahmed Mohamed Maged,

We would like to thank you, the editor of PLOS ONE Journal, and the reviewers again for considering our manuscript for PLOS ONE Journal and for your time and efforts to review this manuscript.

We have addressed each comment in the following reply and have revised the manuscript accordingly. We hope that the 5^th^ revised version of the manuscript is considered suitable for publication in PLOS ONE Journal.

An itemized reply to the comments of the Reviewers is provided below.

Yours sincerely,

Prof. Alina Weissmann-Brenner.

**Reviewers' comments:**

1. If the authors have adequately addressed your comments raised in a previous round of review and you feel that this manuscript is now acceptable for publication, you may indicate that here to bypass the “Comments to the Author” section, enter your conflict of interest statement in the “Confidential to Editor” section, and submit your "Accept" recommendation.

Reviewer #7: All comments have been addressed

Reviewer #8: All comments have been addressed

Reviewer #9: All comments have been addressed

**Reply to comment:** We thank the reviewers.

2. Is the manuscript technically sound, and do the data support the conclusions?

The manuscript must describe a technically sound piece of scientific research with data that supports the conclusions. Experiments must have been conducted rigorously, with appropriate controls, replication, and sample sizes. The conclusions must be drawn appropriately based on the data presented.

Reviewer #7: Yes

Reviewer #8: Yes

Reviewer #9: No

**Reply to comment:** We thank the reviewers.

3. Has the statistical analysis been performed appropriately and rigorously?

Reviewer #7: Yes

Reviewer #8: Yes

Reviewer #9: No

**Reply to comment:** We thank the reviewers. A post hoc power analysis was made. Data was added to the Method and the Discussion sections.

4. Have the authors made all data underlying the findings in their manuscript fully available?

The [PLOS Data policy](http://track.editorialmanager.com/CL0/http:%2F%2Fwww.plosone.org%2Fstatic%2Fpolicies.action%23sharing/1/010f019c5241f533-38cd72dd-ec34-4e9a-910e-28ef399d0924-000000/rxwwezHhZezCtkxZFkJd3v1OBivwLm-dOBI6kv7r_KA=250) requires authors to make all data underlying the findings described in their manuscript fully available without restriction, with rare exception (please refer to the Data Availability Statement in the manuscript PDF file). The data should be provided as part of the manuscript or its supporting information, or deposited to a public repository. For example, in addition to summary statistics, the data points behind means, medians and variance measures should be available. If there are restrictions on publicly sharing data—e.g. participant privacy or use of data from a third party—those must be specified.

Reviewer #7: Yes

Reviewer #8: Yes

Reviewer #9: No

**Reply to comment:** We thank the reviewers.

5. Is the manuscript presented in an intelligible fashion and written in standard English?

PLOS ONE does not copyedit accepted manuscripts, so the language in submitted articles must be clear, correct, and unambiguous. Any typographical or grammatical errors should be corrected at revision, so please note any specific errors here.

Reviewer #7: Yes

Reviewer #8: Yes

Reviewer #9: No

**Reply to comment:** We thank the reviewers.

6. Review Comments to the Author

Please use the space provided to explain your answers to the questions above. You may also include additional comments for the author, including concerns about dual publication, research ethics, or publication ethics. (Please upload your review as an attachment if it exceeds 20,000 characters)

Reviewer #7: The authors have comprehensively addressed all the comments and concerns raised in my previous review of the manuscript

Reviewer #8: (No Response)

Reviewer #9: The artcile was not revised accordingly.
- Post-hoc power analysis should be performed to exclude small sample size as a limitation.

**Reply to comment:** We thank the reviewers. A post hoc power analysis was made. Data was added to the Method and the Discussion sections.
