# Supplementary material for: Early hospital discharge following delivery during wartime is not associated with increased rates of postpartum hospital referrals
Source: PLoS One. 2026 Oct 16;21(10):e0335688. doi: 10.1371/journal.pone.0335688 (PMC13674708; doi:10.1371/journal.pone.0335688)
Supplement: Supplementary file 6 [file pone.0335688.s010.docx]

Date: Wednesday, June 3, 2026

To: Danish Ahmad, , MBBS,MSc,MNAMS,PhD,IP-H(UK),FRCP(Edin),FRCP(Lon)

Academic Editor
PLOS ONE

Re: PONE-D-24-57057 entitled:

Early Hospital Discharge Following Delivery During Wartime is not Associated with Increased Rates of Postpartum Hospital Referrals,

Dear Dr. Danish Ahmad,

We would like to thank you, the editor of PLOS ONE Journal for considering our manuscript for PLOS ONE Journal and for your time and efforts to review this manuscript.

We have addressed each comment in the following reply and have revised the manuscript accordingly. We hope that the 6^h^ revised version of the manuscript is considered suitable for publication in PLOS ONE Journal.

An itemized reply to the comments of the Reviewers is provided below.

Yours sincerely,

Prof. Alina Weissmann-Brenner.

**Reviewers' comments:**

**Overview**
The manuscript addresses a clinically and policy-relevant question whether the introduction of an early-discharge protocol during wartime altered the rate or pattern of postpartum gynaecological emergency room (GER) presentations. The study design is sound in its core elements: the holiday-shifted control window is a thoughtful bias control, the mediator/confounder distinction in the model specification is correctly handled in principle, and the analytical strategy of logistic regression with a Cox proportional-hazards sensitivity analysis is appropriate for fixed-window follow-up data. The authors should be commended on these elements.
That said, several issues remain after the previous round. The comments below identify gaps in framing, language, cohort description, confounder selection, interpretation of borderline findings, and reporting standards. A number of these are substantive and, in our view, must be addressed before the manuscript is suitable for publication.

Introduction

1. Lines 75–93 focus the opening and disambiguate "war" from "disaster". These lines read as a broad scene-setting passage and do not introduce the study's obstetric/maternal-health focus early enough. Please open with a more focused topic sentence, and delete lines 75–83. A related issue runs through the manuscript: war and disaster are not interchangeable terms  they have distinct definitions and produce different health system impacts. As the study is set in a conflict context rather than a natural-disaster context, please use war / armed conflict terminology consistently throughout.

**Reply to comment:** We thank the reviewers. These lines were deleted as requested. Anew paragraph was added to the Introduction section as requested:

Armed conflicts place exceptional strain on healthcare infrastructures, compelling both institutions and practitioners to swiftly adapt in order to continue providing effective care amid uncertainty and limited resources. Common responses to these pressures include reallocating medical staff, modifying patterns of patient referral and hospitalization to accommodate surges of injured military personnel and civilians, and reorganizing hospital operations to balance urgent demands with routine healthcare services [1–11]. One frequent strategy employed during wartime is the reduction of hospital length of stay for select groups, such as low-risk postpartum women. This approach is intended to maximize bed availability and minimize patients’ exposure to conflict-related risks. However, the potential impacts of shortened hospitalization on patient outcomes within this context remain insufficiently understood.

2. Lines 110–112 relocate or delete. These lines sit awkwardly in the introduction. Their generalising claim that the findings may be relevant to conflicts and disasters more broadly belongs in the discussion or conclusion, after the specific findings have been presented. Please consider deleting these lines from the introduction or relocating them.

**Reply to comment:** These lines were deleted as requested.

3. Introduce Israel’s health-system context early, not in the limitations. A point currently raised only in the limitations should be introduced in the introduction or early methods: Israel is a high-income setting with mature emergency and trauma care infrastructure, shaped both by repeated exposure to armed conflict and by substantial domestic investment in trauma medicine. Establishing this context early frames the generalisability of the findings appropriately for an international readership.

**Reply to comment:** The data was added to the Introduction as requested.

4. Lines 94–98, 119–120, and 298–299 adopt objective scientific language and add specific contextual anchors. These lines describe the events of 7 October and the resulting loss of life. While the gravity of those events is fully acknowledged, a scientific manuscript for an international audience should present this background in objective language, without evaluative adjectives and without details that do not bear directly on the study aim or methods. Elements such as the regional evacuation are worth retaining because they describe a population-level shift relevant to the analysis. The authors should also state explicitly that, following the attack on 7 October, Israel formally declared a state of war on 8 October, and that the present study covers only the first month of that period.

**Reply to comment:** Changes were made as requested:

On October 7, 2023, Hamas organization launched a war against Israel including massive missile attacks and territory invasion, resulting in over 1,400 lives lost and the abduction of 251 hostages. On October 8^th^ 2023 Hezbollah subsequently joined, launching rockets at Israel, including attacks targeting civilian populations, leading to declaration of war by Israel.

5. Clarify the scope of the early-discharge protocol. Please clarify whether the early-discharge protocol described in the methods is specific to the study centre, or whether it reflects a national directive issued in response to the war declaration. This affects how readers will interpret generalisability.

**Reply to comment:** During that period the Israeli Ministry of Health revised its guidelines and permitted hospitals to significantly shorten the length of postpartum hospitalization. This data was added as requested to the Method section.

6. Outcome ascertainment and post-discharge follow-up. Reviewer 1 raised this in the previous round and the response was not, in our view, sufficient. Please clarify (a) whether discharged women received structured follow-up for example, telephone contact or written instructions identifying warning signs and points of presentation — and (b) how the authors are confident that women experiencing complications were captured by the study, given they could plausibly have presented to emergency departments outside the study site (particularly relevant in the context of regional evacuations). If outside-site presentations cannot be ascertained from the available data, this must be stated explicitly as a limitation affecting outcome ascertainment.

**Reply to comment:** a) Each postpartum woman received detailed explanations from both an obstetrician and a maternity nurse, as well as a discharge letter specifying when to seek medical attention (for example, in cases of fever, bleeding, abnormal discharge, unusual appearance of the surgical scar, etc).

b) We do not have data regarding whether women who were discharged sought care at other medical centers following delivery. However, not all clinics were operational during the war. Given that our hospital is a tertiary care center located in central Israel—and not in the north or south, where missile attacks were significantly more frequent—we assume that if a postpartum woman required medical attention, she would have presented to our facility. This data was added to the Limitation section in the Discussion section.

Methods and Results
7. Table 2 interpretation of time from delivery to GER referral. The reported time from delivery to GER referral appears shorter in the wartime cohort (median 5.9 [IQR 4.5–10.6]) than in the control (median 7.5 [IQR 4.5–16.0]), with a Wilcoxon p-value of 0.16, yet the text states there was no significant difference in the time elapsed from delivery to postpartum referral. The point estimate is approximately 1.6 days earlier presentation in the wartime cohort, with a meaningfully wider tail in the control cohort. This is precisely the pattern that would be expected if early-discharged women re-present sooner because they are sent home before the natural window for early postpartum complications has closed. Characterising this as "no significant difference" obscures a clinically interpretable signal. Please:
(a) confirm whether the values reported are means or medians, with corresponding SD or IQR, and report the exact test used and p-value;
(b) discuss whether the apparent ~1.6-day earlier presentation, even if not statistically significant, is clinically meaningful  that is, whether it represents a signal that women discharged earlier under the wartime protocol required postpartum care sooner than the control cohort.
A non-significant p-value with a clinically suggestive point estimate warrants discussion rather than dismissal.

**Reply to comment:** We thank you for your comment.

a) The values reported are indeed median.

b) We added the information regarding the 1.6 days difference between early discharge to the control group to the Result section and the Discussion section.

8. Confounders and mediators  terminology. The terms confounder and mediator are used inconsistently across the manuscript. These are distinct epidemiological constructs and should not be used interchangeably. The methods correctly identify delivery characteristics and length-of-stay as mediators on the causal pathway, but elsewhere in the text the language slips. Please audit the manuscript for consistency and tighten it throughout.

**Reply to comment:** thank you for the useful comment. We double checked the phrasing and made sure that delivery characteristics are referred to as mediators,

and mother's characteristics (age at delivery, gestational week, fetus count, and number of previous births) are referred to as confounders and therefore are adjusted for in the multivariable model.

9. Discuss the shift in the elective caesarean fraction (Table 1). Table 1 reports that 57% of caesarean sections in the wartime cohort were classified as elective, compared with 92% in the control period (p < 0.0001). This 35-percentage-point shift is the single largest difference between the two cohorts and is currently mentioned only in passing. Three interpretations are possible and have substantially different implications for how the null finding on GER referral should be read:
(a) Elective caesareans were rationally deferred during wartime for resource or scheduling reasons, a parallel intervention, separate from early discharge.
(b) The wartime cohort experienced a genuine increase in obstetric complications, driving non-elective and urgent caesareans  an effect of war that should be reflected in other outcomes;
(c) The population delivering during wartime differed from the control population due to regional evacuations or altered referral patterns,  a case-mix shift that the current confounder set does not capture.
The authors are asked to discuss this finding explicitly in the manuscript

**Reply to comment:** The following paragraph was added to the Discussion section:

An interesting finding in the present study is the significant reduction in elective cesarean sections during wartime (57% vs. 92% of total cesareans, p < 0.0001). This sharp decline may reflect significant shifts in obstetric decision-making, perhaps influenced by patients' concerns over extended hospitalization and potential disruptions in postnatal care. Another possible explanation is a problem of resources or scheduling reasons. However, our hospital is a tertiary medical center, and the changes in personnel did not result in cancellations of elective cesarean sections. Another possibility may be that the population delivering during wartime differed from the control population due to regional evacuations or altered referral patterns. A nationwide study is required. An alternative explanation may be that the war caused an increase in obstetrical complications increasing the rates of non-elective and urgent cesarean sections. Despite the decline in elective cesarean sections, there were no significant differences in neonatal outcomes, including rates of meconium-stained amniotic fluid, low Apgar scores or rate of admissions to the Newborn Intensive Care Unit indicating that this strategic change did not adversely affect neonates.

10. Cohort derivation is not described and should be reported in STROBE-compliant form. The methods state that all deliveries within each study window were included, but the manuscript provides no inclusion/exclusion algorithm, no flow diagram, and no information on exclusions for fetal demise, antenatal transfer, incomplete electronic medical record data, or other reasons. STROBE adherence is not stated. For an observational cohort study using administrative data, this is a substantial reporting gap. Please provide a STROBE flow diagram and a clear inclusion/exclusion statement, and add a STROBE adherence statement to the methods.

**Reply to comment:**

The present study included all deliveries during the study period. No exclusions were made. Since all data regarding deliveries in our medical center is stored in a computerized database, we have complete and comprehensive information about each delivery, with no missing data. This data was added to the Method section.

The study was conducted in accordance with the Strengthening the Reporting of Observational Studies in Epidemiology (STROBE) guidelines. This information was added to the Method section.

11. Specify the operational definition of "low-risk" and consider an as-treated analysis. The definition of patients eligible for early discharge "generally healthy women, with no delivery complications such as profound vaginal bleeding, hypertension or infection"  is clinical rather than operational. The manuscript does not specify (a) the criteria that determined eligibility for early discharge in practice, (b) whether pre-existing maternal conditions (diabetes, mental health history, BMI) affected eligibility, or (c) what proportion of the wartime cohort actually received the abbreviated stay. This matters because the analysis treats the entire wartime cohort as exposed to the early-discharge intervention, when in reality only a subset would have been eligible. Please specify the operational criteria, report the proportion of each cohort that received early discharge, and present an as-treated sensitivity analysis restricted to women who actually received the abbreviated stay.

**Reply to comment:**

We thank you for these thoughtful and detailed comments.

We would like to clarify that the primary aim of our study was to evaluate the overall impact of reduced postpartum hospitalization duration on GER visits, rather than focusing on specific groups, providing a comprehensive overview of the clinical outcomes following policy implementation. Our intension was to evaluate whether the system-wide change in discharge policy was associated with a significant increase in postpartum GER visits in the general population. As our results demonstrate, we did not observe an increase in the frequency of such visits across the entire cohort, suggesting that the policy did not adversely affect this outcome in the population scale. We recognize the value of a more granular analysis. Indeed, future studies are warranted to explore the effects of shortened postpartum hospitalization on emergency department visits among various sub populations, such as women with elevated BMI, diabetes, mental health disorders and other relevant comorbidities. Such research could provide important insight into the different impact of early discharge policies on maternal health outcomes. We recommended in the Discussion section to perform in a future study a comparison between low and high-risk pregnancies.

Here are three articles addressing the shortening of hospitalization among postpartum women during the COVID-19 pandemic. Similar to our study, these articles also examined the entire population within their respective hospitals:

- [Early postpartum discharge during the COVID-19 pandemic.](https://pubmed.ncbi.nlm.nih.gov/32845868/)

Bornstein E, Gulersen M, Husk G, Grunebaum A, Blitz MJ, Rafael TJ, Rochelson BL, Schwartz B, Nimaroff M, Chervenak FA.J Perinat Med. 2020 Nov 26;48(9):1008-1012. doi: 10.1515/jpm-2020-0337.PMID: 32845868.

- [Length of Postpartum Hospital Stays During COVID-19: Findings From a Convergent Parallel Mixed-Methods Study.](https://pubmed.ncbi.nlm.nih.gov/39154662/)

Correia RH, Greyson D, Carruthers A, Kuyvenhoven C, Kirkwood D, Jones A, Howard M, Darling E, Davis A, McDonald SD, Mniszak C, Kandasamy S, Vanstone M.J Obstet Gynaecol Can. 2024 Oct;46(10):102637. doi: 10.1016/j.jogc.2024.102637. Epub 2024 Aug 21.PMID: 39154662.

- [The Risk of Readmission after Early Postpartum Discharge during the COVID-19 Pandemic.](https://pubmed.ncbi.nlm.nih.gov/34891201/)

Gulersen M, Husk G, Lenchner E, Blitz MJ, Rafael TJ, Rochelson B, Chakravarthy S, Grunebaum A, Chervenak FA, Fruhman G, Jones MF, Schwartz B, Nimaroff M, Bornstein E.Am J Perinatol. 2022 Mar;39(4):354-360. doi: 10.1055/s-0041-1740061. Epub 2021 Dec 10.PMID: 34891201.

12. Reasons-for-GER analysis classifies only a minority of referrals. The analysis of indications for postpartum GER referral classifies only infection (11 + 13 = 24 cases) and bleeding (0 + 4 = 4 cases), totalling 28 of the 150 GER referrals  approximately 19% of all cases. The remaining ~120 referrals are not categorised. The statement that "there were also no significant differences in the reasons for the postpartum referrals" cannot be supported when the majority of referrals have not been classified. Please either (a) provide a complete categorisation of all GER referrals by indication, with appropriate adjustment for multiple comparisons, or (b) remove this analysis from the manuscript.

**Reply to comment:** Table 2 was erased. A paragraph about the postpartum visits and hospitalization was added to the Result section.

13. The confounder set is thin for a wartime/peacetime comparison. The multivariable models adjust for age at delivery, gestational week (<37 vs ≥37), fetus count, and parity. The exclusion of delivery characteristics and length of stay on the grounds that they are mediators is appropriate. However, several variables that are plausibly associated with both calendar period and the outcome, and which are not on the mediator pathway, have been omitted without justification:
(a) Maternal comorbidities eg. pre-existing hypertension, diabetes, obesity, thyroid disease, and mental health history. These conditions pre-date the exposure window and are established predictors of postpartum complications and ED re-presentation.
(b) Geographic and socioeconomic variables area of residence, distance to the index hospital, and any indicator of displacement status. Specifically in the wartime cohort, internal displacement following regional evacuations is a likely source of confounding that the current model cannot address.
Please explicitly acknowledge in the limitations the residual confounding that remains if these variables are not available in the dataset.

**Reply to comment:** We thank you for these insightful comments. As clarified, the primary objective of our study was to evaluate the overall impact of the early discharge policy on postpartum visits to the GER, taking a population-level approach akin to prior studies conducted during the COVID-19 pandemic. While we agree that maternal comorbidities and geographic or socioeconomic factors are important potential confounders, the capacity to adjust for these variables was limited in the context of this unique wartime event.

Specifically, large-scale evacuations from both northern and southern regions of Israel during the war rendered residential address and related geographic or socioeconomic indicators largely unreliable for analysis, as most evacuees lost their homes and ceased employment, greatly challenging the accurate assessment of these variables.

As we wrote in comment no. 11, future studies are warranted to explore the effects of shortened postpartum hospitalization on emergency department visits among various sub populations, such as women with elevated BMI, diabetes, mental health disorders and other relevant comorbidities. Such research could provide important insight into the different impact of early discharge policies on maternal health outcomes. We recommended in the Discussion section to perform in a future study a comparison between low and high-risk pregnancies.

14. "No difference" framing overstates the precision of the estimates. The manuscript repeatedly characterises the findings as showing "no difference" between the wartime and control periods. This phrasing edges toward an equivalence claim that the data do not support. The 95% confidence interval for the multivariable odds ratio (0.72–1.41) is compatible with up to a 28% reduction or a 41% increase in the odds of postpartum GER referral. The study has ruled out only large effects; clinically and policy-relevant moderate effects in either direction remain plausible. The post hoc power statement implicitly acknowledges this, yet the language of the discussion is not consistent with it. A more accurate framing throughout, particularly in the abstract, results, and conclusion, would be: "We found no evidence of a difference between the wartime and control periods, with confidence intervals compatible with moderate increases or decreases in postpartum GER risk." Equivalence cannot be claimed without a pre-specified equivalence margin and the corresponding test, which were not performed.
**Reply to comment:** We agree with the comment and added the sentence suggested.

15. Smaller methodological and reporting points. A number of additional points should be addressed:
(a) The methods (line 142) define the postpartum period as 42 days, while Figure 1 plots 45 days. Please reconcile.
(b) Missing data handling is not described. Please specify whether complete-case analysis was used and report the extent of missingness for each variable in the analytical model.
(c) p-values reported as exactly "1" in Table 2 should be reported as ">0.99" to avoid the misleading impression of perfect agreement.
(d) The single-centre, tertiary-hospital design limits generalisability to other Israeli hospitals — particularly those serving evacuated regions — and to other conflict settings. This should be acknowledged more prominently in the limitations.
**Reply to comment:**

a) Legend of Figure 1 was changed, thank you.
b) There was no missing data.
c) Table 2 was erased as suggested in comment no. 12.
d) We thank the editor for their comment. As Chaim Sheba medical Center is a large, tertiary medical center, caring for highly diverse maternal population, including many who were evacuated during the war, we believe that this increases the potential generalizability of our findings, and is one of the study's main strength.

Reviewers' comments:

Reviewer's Responses to Questions

**Comments to the Author**

1. If the authors have adequately addressed your comments raised in a previous round of review and you feel that this manuscript is now acceptable for publication, you may indicate that here to bypass the “Comments to the Author” section, enter your conflict of interest statement in the “Confidential to Editor” section, and submit your "Accept" recommendation.

Reviewer #7: All comments have been addressed

2. Is the manuscript technically sound, and do the data support the conclusions?

The manuscript must describe a technically sound piece of scientific research with data that supports the conclusions. Experiments must have been conducted rigorously, with appropriate controls, replication, and sample sizes. The conclusions must be drawn appropriately based on the data presented.

Reviewer #7: Yes

3. Has the statistical analysis been performed appropriately and rigorously?

Reviewer #7: Yes

4. Have the authors made all data underlying the findings in their manuscript fully available?

The [PLOS Data policy](http://www.plosone.org/static/policies.action#sharing) requires authors to make all data underlying the findings described in their manuscript fully available without restriction, with rare exception (please refer to the Data Availability Statement in the manuscript PDF file). The data should be provided as part of the manuscript or its supporting information, or deposited to a public repository. For example, in addition to summary statistics, the data points behind means, medians and variance measures should be available. If there are restrictions on publicly sharing data—e.g. participant privacy or use of data from a third party—those must be specified.

Reviewer #7: Yes

5. Is the manuscript presented in an intelligible fashion and written in standard English?

PLOS ONE does not copyedit accepted manuscripts, so the language in submitted articles must be clear, correct, and unambiguous. Any typographical or grammatical errors should be corrected at revision, so please note any specific errors here.

Reviewer #7: Yes

6. Review Comments to the Author

Please use the space provided to explain your answers to the questions above. You may also include additional comments for the author, including concerns about dual publication, research ethics, or publication ethics. (Please upload your review as an attachment if it exceeds 20,000 characters)

Reviewer #7: The authors have comprehensively addressed all the comments and concerns raised in my previous review of the manuscript

7. PLOS authors have the option to publish the peer review history of their article ([what does this mean?](https://journals.plos.org/plosone/s/editorial-and-peer-review-process#loc-peer-review-history)). If published, this will include your full peer review and any attached files.


If you choose “no”, your identity will remain anonymous but your review may still be made public.


**Do you want your identity to be public for this peer review?** For information about this choice, including consent withdrawal, please see our [Privacy Policy](https://www.plos.org/privacy-policy).

Reviewer #7: No
