# Supplementary material for: Early hospital discharge following delivery during wartime is not associated with increased rates of postpartum hospital referrals
Source: PLoS One. 2026 Oct 16;21(10):e0335688. doi: 10.1371/journal.pone.0335688 (PMC13674708; doi:10.1371/journal.pone.0335688)
Supplement: Supplementary file 7 [file pone.0335688.s011.docx]

Date: Tuesday, July 7, 2026

To:

Alejandro Torrado Pacheco, PhD

Danish Ahmad, MBBS,MSc,MNAMS,PhD,IP-H(UK),FRCP(Edin),FRCP(Lon)

Academic Editor
PLOS ONE

Re: PONE-D-24-57057 entitled:

Early Hospital Discharge Following Delivery During Wartime is not Associated with Increased Rates of Postpartum Hospital Referrals,

Dear Dr. Alejandro Torrado Pacheco, and Dr. Danish Ahmad,

We would like to thank you, the editors of PLOS ONE Journal for considering our manuscript for PLOS ONE Journal and for your time and efforts to review this manuscript.

We have addressed each comment in the following reply and have revised the manuscript accordingly. We hope that the 7^h^ revised version of the manuscript is considered suitable for publication in PLOS ONE Journal.

An itemized reply to the comments of the Reviewers is provided below.

Yours sincerely,

Prof. Alina Weissmann-Brenner.

**Reviewers' comments:**

Please also address the following typographical/grammatical errors, and review the text for any other errors:
- Line 27: replace "=" with "-"

Response to comment: Thank you, replacement performed as requested.

- Line 32: replace "and 913 delivered..." with "and 913 who delivered"

Response to comment: Thank you, replacement performed as requested.

- Line 49: remove duplicate ethics statement

Response to comment: Thank you, removal performed as requested.

- Line 94: replace "deranged" with more accurate wording (e.g. "impacted")

Response to comment: Thank you, replacement performed as requested.

- Line 122: replace "health" with "healthy"

Response to comment: Thank you, replacement performed as requested.

- Line 316: remove stray period

Response to comment: Thank you, removal performed as requested.

- Line 321: replace "import" with "important"

Response to comment: Thank you, replacement performed as requested.
