# Supplementary material for: Early hospital discharge following delivery during wartime is not associated with increased rates of postpartum hospital referrals
Source: PLoS One. 2026 Oct 16;21(10):e0335688. doi: 10.1371/journal.pone.0335688 (PMC13674708; doi:10.1371/journal.pone.0335688)
Supplement: Supplementary file 8 [file pone.0335688.s012.docx]

Date: Tuesday, August 11, 2026

To:

Alejandro Torrado Pacheco, PhD

Danish Ahmad, MBBS,MSc,MNAMS,PhD,IP-H(UK),FRCP(Edin),FRCP(Lon)

Academic Editor
PLOS ONE

Re: PONE-D-24-57057 entitled:

Early Hospital Discharge Following Delivery During Wartime is not Associated with Increased Rates of Postpartum Hospital Referrals,

Dear Dr. Alejandro Torrado Pacheco, and Dr. Danish Ahmad,

We would like to thank you, the editors of PLOS ONE Journal for considering our manuscript for PLOS ONE Journal and for your time and efforts to review this manuscript.

We have added the contribution of the author Mrs. Agay Nirit. We hope that the 8^h^ revised version of the manuscript is considered suitable for publication in PLOS ONE Journal.

Yours sincerely,

Prof. Alina Weissmann-Brenner.
