# Supplementary material for: Early hospital discharge following delivery during wartime is not associated with increased rates of postpartum hospital referrals
Source: PLoS One. 2026 Oct 16;21(10):e0335688. doi: 10.1371/journal.pone.0335688 (PMC13674708; doi:10.1371/journal.pone.0335688)
Supplement: Peer review history

# Peer review: Decision Letter 0

**DOI:** 10.1371/journal.pone.0335688.r001

**Authors:** Ahmed Maged

29 Apr 2025

Dear Dr. Weissmann-Brenner,

Thank you for submitting your manuscript to PLOS ONE. After careful consideration, we feel that it has merit but does not fully meet PLOS ONE’s publication criteria as it currently stands. Therefore, we invite you to submit a revised version of the manuscript that addresses the points raised during the review process.

==============================

**ACADEMIC EDITOR: Please respond to all reviewers comments**

Please submit your revised manuscript by Jun 13 2025 11:59PM. If you will need more time than this to complete your revisions, please reply to this message or contact the journal office at plosone@plos.org. When you're ready to submit your revision, log on to https://www.editorialmanager.com/pone/ and select the 'Submissions Needing Revision' folder to locate your manuscript file.

- A rebuttal letter that responds to each point raised by the academic editor and reviewer(s). You should upload this letter as a separate file labeled 'Response to Reviewers'.
- A marked-up copy of your manuscript that highlights changes made to the original version. You should upload this as a separate file labeled 'Revised Manuscript with Track Changes'.
- An unmarked version of your revised paper without tracked changes. You should upload this as a separate file labeled 'Manuscript'.

If you would like to make changes to your financial disclosure, please include your updated statement in your cover letter. Guidelines for resubmitting your figure files are available below the reviewer comments at the end of this letter.

If applicable, we recommend that you deposit your laboratory protocols in protocols.io to enhance the reproducibility of your results. Protocols.io assigns your protocol its own identifier (DOI) so that it can be cited independently in the future. For instructions see: https://journals.plos.org/plosone/s/submission-guidelines#loc-laboratory-protocols. Additionally, PLOS ONE offers an option for publishing peer-reviewed Lab Protocol articles, which describe protocols hosted on protocols.io. Read more information on sharing protocols at https://plos.org/protocols?utm_medium=editorial-email&utm_source=authorletters&utm_campaign=protocols.

We look forward to receiving your revised manuscript.

Kind regards,

Ahmed Mohamed Maged, MD

Academic Editor

PLOS ONE

Comments from PLOS Editorial Office: We note that one or more reviewers has recommended that you cite specific previously published works. As always, we recommend that you please review and evaluate the requested works to determine whether they are relevant and should be cited. It is not a requirement to cite these works. We appreciate your attention to this request.

Journal requirements:

When submitting your revision, we need you to address these additional requirements.

1. Please ensure that your manuscript meets PLOS ONE's style requirements, including those for file naming. The PLOS ONE style templates can be found at

https://journals.plos.org/plosone/s/file?id=wjVg/PLOSOne_formatting_sample_main_body.pdf and

https://journals.plos.org/plosone/s/file?id=ba62/PLOSOne_formatting_sample_title_authors_affiliations.pdf.

2. We noticed you have some minor occurrence of overlapping text with the following previous publication(s), which needs to be addressed:

https://doi.org/10.1515/jpm-2020-0337

In your revision ensure you cite all your sources (including your own works), and quote or rephrase any duplicated text outside the methods section. Further consideration is dependent on these concerns being addressed.

3. In the online submission form, you indicated that [The data that support the findings of this study are available on request from the corresponding author. The data are not publicly available due to privacy or ethical restrictions.].

All PLOS journals now require all data underlying the findings described in their manuscript to be freely available to other researchers, either 1. In a public repository, 2. Within the manuscript itself, or 3. Uploaded as supplementary information.

This policy applies to all data except where public deposition would breach compliance with the protocol approved by your research ethics board. If your data cannot be made publicly available for ethical or legal reasons (e.g., public availability would compromise patient privacy), please explain your reasons on resubmission and your exemption request will be escalated for approval.

4. Your ethics statement should only appear in the Methods section of your manuscript. If your ethics statement is written in any section besides the Methods, please move it to the Methods section and delete it from any other section. Please ensure that your ethics statement is included in your manuscript, as the ethics statement entered into the online submission form will not be published alongside your manuscript.

[Note: HTML markup is below. Please do not edit.]

Reviewers' comments:

Reviewer's Responses to Questions

**Comments to the Author**

1. Is the manuscript technically sound, and do the data support the conclusions?

Reviewer #1: Partly

Reviewer #2: Yes

Reviewer #3: Partly

Reviewer #4: Yes

Reviewer #5: Yes

Reviewer #6: Partly

**********

2. Has the statistical analysis been performed appropriately and rigorously?

Reviewer #1: No

Reviewer #2: Yes

Reviewer #3: No

Reviewer #4: Yes

Reviewer #5: Yes

Reviewer #6: No

**********

3. Have the authors made all data underlying the findings in their manuscript fully available?

The PLOS Data policy

Reviewer #1: Yes

Reviewer #2: Yes

Reviewer #3: No

Reviewer #4: No

Reviewer #5: No

Reviewer #6: Yes

**********

4. Is the manuscript presented in an intelligible fashion and written in standard English?

Reviewer #1: Yes

Reviewer #2: Yes

Reviewer #3: Yes

Reviewer #4: Yes

Reviewer #5: Yes

Reviewer #6: Yes

**********

Reviewer #1: This study evaluates the impact of war on postpartum hospitalization and maternal outcomes in a unique setting where an active conflict coexists with a highly developed and advanced medical system. The study highlights operational adaptations in obstetric care, particularly the shortening of postpartum hospitalization, and assesses its implications for maternal health. While this research provides valuable insights into healthcare adjustments in crisis settings, several aspects require further scrutiny, particularly regarding the clinical significance of the reported hospitalization reduction, differentiation between low- and high-risk patients, and shifts in obstetric decision-making.

The study reports a 13.4% reduction in postpartum hospitalization, with a 9.4% decrease after vaginal delivery and a 23% decrease after cesarean section (p < 0.0001). However, in absolute terms, the reduction translates to a median of approximately 5 hours for vaginal births and 20 hours for cesarean sections. Given that postpartum length of stay in many high-resource settings, including Israel, is already longer than international standards, the clinical impact of this reduction remains questionable.

Clinical Relevance of Reduced Hospitalization Duration

The study does not define a threshold for what constitutes “short” hospitalization. Prior research, such as Bernstein et al. (2016) (PMC4783077), defined 24 hours as the threshold for short hospitalization and found that reducing postpartum length of stay to 36 hours did not increase maternal morbidity, provided that appropriate follow-up care was in place. In contrast, the median postpartum hospitalization in this study remained 49.3 hours after vaginal delivery and 68.6 hours after cesarean section, even during wartime.

While the reduction is statistically significant, it is unclear whether it reflects a meaningful clinical adaptation or a minor logistical adjustment that had little impact on patient outcomes. A more substantial decrease in hospitalization duration, such as a shift toward 24-hour postpartum discharge, would have raised concerns about potential gaps in maternal care. However, the observed reduction—while indicative of an operational shift—may not represent a major change in postpartum management, particularly when compared to global trends in early discharge.

Additionally, it remains uncertain whether this reduction was imposed as a hospital-wide mandate or was selectively applied to patients based on medical criteria. If all postpartum women were uniformly discharged earlier, including high-risk patients, this could have serious clinical implications. Conversely, if early discharge was limited to low-risk women with stable postpartum courses, the impact on maternal and neonatal health would likely be minimal. A subgroup analysis distinguishing low- vs. high-risk women would help clarify whether earlier discharge affected outcomes differently across patient populations.

Changes in Cesarean Section Practices and Obstetric Decision-Making

The study also reports a significant reduction in elective cesarean sections during wartime (57% vs. 92% of total cesareans, p < 0.0001). This dramatic shift suggests a major change in obstetric decision-making, but the study does not explore the reasons behind it.

Several factors could explain this trend:

1. Resource Allocation: During wartime, hospital resources may have been reallocated toward emergency and trauma care, limiting the availability of scheduled elective surgeries, including cesareans.

2. Maternal Preferences: Women may have actively chosen to avoid elective cesareans due to concerns about prolonged hospitalization or potential disruptions in postnatal care.

3. Clinical Prioritization: Obstetric teams may have prioritized vaginal delivery over elective cesareans to reduce surgical burden and hospital stay.

While a shift away from elective cesareans might be expected in a crisis setting, the study does not examine whether this change had clinical consequences. Elective cesarean sections are typically performed under controlled conditions, allowing for better preparation and lower emergency risks. If wartime conditions led to an increase in non-elective or emergency cesareans, this could have implications for maternal and neonatal outcomes. The study should assess whether the reduction in elective cesareans led to an increase in labor complications, emergency deliveries, or neonatal distress.

Postpartum Emergency Room Visits and Alternative Care-Seeking Behavior

Despite the reduction in hospitalization duration, the study found no significant increase in postpartum referrals to the gynecological emergency room (GER) (8.7% vs. 8.8%). This is an important finding, as one might expect an increase in postpartum complications if early discharge led to inadequate follow-up care. The lack of a rise in GER visits suggests that women may have sought postpartum care in alternative settings rather than returning to the hospital.

Given Israel’s well-developed outpatient care system, it is plausible that postpartum women preferred to seek care in HMO clinics rather than returning to the hospital, particularly during wartime. The study does not examine whether there was a corresponding increase in postpartum clinic visits, which would support the hypothesis that care-seeking behavior shifted from hospital-based to community-based settings.

To strengthen its conclusions, the study should investigate:

Reviewer #2: A very interesting study on a subject less studied.

Written well and concise. Comprehensible.

I have 2 minor comments. I noticed a few misspelled words such as “holyday” and a few grammatical errors, therefore English editing can further improve the manuscript. Secondly, consider adding to the limitations that the external validity of the study is very limited due to the unique conditions that characterize a certain country, with a relatively small population, where military service and reserve military service are mandatory, leading to a high rate of recruitment among the general population as well as among medical staff. The study design may be irreproducible. Otherwise, a very enjoyable and educative article.

Reviewer #3: The manuscript's validity and conclusions would be strengthened by addressing the following key issues:

1. Matching and Control: To ensure comparability between groups, the authors should employ matching techniques, such as propensity score matching, to balance cases and controls based on relevant covariates.

2. In-depth Statistical Analysis: Consultation with a statistician is recommended to perform a more comprehensive analysis, including multivariate models like logistic regression or Poisson regression, to account for potential confounders and draw robust conclusions.

3. Data Cleaning and Transparency: The methodology should include a detailed description of data cleaning processes, including handling of missing values, variable selection, and reasons for data exclusion.

Specific Suggestions for Improvement

1. Introduction: Enhance the introduction by citing relevant publications on healthcare during wartime, highlighting hospitalization needs, and providing statistics. Clearly articulate the research gap and objectives.

2. Analysis: Explore potential confounders, such as age categories, occupation, residence, parity, comorbidities, and type of deliveries, and adjust for them in the multivariate analysis.

3. Methodology: Provide a transparent description of data cleaning and processing steps. The authors should also provide context of the hospital and the location of the hospital

Conclusion

By addressing these concerns and incorporating the suggested improvements, the manuscript can provide more robust insights into the impact of early hospital discharge during wartime on postpartum hospital referrals. With revisions, the study's validity and contribution to the field can be significantly enhanced.

Reviewer #4: Summary & Contribution:

This paper presents a timely and persuasive data-driven analysis of postpartum outcomes among women discharged early following delivery during wartime. The authors convincingly show that early discharge during emergency conditions—specifically war—did not lead to increased gynecological emergency room (GER) visits or readmissions. This is an important and novel contribution, particularly given the paucity of evidence on obstetric care adaptations during conflict scenarios. The work offers actionable insights for public health policy and emergency preparedness protocols in regions experiencing war or similar large-scale disruptions.

Strengths:

Novelty and Relevance: To my knowledge, this is the first study examining early postpartum discharge outcomes during a wartime context, filling an important gap in obstetric literature.

Robust Dataset: The study uses electronic medical records from a large tertiary hospital, includes a sizable patient population (N=1,714), and compares wartime data with a well-matched control period.

Clear Methodology: The authors used appropriate statistical comparisons and provided well-structured tables that allow easy interpretation of the results.

Policy Implications: The findings are especially useful for informing emergency response protocols in healthcare systems during times of conflict.

Areas for Improvement and Suggestions:

Data Accessibility for Future Research:

While the paper states that data is available on request, I strongly recommend that a de-identified version of the dataset be made available in a public repository (e.g., Dryad or Figshare). This would greatly enhance transparency and facilitate future meta-analyses or cross-country comparisons.

Data Visualization:

The authors could strengthen their argument by adding visual summaries, such as:

A boxplot comparing hospitalization durations by delivery mode (vaginal vs cesarean) across the two time periods.

A bar chart or stacked histogram of GER referral reasons by group (e.g., infections, bleeding).

A time-to-event curve (e.g., Kaplan-Meier) for GER visits or readmissions to assess timing distributions post-discharge.

Clarification of Elective vs Non-Elective Cesarean Patterns:

The sharp drop in elective cesareans (from 92% to 57%) is striking. While this is acknowledged, it would be valuable to include a short discussion on potential implications—e.g., patient autonomy, clinical risk-taking, or resource prioritization in wartime.

Discussion on External Validity:

The paper mentions generalizability due to population diversity. However, a short paragraph elaborating on how these findings might translate to low-resource or conflict-affected regions with weaker health infrastructure would enhance its utility for global health readers.

Call for Further Research:

The authors suggest a nationwide study, which is appropriate. To guide this future work, they could briefly propose potential variables or methods (e.g., linking hospital discharge data with community midwife visits or primary care outcomes) to enhance comprehensiveness.

Minor Edits:

The manuscript occasionally uses passive constructions ("data was retrieved...")—minor revisions for readability and active voice would improve flow.

Figure/table legends could be more descriptive for stand-alone clarity.

Reviewer #5: Early Hospital Discharge Following Delivery During Wartime is not Associated with

Increased Rates of Postpartum Hospital Referrals

The topic is very interesting and addresses an issue that could hardly be addressed except in a state of exception such as war.

Abstract: The abstract needs better contextualization about war and risk locations. It is necessary to consider the bias of patients not returning because readmission may not have occurred also due to access issues and risks inherent to war.

The introduction is clear, but it could provide more data and information from studies on the topic, especially when dealing with states of exception such as floods, wars and the COVID period itself, which could serve as a comparison.

Regarding the methods, it would be interesting if the authors described the indicator related to infection after cesarean section compared between the groups.

The results are clear, but the tables are polluted and should be better adjusted.

The discussion is well-prepared and brings rich elements to discuss a new approach. I miss the text's approach to home strategies in the postpartum assessment period, with the aim of preventing patient readmission.

The references are adequate, but I miss historical references from the past about the text and its contextualization.

Therefore, I consider the article interesting, but it should be adjusted with the recommendations made here to be eligible for publication.

Reviewer #6: The topic studied by the authors is how the frequency of postpartum complications and other medical issues varies upon the shortening of hospitalization, in the context of the aftermath of a traumatic national event. Sadly, this is a worthwhile and relevant topic of study, which may also be applicable to other environments in which postpartum stays have been shortened due to adverse circumstances, such as active warzones.

The study design used by the authors involves comparing a data from a cohort of people who gave birth in the month after the dreadful October 7 attacks on Israel in 2023 with a cohort of people from the same area who gave birth in the same month of the previous year. The authors have treated the two groups are comparable and have not made statistical adjustments such as regression.

A different study design would have been preferable to use. A pre-post study design in which data are compared from those who gave birth in the month before the attacks and the month after, which would likely reduce the effect from other confounders, relative to choosing the corresponding time period in the previous year as the comparator group. A method such as regression discontinuity or interrupted time series could then be used. Use of data from the two month-long periods, separated by a year, should have been accompanied by adjustments for possible differences in the cohort using appropriate statistical regression approaches and/or, a propensity score-based approach such as matching or IPTW, including additional data on appropriate factors that may affect the length of postpartum hospital stay.

Attention should also be given the description of the circumstances and environment of the individuals in the cohort under study. I am uncomfortable with the use of the word “warzone” being applied to the area of Ramat Gan or even the greater Tel Aviv region (it seems to me as being implied) as it is 60-70 km from the nearest part of Gaza, which is unambiguously a warzone. The circumstances seem to me to be those of living in the traumatic aftermath of a terrorist with the reasonable worry of further attack rather than civilians being directly affected by war fighting (c.f. parts of Gaza). I could not find reports that this area was attacked (e.g. by rockets) in the month after October 7, 2023, and it had been, it is highly likely the authors would have (appropriately) mentioned this in the manuscript. Also, each sentence in lines 85-88 (second paragraph) requires a reference, to news articles or government statements if necessary. The use of the word “wartime” is also not without issue – the circumstances necessitating the shorter postpartum stays should be described using more generally applicable language. Accurate, and specific description of the cohort and relevant circumstances is important for any epidemiological study.

**********

what does this mean?). If published, this will include your full peer review and any attached files.

If you choose “no”, your identity will remain anonymous but your review may still be made public.

**Do you want your identity to be public for this peer review?**  For information about this choice, including consent withdrawal, please see our Privacy Policy

Reviewer #1: No

Reviewer #2: No

Reviewer #3: No

Reviewer #4: **Yes:** Taposh Dutta Roy

Reviewer #5: **Yes:** Vicente Sperb Antonello

Reviewer #6: No

**********

[NOTE: If reviewer comments were submitted as an attachment file, they will be attached to this email and accessible via the submission site. Please log into your account, locate the manuscript record, and check for the action link "View Attachments". If this link does not appear, there are no attachment files.]

While revising your submission, please upload your figure files to the Preflight Analysis and Conversion Engine (PACE) digital diagnostic tool, https://pacev2.apexcovantage.com/. PACE helps ensure that figures meet PLOS requirements. To use PACE, you must first register as a user. Registration is free. Then, login and navigate to the UPLOAD tab, where you will find detailed instructions on how to use the tool. If you encounter any issues or have any questions when using PACE, please email PLOS at figures@plos.org. Please note that Supporting Information files do not need this step.

Attachment Submitted filename: early hospital review.docx

# Author comment: Author response to Decision Letter 1

**DOI:** 10.1371/journal.pone.0335688.r002

17 Jul 2025

Date: Wednesday, June 11, 2025

To: Ahmed Mohamed Maged, MD

Academic Editor

PLOS ONE

Re: PONE-D-24-57057 entitled:

Early Hospital Discharge Following Delivery During Wartime is not Associated with Increased Rates of Postpartum Hospital Referrals

PLOS ONE

Dear Dr. Ahmed Mohamed Maged,

We would like to thank you, the editor of PLOS ONE Journal, and the reviewers for considering our manuscript for PLOS ONE Journal and for your time and efforts to review this manuscript. We feel that your insightful comments and suggestions have helped to improve the quality of our manuscript.

We have addressed each comment in the following reply and have revised the manuscript accordingly. We hope that the revised version of the manuscript is considered suitable for publication in PLOS ONE Journal.

An itemized reply to the comments of the Reviewers is provided below.

An Exel file with the data is attached.

Yours sincerely,

Prof. Alina Weissmann-Brenner.

Reviewers’ comment:

Reviewer #1: This study evaluates the impact of war on postpartum hospitalization and maternal outcomes in a unique setting where an active conflict coexists with a highly developed and advanced medical system. The study highlights operational adaptations in obstetric care, particularly the shortening of postpartum hospitalization, and assesses its implications for maternal health. While this research provides valuable insights into healthcare adjustments in crisis settings, several aspects require further scrutiny, particularly regarding the clinical significance of the reported hospitalization reduction, differentiation between low- and high-risk patients, and shifts in obstetric decision-making.

The study reports a 13.4% reduction in postpartum hospitalization, with a 9.4% decrease after vaginal delivery and a 23% decrease after cesarean section (p < 0.0001). However, in absolute terms, the reduction translates to a median of approximately 5 hours for vaginal births and 20 hours for cesarean sections. Given that postpartum length of stay in many high-resource settings, including Israel, is already longer than international standards, the clinical impact of this reduction remains questionable.

Reply to comment: We thank the reviewer for his/her favorable assessment of the manuscript. Indeed, some medical centers do offer postpartum discharge 24 hours following delivery, while others discharge postpartum patients following several days even after vaginal delivery.

Attached please find a google search regarding the hospitalization time in several medical centers. We believe that our study emphasizes the possibility to safely discharge postpartum patients earlier in specific cases such as national crisis.

Clinical Relevance of Reduced Hospitalization Duration

The study does not define a threshold for what constitutes “short” hospitalization. Prior research, such as Bernstein et al. (2016) (PMC4783077), defined 24 hours as the threshold for short hospitalization and found that reducing postpartum length of stay to 36 hours did not increase maternal morbidity, provided that appropriate follow-up care was in place. In contrast, the median postpartum hospitalization in this study remained 49.3 hours after vaginal delivery and 68.6 hours after cesarean section, even during wartime.

While the reduction is statistically significant, it is unclear whether it reflects a meaningful clinical adaptation or a minor logistical adjustment that had little impact on patient outcomes. A more substantial decrease in hospitalization duration, such as a shift toward 24-hour postpartum discharge, would have raised concerns about potential gaps in maternal care. However, the observed reduction—while indicative of an operational shift—may not represent a major change in postpartum management, particularly when compared to global trends in early discharge.

Additionally, it remains uncertain whether this reduction was imposed as a hospital-wide mandate or was selectively applied to patients based on medical criteria. If all postpartum women were uniformly discharged earlier, including high-risk patients, this could have serious clinical implications. Conversely, if early discharge was limited to low-risk women with stable postpartum courses, the impact on maternal and neonatal health would likely be minimal. A subgroup analysis distinguishing low- vs. high-risk women would help clarify whether earlier discharge affected outcomes differently across patient populations.

Reply to comment: We thank the reviewer for this comment. As pointed at the attached photo from google some hospitals discharge postpartum women 24 hour following delivery, while others discharge them following a few days. The present study emphasizes that it is safe to consider earlier postpartum discharge in specific conditions. The early discharge took place after careful medical examination.

We agree with the reviewer that the comparison between low risk patients and high- risk patients is interesting, therefore we intend to perform such a study.

Changes in Cesarean Section Practices and Obstetric Decision-Making

The study also reports a significant reduction in elective cesarean sections during wartime (57% vs. 92% of total cesareans, p < 0.0001). This dramatic shift suggests a major change in obstetric decision-making, but the study does not explore the reasons behind it.

Several factors could explain this trend:

1. Resource Allocation: During wartime, hospital resources may have been reallocated toward emergency and trauma care, limiting the availability of scheduled elective surgeries, including cesareans.

2. Maternal Preferences: Women may have actively chosen to avoid elective cesareans due to concerns about prolonged hospitalization or potential disruptions in postnatal care.

3. Clinical Prioritization: Obstetric teams may have prioritized vaginal delivery over elective cesareans to reduce surgical burden and hospital stay.

While a shift away from elective cesareans might be expected in a crisis setting, the study does not examine whether this change had clinical consequences. Elective cesarean sections are typically performed under controlled conditions, allowing for better preparation and lower emergency risks. If wartime conditions led to an increase in non-elective or emergency cesareans, this could have implications for maternal and neonatal outcomes. The study should assess whether the reduction in elective cesareans led to an increase in labor complications, emergency deliveries, or neonatal distress.

Reply to comment: We thank the reviewer for the comment. As requested, we added a discussion regarding the elective cesarean section. Our study found no significant differences in the rate of meconium, rate of low Apgar scores and the rate of admissions to the Newborn Intensive Care Unit between the wartime and the similar period in the previous year. Therefore, the shift seen in the rate of elective cesarean section had no clinical impact.

Postpartum Emergency Room Visits and Alternative Care-Seeking Behavior

Despite the reduction in hospitalization duration, the study found no significant increase in postpartum referrals to the gynecological emergency room (GER) (8.7% vs. 8.8%). This is an important finding, as one might expect an increase in postpartum complications if early discharge led to inadequate follow-up care. The lack of a rise in GER visits suggests that women may have sought postpartum care in alternative settings rather than returning to the hospital.

Given Israel’s well-developed outpatient care system, it is plausible that postpartum women preferred to seek care in HMO clinics rather than returning to the hospital, particularly during wartime. The study does not examine whether there was a corresponding increase in postpartum clinic visits, which would support the hypothesis that care-seeking behavior shifted from hospital-based to community-based settings.

Reply to comment: We thank the reviewer for this favorable comment.

Reviewer #2: A very interesting study on a subject less studied.

Written well and concise. Comprehensible.

I have 2 minor comments. I noticed a few misspelled words such as “holyday” and a few grammatical errors, therefore English editing can further improve the manuscript. Secondly, consider adding to the limitations that the external validity of the study is very limited due to the unique conditions that characterize a certain country, with a relatively small population, where military service and reserve military service are mandatory, leading to a high rate of recruitment among the general population as well as among medical staff. The study design may be irreproducible. Otherwise, a very enjoyable and educative article.

Reply to comment: We thank the reviewer for his/her favorable comment.

We corrected grammatical errors and added the suggested limitation to the Discussion section.

Reviewer #3: The manuscript's validity and conclusions would be strengthened by addressing the following key issues:

1. Matching and Control: To ensure comparability between groups, the authors should employ matching techniques, such as propensity score matching, to balance cases and controls based on relevant covariates.

2. In-depth Statistical Analysis: Consultation with a statistician is recommended to perform a more comprehensive analysis, including multivariate models like logistic regression or Poisson regression, to account for potential confounders and draw robust conclusions.

3. Data Cleaning and Transparency: The methodology should include a detailed description of data cleaning processes, including handling of missing values, variable selection, and reasons for data exclusion.

Reply to comment: We thank the reviewer for the comment. A multivariable

logistic regression model was performed as requested. Data was added to the Method section and to the Result section.

Specific Suggestions for Improvement

1. Introduction: Enhance the introduction by citing relevant publications on healthcare during wartime, highlighting hospitalization needs, and providing statistics. Clearly articulate the research gap and objectives.

Reply to comment: We added references as requested, and made clarification of the research gap and objectives as requested.

2. Analysis: Explore potential confounders, such as age categories, occupation, residence, parity, comorbidities, and type of deliveries, and adjust for them in the multivariate analysis.

Reply to comment: We thank the reviewer for the comment. A multivariable logistic regression model was performed as requested. Data was added to the Method section and to the Result section. We do not have information about the occupation of our patients. We agree that knowledge of the place of residence is important. However, because of the missile attacks on Israel about 64,000 Israelis were evacuated from their homes in the southern parts of Israel, and about 61,000 Israelis were evacuated from their homes in the northern parts of Israel. They all moved to temporary residence during the war. In the computerized data base we have information about the permanent residence. Because of the changes during the war this information may not be correct, therefore we did not include it in our analysis.

3. Methodology: The authors should also provide context of the hospital and the location of the hospital

Reply to comment: The Chaim Sheba Medical Center is a tertiary hospital in the center of Israel, that treated many wounded soldiers and civilians. Many of the hospital workers and/or their spouses were recruited to the army during the war. The hospital made many changes and adaptations in personnel and in equipment in all departments, including transfer of staff between departments and reinforcement of the departments according to the specific needs.

Conclusion

By addressing these concerns and incorporating the suggested improvements, the manuscript can provide more robust insights into the impact of early hospital discharge during wartime on postpartum hospital referrals. With revisions, the study's validity and contribution to the field can be significantly enhanced.

Reply to comment: We thank the reviewer for his/her comments.

Reviewer #4: Summary & Contribution:

This paper presents a timely and persuasive data-driven analysis of postpartum outcomes among women discharged early following delivery during wartime. The authors convincingly show that early discharge during emergency conditions—specifically war—did not lead to increased gynecological emergency room (GER) visits or readmissions. This is an important and novel contribution, particularly given the paucity of evidence on obstetric care adaptations during conflict scenarios. The work offers actionable insights for public health policy and emergency preparedness protocols in regions experiencing war or similar large-scale disruptions.

Strengths:

Novelty and Relevance: To my knowledge, this is the first study examining early postpartum discharge outcomes during a wartime context, filling an important gap in obstetric literature.

Robust Dataset: The study uses electronic medical records from a large tertiary hospital, includes a sizable patient population (N=1,714), and compares wartime data with a well-matched control period.

Clear Methodology: The authors used appropriate statistical comparisons and provided well-structured tables that allow easy interpretation of the results.

Policy Implications: The findings are especially useful for informing emergency response protocols in healthcare systems during times of conflict.

Areas for Improvement and Suggestions:

Data Accessibility for Future Research:

While the paper states that data is available on request, I strongly recommend that a de-identified version of the dataset be made available in a public repository (e.g., Dryad or Figshare). This would greatly enhance transparency and facilitate future meta-analyses or cross-country comparisons.

Reply to comment: We thank the reviewer for his/her favorable comments.

Data Visualization:

The authors could strengthen their argument by adding visual summaries, such as:

A boxplot comparing hospitalization durations by delivery mode (vaginal vs cesarean) across the two time periods.

A bar chart or stacked histogram of GER referral reasons by group (e.g., infections, bleeding).

A time-to-event curve (e.g., Kaplan-Meier) for GER visits or readmissions to assess timing distributions post-discharge.

Reply to comment: We thank the reviewer for the comment. A time to event curve (Kaplan Meyer) was added (Figure 1) as requested. Data was added to the Method section and to the Result section.

Clarification of Elective vs Non-Elective Cesarean Patterns:

The sharp drop in elective cesareans (from 92% to 57%) is striking. While this is acknowledged, it would be valuable to include a short discussion on potential implications—e.g., patient autonomy, clinical risk-taking, or resource prioritization in wartime.

Reply to comment: We thank the reviewer for his/her favorable comments. We added a discussion regarding the elective cesarean section as requested.

Discussion on External Validity:

The paper mentions generalizability due to population diversity. However, a short paragraph elaborating on how these findings might translate to low-resource or conflict-affected regions with weaker health infrastructure would enhance its utility for global health readers.

Reply to comment: We thank the reviewer for his/her comment. We added a paragraph in the Discussion section as requested.

Call for Further Research:

The authors suggest a nationwide study, which is appropriate. To guide this future work, they could briefly propose potential variables or methods (e.g., linking hospital discharge data with community midwife visits or primary care outcomes) to enhance comprehensiveness.

Reply to comment: We added data to the Method section with information about the Chaim Sheba Medical Center. We added limitation why the study is not reproducible in other countries to the Discussion section

Minor Edits:

The manuscript occasionally uses passive constructions ("data was retrieved...")—minor revisions for readability and active voice would improve flow.

Figure/table legends could be more descriptive for stand-alone clarity.

Reply to comment: We made grammar changes.

Reviewer #5: Early Hospital Discharge Following Delivery During Wartime is not Associated with Increased Rates of Postpartum Hospital Referrals

The topic is very interesting and addresses an issue that could hardly be addressed except in a state of exception such as war.

Reply to comment: We thank the reviewer for his/her favorable review.

Abstract: The abstract needs better contextualization about war and risk locations. It is necessary to consider the bias of patients not returning because readmission may not have occurred also due to access issues and risks inherent to war.

Reply to comment: We added information regarding changes and adaptations in hospitals during war, with the limitation of the 250 words allowed buy the PLOS ONE journal.

The introduction is clear, but it could provide more data and information from studies on the topic, especially when dealing with states of exception such as floods, wars and the COVID period itself, which could serve as a comparison.

Reply to comment: We added more references to the Introduction section. We added information about the lack of knowledge regarding shortening of postpartum hospitalization and its influence on postpartum visits to the gynecological emergency room during war.

Regarding the methods, it would be interesting if the authors described the indicator related to infection after cesarean section compared between the groups.

Reply to comment: We added definition of infection to the Method section.

The results are clear, but the tables are polluted and should be better adjusted.

The discussion is well-prepared and brings rich elements to discuss a new approach. I miss the text's approach to home strategies in the postpartum assessment period, with the aim of preventing patient readmission.

Reply to comment: We added a take home message to the Conclusion section as suggested.

The references are adequate, but I miss historical references from the past about the text and its contextualization.

Reply to comment: We added additional references as suggested.

Therefore, I consider the article interesting, but it should be adjusted with the recommendations made here to be eligible for publication.

Reply to comment: We thank the reviewer for his/her favorable review.

Reviewer #6: The topic studied by the authors is how the frequency of postpartum complications and other medical issues varies upon the shortening of hospitalization, in the context of the aftermath of a traumatic national event. Sadly, this is a worthwhile and relevant topic of study, which may also be applicable to other environments in which postpartum stays have been shortened due to adverse circumstances, such as active warzones.

The study design used by the authors involves comparing a data from a cohort of people who gave birth in the month after the dreadful October 7 attacks on Israel in 2023 with a cohort of people from the same area who gave birth in the same month of the previous year. The authors have treated the two groups are comparable and have not made statistical adjustments such as regression.

A different study design would have been preferable to use. A pre-post study design in which data are compared from those who gave birth in the month before the attacks and the month after, which would likely reduce the effect from other confounders, relative to choosing the corresponding time period in the previous year as the comparator group.

Reply to comment: We thank the reviewer for his/her favorable review. We chose to compare data to a similar period in the previous year due to the Jewish holiday that took place during the war. October 7th 2023 was a Jewish holiday of SIMCHAT TORA. During holidays outpatient clinics are closed, and this may affect the referrals to the hospital. This is the reason we chose to make comparisons to a similar period from SIMCHAT TORA 2022, and not to the previous months, that included other Jewish holydays.

A method such as regression discontinuity or interrupted time series could then be used. Use of data from the two month-long periods, separated by a year, should have been accompanied by adjustments for possible differences in the cohort using appropriate statistical regression approaches and/or, a propensity score-based approach such as matching or IPTW, including additional data on appropriate factors that may affect the length of postpartum hospital stay.

Attention should also be given the description of the circumstances and environment of the individuals in the cohort under study.

Reply to comment: We thank the reviewer for the comment. A multivariable logistic regression model was performed as requested. Data was added to the Method section and to the Result section.

I am uncomfortable with the use of the word “warzone” being applied to the area of Ramat Gan or even the greater Tel Aviv region (it seems to me as being implied) as it is 60-70 km from the nearest part of Gaza, which is unambiguously a warzone. The circumstances seem to me to be those of living in the traumatic aftermath of a terrorist with the reasonable worry of further attack rather than civilians being directly affected by war fighting (c.f. parts of Gaza). I could not find reports that this area was attacked (e.g. by rockets) in the month after October 7, 2023, and it had been, it is highly likely the authors would have (appropriately) mentioned this in the manuscript.

Reply to comment: During the study period of October 7th 2023 and November 5th 2023 there were 44 clusters of missile attacks on the Tel Aviv area, making the Tel Aviv area a true warzone. This information was taken from the Home Front Command internet portal. We added this information to the Method section and the References section.

Also, each sentence in lines 85-88 (second paragraph) requires a reference, to news articles or government statements if necessary. The use of the word “wartime” is also not without issue – the circumstances necessitating the shorter postpartum stays should be described using more generally applicable language. Accurate, and specific description of the cohort and relevant circumstances is important for any epidemiological study.

Reply to comment: We thank the reviewer for this important suggestion. We added references 13-18.

We hope that the revised manuscript is accepted by PLOS ONE Journal. We will be happy to answer any further questions.

Sincerely,

Prof. Alina Weissmann-Brenner

Department of Obstetrics and Gynecology, The Chaim Sheba Medical Center, Tel

HaShomer

Attachment Submitted filename: responce to reviewers.docx

# Peer review: Decision Letter 1

**DOI:** 10.1371/journal.pone.0335688.r003

**Authors:** Ahmed Maged

13 Aug 2025

Dear Dr. Weissmann-Brenner,

Thank you for submitting your manuscript to PLOS ONE. After careful consideration, we feel that it has merit but does not fully meet PLOS ONE’s publication criteria as it currently stands. Therefore, we invite you to submit a revised version of the manuscript that addresses the points raised during the review process.

==============================

**ACADEMIC EDITOR: Please respond to all reviewers comments**

Please submit your revised manuscript by Sep 27 2025 11:59PM. If you will need more time than this to complete your revisions, please reply to this message or contact the journal office at plosone@plos.org. When you're ready to submit your revision, log on to https://www.editorialmanager.com/pone/ and select the 'Submissions Needing Revision' folder to locate your manuscript file.

- A rebuttal letter that responds to each point raised by the academic editor and reviewer(s). You should upload this letter as a separate file labeled 'Response to Reviewers'.
- A marked-up copy of your manuscript that highlights changes made to the original version. You should upload this as a separate file labeled 'Revised Manuscript with Track Changes'.
- An unmarked version of your revised paper without tracked changes. You should upload this as a separate file labeled 'Manuscript'.

If you would like to make changes to your financial disclosure, please include your updated statement in your cover letter. Guidelines for resubmitting your figure files are available below the reviewer comments at the end of this letter.

If applicable, we recommend that you deposit your laboratory protocols in protocols.io to enhance the reproducibility of your results. Protocols.io assigns your protocol its own identifier (DOI) so that it can be cited independently in the future. For instructions see: https://journals.plos.org/plosone/s/submission-guidelines#loc-laboratory-protocols. Additionally, PLOS ONE offers an option for publishing peer-reviewed Lab Protocol articles, which describe protocols hosted on protocols.io. Read more information on sharing protocols at https://plos.org/protocols?utm_medium=editorial-email&utm_source=authorletters&utm_campaign=protocols.

We look forward to receiving your revised manuscript.

Kind regards,

Ahmed Mohamed Maged, MD

Academic Editor

PLOS ONE

Journal Requirements:

If the reviewer comments include a recommendation to cite specific previously published works, please review and evaluate these publications to determine whether they are relevant and should be cited. There is no requirement to cite these works unless the editor has indicated otherwise.

[Note: HTML markup is below. Please do not edit.]

Reviewers' comments:

Reviewer's Responses to Questions

**Comments to the Author**

Reviewer #1: (No Response)

Reviewer #5: All comments have been addressed

**********

2. Is the manuscript technically sound, and do the data support the conclusions?

Reviewer #1: No

Reviewer #5: Yes

**********

3. Has the statistical analysis been performed appropriately and rigorously?

Reviewer #1: No

Reviewer #5: Yes

**********

4. Have the authors made all data underlying the findings in their manuscript fully available?

The PLOS Data policy

Reviewer #1: No

Reviewer #5: Yes

**********

5. Is the manuscript presented in an intelligible fashion and written in standard English?

Reviewer #1: Yes

Reviewer #5: Yes

**********

Reviewer #1: This study addresses an important and unique question; however, several methodological and interpretive issues limit the robustness of the conclusions, and unfortunately, my previous comments on these matters were not fully addressed.

In response to another reviewer, the authors mention the evacuation of approximately 600,000 Israelis to central regions during the study period. This has likely altered the obstetric population at the study hospital and should be mentioned by authors, especially in the context of the marked reduction in cesarean section rate that is unlikely to be explained by changes in departmental protocols (breach, suspected macrosomia, TOLAC) and more plausibly reflects a higher proportion of low-risk pregnancies. The significant differences in the proportion of multiple gestations between periods support this interpretation. Such a shift in case mix represents a potential confounder that requires explicit discussion.

The observed reduction in hospitalization duration was statistically significant but clinically modest - a median of approximately 5 hours for vaginal deliveries and about 20 hours for cesareans - and the median stay remained well above the 24–36 hours commonly used internationally as an early-discharge benchmark (as noted in the authors’ own Google search). Missing from the manuscript is a clear description of the department’s discharge policy and targets during the wartime period - was the intended target 24 hours, 48 hours, or another standard? Clarification is essential, as length of stay is the key determinant in this analysis, and postpartum readmission is the primary outcome. Furthermore, it remains unclear whether this reduction reflects a change in population characteristics, as noted in point (1), or a policy change.

Table 1 contains several numerical inconsistencies: for example, the number of cesareans in the “Mode of delivery” line (211 vs. 284) does not match the sum of the “Cesarean section” breakdown (223 vs. 300). This discrepancy should be clarified, and if it reflects data handling issues, the analysis should be revised. In addition, the manuscript should clearly define the distinction between “non-elective” and “urgent” cesarean deliveries, the definition of meconium, etc.

4. Furthermore, the section on postpartum emergency room visits should be interpreted with caution. The absence of an increase in GER visits may reflect care-seeking in alternative settings, particularly given the large number of internally displaced individuals who may not have returned to the same hospital where they gave birth. In Israel, according to several publications during COVID, HMO and hospital records are interconnected; therefore, a more rigorous effort should be made to assess postpartum morbidity following early discharge through comprehensive system-wide data capture. Reliance solely on GER visits is inadequate, as under missile attacks - as described by the authors-patients would understandably be less inclined to return to hospital-based emergency services.

Reviewer #5: Dear editors and authores,

All replies to the reviewers' comments were received. However, there are still two outstanding issues that need to be adjusted:

1) The definition of infection should follow a scientific reference: "Infections were defined clinically and laboratory in patients with fever, chills, erythema in scar, purulent secretions, leukocytosis, etc." I suggest the CDC reference (https://www.cdc.gov/nhsn/pdfs/pscmanual/17pscnosinfdef_current.pdf), for example, or similar with citation in the text.

2) The references have been updated, but they do not comply with the Plos One guideline. I suggest adjusting them. For example, information about when they were accessed and other information, especially from websites, is missing.

The remaining questions have all been addressed and I believe that after these adjustments, the article can be accepted for publication.

**********

what does this mean?). If published, this will include your full peer review and any attached files.

If you choose “no”, your identity will remain anonymous but your review may still be made public.

**Do you want your identity to be public for this peer review?**  For information about this choice, including consent withdrawal, please see our Privacy Policy

Reviewer #1: No

Reviewer #5: **Yes:** Vicente Sperb Antonello

**********

[NOTE: If reviewer comments were submitted as an attachment file, they will be attached to this email and accessible via the submission site. Please log into your account, locate the manuscript record, and check for the action link "View Attachments". If this link does not appear, there are no attachment files.]

While revising your submission, please upload your figure files to the Preflight Analysis and Conversion Engine (PACE) digital diagnostic tool, https://pacev2.apexcovantage.com/. PACE helps ensure that figures meet PLOS requirements. To use PACE, you must first register as a user. Registration is free. Then, login and navigate to the UPLOAD tab, where you will find detailed instructions on how to use the tool. If you encounter any issues or have any questions when using PACE, please email PLOS at figures@plos.org. Please note that Supporting Information files do not need this step.

# Author comment: Author response to Decision Letter 2

**DOI:** 10.1371/journal.pone.0335688.r004

18 Aug 2025

Reviewers' comments:

Reviewer's Responses to Questions

Comments to the Author

1. If the authors have adequately addressed your comments raised in a previous round of review and you feel that this manuscript is now acceptable for publication, you may indicate that here to bypass the “Comments to the Author” section, enter your conflict of interest statement in the “Confidential to Editor” section, and submit your "Accept" recommendation.

Reviewer #1: (No Response)

Reviewer #5: All comments have been addressed

Reply to comment: We thank the reviewers.

________________________________________

2. Is the manuscript technically sound, and do the data support the conclusions?

The manuscript must describe a technically sound piece of scientific research with data that supports the conclusions. Experiments must have been conducted rigorously, with appropriate controls, replication, and sample sizes. The conclusions must be drawn appropriately based on the data presented.

Reviewer #1: No

Reviewer #5: Yes

Reply to comment: We thank the reviewers. The study described deliveries during the first month of war, compared to a similar period in the previous year.

________________________________________

3. Has the statistical analysis been performed appropriately and rigorously?

Reviewer #1: No

Reviewer #5: Yes

Reply to comment: We thank the reviewers. Comparison was made between deliveries and admission to the hospital in the postpartum period during the first month of war and a similar period in the previous year.

Statistical analysis in described in the method section and included:

Mean and standard deviation (relating to normally distributed continuous variables), median and IQR (relating to non-normally distributed continuous variables) of the aforementioned patients’ characteristics (delivery characteristics and length-of-stay in hospital following delivery) were computed for each period.

Categorical variables were compared by Chi-square tests, and continuous variables were compared via Wilcoxon non-parametric tests or via t-tests.

In order to compare post-partum visits to the GER in the two periods while controlling for potential confounders, a multivariable logistic regression model was fitted to the data, with age at delivery, gestational week (below or above 37), fetus count (one or more), number of previous births (1,2,3,4, 5+). Delivery characteristics and hospital length-of-stay are mediators so they were not included in the model.

In each cohort, a Kaplan-Meier curve for the time until a post-partum referral to the GER was fitted, and the two curves were compared via a log-rank test.

Statistical significance was determined whenever the p value was < 0.05. All analytics were done using the SPSS software (Microsoft inc.).

________________________________________

4. Have the authors made all data underlying the findings in their manuscript fully available?

The PLOS Data policy requires authors to make all data underlying the findings described in their manuscript fully available without restriction, with rare exception (please refer to the Data Availability Statement in the manuscript PDF file). The data should be provided as part of the manuscript or its supporting information, or deposited to a public repository. For example, in addition to summary statistics, the data points behind means, medians and variance measures should be available. If there are restrictions on publicly sharing data—e.g. participant privacy or use of data from a third party—those must be specified.

Reviewer #1: No

Reviewer #5: Yes

Reply to comment: An EXEL file with all the data of the study (without identifying data) was uploaded as supporting information.

________________________________________

5. Is the manuscript presented in an intelligible fashion and written in standard English?

PLOS ONE does not copyedit accepted manuscripts, so the language in submitted articles must be clear, correct, and unambiguous. Any typographical or grammatical errors should be corrected at revision, so please note any specific errors here.

Reviewer #1: Yes

Reviewer #5: Yes

Reply to comment: We thank the reviewers.

________________________________________

6. Review Comments to the Author

Please use the space provided to explain your answers to the questions above. You may also include additional comments for the author, including concerns about dual publication, research ethics, or publication ethics. (Please upload your review as an attachment if it exceeds 20,000 characters)

Reviewer #1: This study addresses an important and unique question; however, several methodological and interpretive issues limit the robustness of the conclusions, and unfortunately, my previous comments on these matters were not fully addressed.

In response to another reviewer, the authors mention the evacuation of approximately 600,000 Israelis to central regions during the study period. This has likely altered the obstetric population at the study hospital and should be mentioned by authors, especially in the context of the marked reduction in cesarean section rate that is unlikely to be explained by changes in departmental protocols (breach, suspected macrosomia, TOLAC) and more plausibly reflects a higher proportion of low-risk pregnancies. The significant differences in the proportion of multiple gestations between periods support this interpretation. Such a shift in case mix represents a potential confounder that requires explicit discussion.

Reply to comment: We thank the reviewer for his/her comment.

Following the attacks on Israel from Gaza and from Lebanon about 143,000 civilians were evacuated from their homes in the north and south of Israel along the borders with Lebanon and Gaza [ref.19 was added to the references]. About 60,000 were evacuated to city of Eilat, the most south city in Israel (near Egypt and Jordan), while others were evacuated to other parts of Israel not close to the borders. The evacuated population included children, elderly people, men and non-pregnant women. There is no statistical data in the Israeli national records regarding the number of pregnant patients evacuated during the war. Since the Tel Aviv area was attacked by 44 clusters of missiles only in the first month of the war, we can assume that many pregnant women preferred to be evacuated to relatively more quiet regions of the country, and delivered in nearby hospitals. Therefore, there were no significant changes in the numbers of deliveries between the study group and the control. Furthermore, low risk pregnant women from the Tel Aviv area might have preferred to move to other quieter places and deliver in hospitals that were less buzzy (as written, Tel HaShomer hospital is a tertiary hospital that received many wounded soldiers and civilians during the war). Because Tel HaShomer is a tertiary hospital experienced and specialized in high risk pregnancies and multiple pregnancies , with an excellent neonatal intensive care unit, patients with multiple pregnancies deliberately come to deliver there.

The data was added to the manuscript.

------------------------------------------------------------------------------------

The observed reduction in hospitalization duration was statistically significant but clinically modest - a median of approximately 5 hours for vaginal deliveries and about 20 hours for cesareans - and the median stay remained well above the 24–36 hours commonly used internationally as an early-discharge benchmark (as noted in the authors’ own Google search). Missing from the manuscript is a clear description of the department’s discharge policy and targets during the wartime period - was the intended target 24 hours, 48 hours, or another standard? Clarification is essential, as length of stay is the key determinant in this analysis, and postpartum readmission is the primary outcome. Furthermore, it remains unclear whether this reduction reflects a change in population characteristics, as noted in point (1), or a policy change.

Reply to comment: We thank the reviewer for his/her comment.

Following delivery, low-risk patients were offered to be discharged earlier from the hospital after careful examination of them and their newborns, after 24 hours for vaginal deliveries and after 48 hours for cesarean sections.

The information was added to the manuscript.

Regarding the shift in the population, as written in the previous answer, most people evacuated were not pregnant, many of them were evacuated to other less bombed parts of Israel.

-----------------------------------------------------------------------------------

Table 1 contains several numerical inconsistencies: for example, the number of cesareans in the “Mode of delivery” line (211 vs. 284) does not match the sum of the “Cesarean section” breakdown (223 vs. 300). This discrepancy should be clarified, and if it reflects data handling issues, the analysis should be revised. In addition, the manuscript should clearly define the distinction between “non-elective” and “urgent” cesarean deliveries, the definition of meconium, etc.

Reply to comment: The number of cesarean sections in Table 1 was corrected, thank you.

Definition of non-elective cesarean section, urgent cesarean section and meconium were made as requested.

---------------------------------------------------------------------------------------

4. Furthermore, the section on postpartum emergency room visits should be interpreted with caution. The absence of an increase in GER visits may reflect care-seeking in alternative settings, particularly given the large number of internally displaced individuals who may not have returned to the same hospital where they gave birth. In Israel, according to several publications during COVID, HMO and hospital records are interconnected; therefore, a more rigorous effort should be made to assess postpartum morbidity following early discharge through comprehensive system-wide data capture. Reliance solely on GER visits is inadequate, as under missile attacks - as described by the authors-patients would understandably be less inclined to return to hospital-based emergency services.

Reply to comment: We thank the reviewer for the comment. Discussion of the subject was added to the Discussion section.

====================================================

Reviewer #5: Dear editors and authores,

All replies to the reviewers' comments were received. However, there are still two outstanding issues that need to be adjusted:

1) The definition of infection should follow a scientific reference: "Infections were defined clinically and laboratory in patients with fever, chills, erythema in scar, purulent secretions, leukocytosis, etc." I suggest the CDC reference (https://www.cdc.gov/nhsn/pdfs/pscmanual/17pscnosinfdef_current.pdf), for example, or similar with citation in the text.

Reply to comment: We thank the reviewer for his/her comment.

The reference was added as requested.

2) The references have been updated, but they do not comply with the Plos One guideline. I suggest adjusting them. For example, information about when they were accessed and other information, especially from websites, is missing.

Reply to comment: We thank the reviewer for his/her comment.

The references were adjusted as requested.

The remaining questions have all been addressed and I believe that after these adjustments, the article can be accepted for publication.

________________________________________

7. PLOS authors have the option to publish the peer review history of their article (what does this mean?). If published, this will include your full peer review and any attached files.

If you choose “no”, your identity will remain anonymous but your review may still be made public.

Do you want your identity to be public for this peer review? For information about this choice, including consent withdrawal, please see our Privacy Policy.

Reviewer #1: No

Reviewer #5: Yes: Vicente Sperb Antonello

Reply to comment: We thank the reviewers.

# Peer review: Decision Letter 2

**DOI:** 10.1371/journal.pone.0335688.r005

**Authors:** Ahmed Maged

26 Aug 2025

Dear Dr. Weissmann-Brenner,

Thank you for submitting your manuscript to PLOS ONE. After careful consideration, we feel that it has merit but does not fully meet PLOS ONE’s publication criteria as it currently stands. Therefore, we invite you to submit a revised version of the manuscript that addresses the points raised during the review process.

==============================

**ACADEMIC EDITOR: Please respond to all reviewer comments**

Please submit your revised manuscript by Oct 10 2025 11:59PM. If you will need more time than this to complete your revisions, please reply to this message or contact the journal office at plosone@plos.org. When you're ready to submit your revision, log on to https://www.editorialmanager.com/pone/ and select the 'Submissions Needing Revision' folder to locate your manuscript file.

- A rebuttal letter that responds to each point raised by the academic editor and reviewer(s). You should upload this letter as a separate file labeled 'Response to Reviewers'.
- A marked-up copy of your manuscript that highlights changes made to the original version. You should upload this as a separate file labeled 'Revised Manuscript with Track Changes'.
- An unmarked version of your revised paper without tracked changes. You should upload this as a separate file labeled 'Manuscript'.

If you would like to make changes to your financial disclosure, please include your updated statement in your cover letter. Guidelines for resubmitting your figure files are available below the reviewer comments at the end of this letter.

If applicable, we recommend that you deposit your laboratory protocols in protocols.io to enhance the reproducibility of your results. Protocols.io assigns your protocol its own identifier (DOI) so that it can be cited independently in the future. For instructions see: https://journals.plos.org/plosone/s/submission-guidelines#loc-laboratory-protocols. Additionally, PLOS ONE offers an option for publishing peer-reviewed Lab Protocol articles, which describe protocols hosted on protocols.io. Read more information on sharing protocols at https://plos.org/protocols?utm_medium=editorial-email&utm_source=authorletters&utm_campaign=protocols.

We look forward to receiving your revised manuscript.

Kind regards,

Ahmed Mohamed Maged, MD

Academic Editor

PLOS ONE

Journal Requirements:

If the reviewer comments include a recommendation to cite specific previously published works, please review and evaluate these publications to determine whether they are relevant and should be cited. There is no requirement to cite these works unless the editor has indicated otherwise.

[Note: HTML markup is below. Please do not edit.]

Reviewers' comments:

Reviewer's Responses to Questions

**Comments to the Author**

Reviewer #1: (No Response)

Reviewer #5: All comments have been addressed

**********

2. Is the manuscript technically sound, and do the data support the conclusions?

Reviewer #1: No

Reviewer #5: Yes

**********

3. Has the statistical analysis been performed appropriately and rigorously?

Reviewer #1: No

Reviewer #5: Yes

**********

4. Have the authors made all data underlying the findings in their manuscript fully available?

The PLOS Data policy

Reviewer #1: Yes

Reviewer #5: Yes

**********

5. Is the manuscript presented in an intelligible fashion and written in standard English?

Reviewer #1: Yes

Reviewer #5: Yes

**********

Reviewer #1: Unfortunately, the authors did not address my concern regarding internal inconsistencies in Table 1. They have corrected the sum of cesareans however did not reviewed the whole table.

In several places, the absolute numbers and percentages are mismatched when back-calculated from. This suggests that either the denominators used for percentages varied or there were data omissions not clarified in the manuscript.

These inconsistencies, collectively reduce confidence in the accuracy of the data presentation. Clarification from the authors is necessary to ensure transparency and reproducibility.

For example:

• Study group (wartime,reported N = 801)

• Normal vaginal delivery: 475

• Vacuum extraction: 52

• Forceps delivery: 4

• Cesarean section: 223

• Subtotal = 754

• Missing cases: 47 deliveries unaccounted for

• Control group (N = 913)

• Normal vaginal delivery: 576

• Vacuum extraction: 42

• Forceps delivery: 2

• Cesarean section: 300

• Subtotal = 920

• Exceeds N by 7 cases

This affects all calculations and statistical analysis:

For example, the percentage of normal vaginal delivery should be approximately 62.99% rather than the reported 59%. And similar inconveniences are present throughout the table.

A comprehensive re-analysis of the data presented in Table 1 is strongly advised to ensure the reliability, accuracy, and reproducibility of the reported findings.

The current version contains substantive internal inconsistencies, most notably in the mode of delivery distributions, where subgroup counts do not correspond with the stated sample sizes, resulting in inaccurate percentages. Additionally, the hospitalization duration data require clarification. The overall interquartile ranges do not align coherently with those of the subgroups, raising questions about the analytic methodology and whether operative vaginal deliveries were categorized consistently. Such discrepancies diminish the interpretability and reliability of the reported findings.

Minor Comments:

•The manuscript notes that parturients were offered discharge after 24 hours; however, it remains unclear whether this reflected a formal early-discharge policy or simply a theoretical option. The reported medians and IQRs approximate 48 hours, which aligns more closely with the standard recommended discharge interval at this center. Clarification on this point is warranted.

•In Table 1, the label “after cesarean” appears duplicated in the IQR row and should be corrected.

•In the legends, the term should be revised to “breech.”

Reviewer #5: All replies to the reviewers' comments were received. However, there one last issue that need to be adjusted.

1) Please adjust the references regarding the Plos One guideline.

The remaining questions have all been addressed and I believe that after these

adjustments, the article can be accepted for publication.

**********

what does this mean?). If published, this will include your full peer review and any attached files.

If you choose “no”, your identity will remain anonymous but your review may still be made public.

**Do you want your identity to be public for this peer review?**  For information about this choice, including consent withdrawal, please see our Privacy Policy

Reviewer #1: No

Reviewer #5: **Yes:** Vicente Sperb Antonello

**********

[NOTE: If reviewer comments were submitted as an attachment file, they will be attached to this email and accessible via the submission site. Please log into your account, locate the manuscript record, and check for the action link "View Attachments". If this link does not appear, there are no attachment files.]

While revising your submission, please upload your figure files to the Preflight Analysis and Conversion Engine (PACE) digital diagnostic tool, https://pacev2.apexcovantage.com/. PACE helps ensure that figures meet PLOS requirements. To use PACE, you must first register as a user. Registration is free. Then, login and navigate to the UPLOAD tab, where you will find detailed instructions on how to use the tool. If you encounter any issues or have any questions when using PACE, please email PLOS at figures@plos.org. Please note that Supporting Information files do not need this step.

# Author comment: Author response to Decision Letter 3

**DOI:** 10.1371/journal.pone.0335688.r006

26 Sep 2025

Date: Wednesday, September 14th, 2025

To: Ahmed Mohamed Maged, MD

Academic Editor

PLOS ONE

Re: PONE-D-24-57057 entitled:

Early Hospital Discharge Following Delivery During Wartime is not Associated with Increased Rates of Postpartum Hospital Referrals.

Dear Dr. Ahmed Mohamed Maged,

We would like to thank you, the editor of PLOS ONE Journal, and the reviewers again for considering our manuscript for publication in the PLOS ONE Journal and for your time and efforts to review this manuscript.

We have addressed each comment in the following reply and have revised the manuscript accordingly. We hope that the revised version of the manuscript is considered suitable for publication in PLOS ONE Journal.

An itemized reply to the comments of the Reviewers is provided below.

Yours sincerely,

Prof. Alina Weissmann-Brenner.

Reviewers' comments:

Reviewer's Responses to Questions

Comments to the Author

1. If the authors have adequately addressed your comments raised in a previous round of review and you feel that this manuscript is now acceptable for publication, you may indicate that here to bypass the “Comments to the Author” section, enter your conflict of interest statement in the “Confidential to Editor” section, and submit your "Accept" recommendation.

Reviewer #1: (No Response)

Reviewer #5: All comments have been addressed

Reply to comment: We thank the reviewer.

________________________________________

2. Is the manuscript technically sound, and do the data support the conclusions?

The manuscript must describe a technically sound piece of scientific research with data that supports the conclusions. Experiments must have been conducted rigorously, with appropriate controls, replication, and sample sizes. The conclusions must be drawn appropriately based on the data presented.

Reviewer #1: No

Reviewer #5: Yes

Reply to comment: We thank the reviewer.

________________________________________

3. Has the statistical analysis been performed appropriately and rigorously?

Reviewer #1: No

Reviewer #5: Yes

Reply to comment: We thank the reviewer.

________________________________________

4. Have the authors made all data underlying the findings in their manuscript fully available?

The PLOS Data policy requires authors to make all data underlying the findings described in their manuscript fully available without restriction, with rare exception (please refer to the Data Availability Statement in the manuscript PDF file). The data should be provided as part of the manuscript or its supporting information, or deposited to a public repository. For example, in addition to summary statistics, the data points behind means, medians and variance measures should be available. If there are restrictions on publicly sharing data—e.g. participant privacy or use of data from a third party—those must be specified.

Reviewer #1: Yes

Reviewer #5: Yes

Reply to comment: We thank the reviewers.

________________________________________

5. Is the manuscript presented in an intelligible fashion and written in standard English?

PLOS ONE does not copyedit accepted manuscripts, so the language in submitted articles must be clear, correct, and unambiguous. Any typographical or grammatical errors should be corrected at revision, so please note any specific errors here.

Reviewer #1: Yes

Reviewer #5: Yes

Reply to comment: We thank the reviewers.

________________________________________

6. Review Comments to the Author

Please use the space provided to explain your answers to the questions above. You may also include additional comments for the author, including concerns about dual publication, research ethics, or publication ethics. (Please upload your review as an attachment if it exceeds 20,000 characters)

Reviewer #1: Unfortunately, the authors did not address my concern regarding internal inconsistencies in Table 1. They have corrected the sum of cesareans however did not reviewed the whole table.

In several places, the absolute numbers and percentages are mismatched when back-calculated from. This suggests that either the denominators used for percentages varied or there were data omissions not clarified in the manuscript.

These inconsistencies, collectively reduce confidence in the accuracy of the data presentation. Clarification from the authors is necessary to ensure transparency and reproducibility.

For example:

• Study group (wartime,reported N = 801)

• Normal vaginal delivery: 475

• Vacuum extraction: 52

• Forceps delivery: 4

• Cesarean section: 223

• Subtotal = 754

• Missing cases: 47 deliveries unaccounted for

• Control group (N = 913)

• Normal vaginal delivery: 576

• Vacuum extraction: 42

• Forceps delivery: 2

• Cesarean section: 300

• Subtotal = 920

• Exceeds N by 7 cases

This affects all calculations and statistical analysis:

For example, the percentage of normal vaginal delivery should be approximately 62.99% rather than the reported 59%. And similar inconveniences are present throughout the table.

A comprehensive re-analysis of the data presented in Table 1 is strongly advised to ensure the reliability, accuracy, and reproducibility of the reported findings.

The current version contains substantive internal inconsistencies, most notably in the mode of delivery distributions, where subgroup counts do not correspond with the stated sample sizes, resulting in inaccurate percentages. Additionally, the hospitalization duration data require clarification. The overall interquartile ranges do not align coherently with those of the subgroups, raising questions about the analytic methodology and whether operative vaginal deliveries were categorized consistently. Such discrepancies diminish the interpretability and reliability of the reported findings.

Reply to comment: We thank the reviewer. Re-analysis of Table 1 was made as requested.

Minor Comments:

•The manuscript notes that parturients were offered discharge after 24 hours; however, it remains unclear whether this reflected a formal early-discharge policy or simply a theoretical option. The reported medians and IQRs approximate 48 hours, which aligns more closely with the standard recommended discharge interval at this center. Clarification on this point is warranted.

Reply to comment: Following delivery, low-risk patients were offered to be discharged earlier from the hospital after careful examination of them and their newborns. This sentence is in the Method section.

•In Table 1, the label “after cesarean” appears duplicated in the IQR row and should be corrected.

Reply to comment: Changes were made as suggested.

•In the legends, the term should be revised to “breech.”

Reply to comment: Changes were made as suggested.

Reviewer #5: All replies to the reviewers' comments were received. However, there one last issue that need to be adjusted.

1) Please adjust the references regarding the Plos One guideline.

Reply to comment: Changes were made as suggested.

The remaining questions have all been addressed and I believe that after these

adjustments, the article can be accepted for publication.

________________________________________

7. PLOS authors have the option to publish the peer review history of their article (what does this mean?). If published, this will include your full peer review and any attached files.

If you choose “no”, your identity will remain anonymous but your review may still be made public.

Do you want your identity to be public for this peer review? For information about this choice, including consent withdrawal, please see our Privacy Policy.

Reviewer #1: No

Reviewer #5: Yes: Vicente Sperb Antonello

# Peer review: Decision Letter 3

**DOI:** 10.1371/journal.pone.0335688.r007

**Authors:** Ahmed Maged

2 Dec 2025

Dear Dr. Weissmann-Brenner,

Thank you for submitting your manuscript to PLOS ONE. After careful consideration, we feel that it has merit but does not fully meet PLOS ONE’s publication criteria as it currently stands. Therefore, we invite you to submit a revised version of the manuscript that addresses the points raised during the review process.

==============================

**ACADEMIC EDITOR: Please respond to all reviewers comments**

Please submit your revised manuscript by Jan 16 2026 11:59PM. If you will need more time than this to complete your revisions, please reply to this message or contact the journal office at plosone@plos.org. When you're ready to submit your revision, log on to https://www.editorialmanager.com/pone/ and select the 'Submissions Needing Revision' folder to locate your manuscript file.

- A rebuttal letter that responds to each point raised by the academic editor and reviewer(s). You should upload this letter as a separate file labeled 'Response to Reviewers'.
- A marked-up copy of your manuscript that highlights changes made to the original version. You should upload this as a separate file labeled 'Revised Manuscript with Track Changes'.
- An unmarked version of your revised paper without tracked changes. You should upload this as a separate file labeled 'Manuscript'.

If you would like to make changes to your financial disclosure, please include your updated statement in your cover letter. Guidelines for resubmitting your figure files are available below the reviewer comments at the end of this letter.

If applicable, we recommend that you deposit your laboratory protocols in protocols.io to enhance the reproducibility of your results. Protocols.io assigns your protocol its own identifier (DOI) so that it can be cited independently in the future. For instructions see: https://journals.plos.org/plosone/s/submission-guidelines#loc-laboratory-protocols. Additionally, PLOS ONE offers an option for publishing peer-reviewed Lab Protocol articles, which describe protocols hosted on protocols.io. Read more information on sharing protocols at https://plos.org/protocols?utm_medium=editorial-email&utm_source=authorletters&utm_campaign=protocols.

We look forward to receiving your revised manuscript.

Kind regards,

Ahmed Mohamed Maged, MD

Academic Editor

PLOS ONE

Journal Requirements:

If the reviewer comments include a recommendation to cite specific previously published works, please review and evaluate these publications to determine whether they are relevant and should be cited. There is no requirement to cite these works unless the editor has indicated otherwise.

[Note: HTML markup is below. Please do not edit.]

Reviewers' comments:

Reviewer's Responses to Questions

**Comments to the Author**

Reviewer #3: All comments have been addressed

Reviewer #7: All comments have been addressed

Reviewer #8: (No Response)

Reviewer #9: All comments have been addressed

**********

2. Is the manuscript technically sound, and do the data support the conclusions?

Reviewer #3: Yes

Reviewer #7: Yes

Reviewer #8: Partly

Reviewer #9: No

**********

3. Has the statistical analysis been performed appropriately and rigorously?

Reviewer #3: Yes

Reviewer #7: No

Reviewer #8: Yes

Reviewer #9: No

**********

4. Have the authors made all data underlying the findings in their manuscript fully available?

The PLOS Data policy

Reviewer #3: No

Reviewer #7: (No Response)

Reviewer #8: No

Reviewer #9: (No Response)

**********

5. Is the manuscript presented in an intelligible fashion and written in standard English?

Reviewer #3: Yes

Reviewer #7: Yes

Reviewer #8: Yes

Reviewer #9: (No Response)

**********

Reviewer #3: This manuscript seems okay for submission as the author's have attended to the comments as seen from the abstract.I don't have access to the rest of the manuscript.

Reviewer #7: Dear Editor,

I write to submit my review on the manuscript titled “Early Hospital Discharge Following Delivery During Wartime is not Associated with Increased Rates of Postpartum Hospital Referrals.”

The study compared the rate of postpartum visits to the gynecological-emergency-room (GER) between wartime to a similar period in the previous year.

Here are my comments

Methods: What was the entire duration of the war? This must be clearly stated in the manuscript. Did the war only last for one month? Why did the authors decide to only compare outcomes within just one month of war? This could explain why no significant differences are seen in most of the outcomes. There may be a lagged effect of war, that is, the true effect of the war may even be experienced post-war. Since authors are only analyzing secondary data, a sensitivity analysis could have been conducted by expanding the scope to include 6 months before and 6 months after the war to assess whether the effect would be the same as evaluating it within one month. The reasoning behind selecting one month as the duration of assessment should be clearly elaborated in the manuscript if further analyses cannot be performed

Methods: Kindly define low-risk patients

Methods: The primary exposure of this study is war ( a binary indicator, before coded 0 and after war coded 1). Please include a section that outlines the outcome measures of interest and their corresponding measurement scales (e.g., continuous, binary, count, nominal, multinomial). Another section, called 'confounders, mediators,' should be clearly labeled. This will help assess whether the statistical methods chosen were appropriate. The variables are lumped together, including the sociodemographic factors which I believe are potential confounding factors.

Methods: The section titled “Descriptive statistics and analysis” are all analyses. Kindly title that section “Statistical Analysis”. Descriptive and inferential statistics are part of statistical analysis.

Methods: The statement “In order to compare post-partum visits to the GER in the two periods while controlling for potential confounders, a multivariable logistic regression model was fitted to the data, with age at delivery, gestational week (below or above 37), fetus 137 count (one or more), number of previous births (1,2,3,4, 5+)”. The statement looks incomplete. That is ...... with age at delivery, gestational week (below or above 37), fetus 137 count (one or more), number of previous births (1,2,3,4, 5+) as what? Confounders?. Kindly check

Methods: The methods sound a bit confusing. Some outcomes were treated as time to an event, “time until a post-partum referral,” and the authors compared the distribution of survival time (time until a post-partum referral) using the log-rank test after the Kaplan-Meier curve. Ideally, the Cox proportional hazard model would have been fitted to determine the hazard of war on postpartum referral, controlling for other potential confounders. The odds ratio from the multivariable logistic regression model could then serve as a sensitivity analysis. But the Cox proportional hazard model was not fitted.

Results: Table 1: Some of the expected frequencies were less than one (No. of fetuses vs wartime status) but reported p-values from the Chi-square test of independence instead of Fisher’s exact test.

Table 2: Must be restructured. Very confusing. Is it 70(8.7%) and 80(8.8%). Creating another column for the percentages makes it difficult to understand. No need.

Reviewer #8: (No Response)

Reviewer #9: Comments.

*Title

* Running title

*Abstract

- All abbreviations should have been provided in full on first mention and this applies to the title, running (short) title, abstract, impact statement, main text and each table / figure independently as they will be read independently. Please use the abbrevations correctly and effectively so all the abbrevations should be checked.

- The sstudy groups should be defined and also should be given the number of the participants for each group.

- Please do not to start to sentences with figures or numbers.

- Please determine the statistically significant of p values (p<0.05 or p<0.001 or p<0.0001).

- The real p values should be given.

- The abstract must contain enough data (figures) to enable the reader (researcher) to judge its content and therefore relevance. It can not include undefined terms or contradictory statements.

- The manuscript should have ended with a clear conclusion that sums up the value of the study within its limitations.

*Keywords

- The keywords should be added according to alphabetical order and they should be compatible with MeSH.

* Introduction

- An introduction should include information on why the subject under investigation is important, what is currently known on the topic and how the current investigation will improve our knowledge.

*Materials and Methods

- Statistical analysis method should be detailed. "Which test used for to determine distribution?, which test used for normal distribution and non-normal distribution. (Ideally, the distribution type and the statistical test used should be specified for each parameter). Kolmogorov-Smirnov test should be used to determine the distribution of the variables.

- The parameters distributed with normally distribution t test should be used, the parameters distributed with non-normally distribution should be used Mann-Whitney U test should be used.

- Which tests were used for categorical variables? The conditions for a valid chi-squared test and the circumstances when Fisher’s exact test can be used should have been specified in the method as well.

- The parameters distributed with normally distribution should be explained Mean+SD, the parameters distributed with non-normally distribution should be explained Median (min-max) or ideally median (25th-75th percentiles).

- Multivariate analysis should be performed to exclude possible confounding fctors.

*Results

- This section must contain enough data (figures) to enable the reader (researcher) to judge its content and therefore relevance. It can not include undefined terms or contradictory statements.

*Tables and Figures

* Discussion

- Practical implications and future research direction are not mentioned.

- In the discussion the authors should summarise and qualify their findings, compare outcomes with other publications and draw conclusion. The implications of the study and the future direction of study should be discussed. Importantly the study’s limitations should be considered.

- The manuscript should have ended with a clear conclusion that sums up the value of the study within its limitations.

- Post-hoc power analysis should be performed to exclude small sample size as a limitation.

*References

- The references should be cited the articles published in the last five years to progress the science.

***** All abbreviations should have been provided in full on first mention and this applies to the title, running (short) title, abstract, impact statement, main text and each table / figure independently as they will be read independently. Please use the abbrevations correctly and effectively so all the abbrevations should be checked.

***** Language and presentation require improvement.

***** Need several grammar/wording corrections (There are a lot of words with no space between them, there are missing spaces after commasin the manuscript and also, there are a lot of gramatical error. Language could be improved).

**********

what does this mean?). If published, this will include your full peer review and any attached files.

If you choose “no”, your identity will remain anonymous but your review may still be made public.

**Do you want your identity to be public for this peer review?**  For information about this choice, including consent withdrawal, please see our Privacy Policy

Reviewer #3: No

Reviewer #7: No

Reviewer #8: **Yes:** Precious L Hajison

Reviewer #9: No

**********

[NOTE: If reviewer comments were submitted as an attachment file, they will be attached to this email and accessible via the submission site. Please log into your account, locate the manuscript record, and check for the action link "View Attachments". If this link does not appear, there are no attachment files.]

To ensure your figures meet our technical requirements, please review our figure guidelines: https://journals.plos.org/plosone/s/figures

You may also use PLOS’s free figure tool, NAAS, to help you prepare publication quality figures: https://journals.plos.org/plosone/s/figures#loc-tools-for-figure-preparation.

NAAS will assess whether your figures meet our technical requirements by comparing each figure against our figure specifications.

Attachment Submitted filename: Reviewer Early discharge Plos.docx

# Author comment: Author response to Decision Letter 4

**DOI:** 10.1371/journal.pone.0335688.r008

16 Jan 2026

Date: Tuesday, January 13th, 2026

To: Ahmed Mohamed Maged, MD

Academic Editor

PLOS ONE

Re: PONE-D-24-57057 entitled:

Early Hospital Discharge Following Delivery During Wartime is not Associated with Increased Rates of Postpartum Hospital Referrals

PLOS ONE

Dear Dr. Ahmed Mohamed Maged,

We would like to thank you, the editor of PLOS ONE Journal, and the reviewers again for considering our manuscript for PLOS ONE Journal and for your time and efforts to review this manuscript.

We have addressed each comment in the following reply and have revised the manuscript accordingly. We hope that the 4th revised version of the manuscript is considered suitable for publication in PLOS ONE Journal.

An itemized reply to the comments of the Reviewers is provided below.

Yours sincerely,

Prof. Alina Weissmann-Brenner.

Reviewers' comments:

1. If the authors have adequately addressed your comments raised in a previous round of review and you feel that this manuscript is now acceptable for publication, you may indicate that here to bypass the “Comments to the Author” section, enter your conflict of interest statement in the “Confidential to Editor” section, and submit your "Accept" recommendation.

Reviewer #3: All comments have been addressed

Reviewer #7: All comments have been addressed

Reviewer #8: (No Response)

Reviewer #9: All comments have been addressed

Reply to comment: We thank the reviewers.

________________________________________

2. Is the manuscript technically sound, and do the data support the conclusions?

The manuscript must describe a technically sound piece of scientific research with data that supports the conclusions. Experiments must have been conducted rigorously, with appropriate controls, replication, and sample sizes. The conclusions must be drawn appropriately based on the data presented.

Reviewer #3: Yes

Reviewer #7: Yes

Reviewer #8: Partly

Reviewer #9: No

Reply to comment: We thank the reviewers.

________________________________________

3. Has the statistical analysis been performed appropriately and rigorously?

Reviewer #3: Yes

Reviewer #7: No

Reviewer #8: Yes

Reviewer #9: No

Reply to comment: We thank the reviewers.

________________________________________

4. Have the authors made all data underlying the findings in their manuscript fully available?

The PLOS Data policy requires authors to make all data underlying the findings described in their manuscript fully available without restriction, with rare exception (please refer to the Data Availability Statement in the manuscript PDF file). The data should be provided as part of the manuscript or its supporting information, or deposited to a public repository. For example, in addition to summary statistics, the data points behind means, medians and variance measures should be available. If there are restrictions on publicly sharing data—e.g. participant privacy or use of data from a third party—those must be specified.

Reviewer #3: No

Reviewer #7: (No Response)

Reviewer #8: No

Reviewer #9: (No Response)

Reply to comment: We thank the reviewers.

________________________________________

5. Is the manuscript presented in an intelligible fashion and written in standard English?

PLOS ONE does not copyedit accepted manuscripts, so the language in submitted articles must be clear, correct, and unambiguous. Any typographical or grammatical errors should be corrected at revision, so please note any specific errors here.

Reviewer #3: Yes

Reviewer #7: Yes

Reviewer #8: Yes

Reviewer #9: (No Response)

Reply to comment: We thank the reviewers.

________________________________________

6. Review Comments to the Author

Please use the space provided to explain your answers to the questions above. You may also include additional comments for the author, including concerns about dual publication, research ethics, or publication ethics. (Please upload your review as an attachment if it exceeds 20,000 characters)

Reviewer #3: This manuscript seems okay for submission as the author's have attended to the comments as seen from the abstract.I don't have access to the rest of the manuscript.

Reply to comment: We thank the reviewers.

Reviewer #7: Dear Editor,

I write to submit my review on the manuscript titled “Early Hospital Discharge Following Delivery During Wartime is not Associated with Increased Rates of Postpartum Hospital Referrals.”

The study compared the rate of postpartum visits to the gynecological-emergency-room (GER) between wartime to a similar period in the previous year.

Here are my comments

Methods: What was the entire duration of the war? This must be clearly stated in the manuscript. Did the war only last for one month? Why did the authors decide to only compare outcomes within just one month of war? This could explain why no significant differences are seen in most of the outcomes. There may be a lagged effect of war, that is, the true effect of the war may even be experienced post-war. Since authors are only analyzing secondary data, a sensitivity analysis could have been conducted by expanding the scope to include 6 months before and 6 months after the war to assess whether the effect would be the same as evaluating it within one month. The reasoning behind selecting one month as the duration of assessment should be clearly elaborated in the manuscript if further analyses cannot be performed

Reply to comment:

The war started with a surprise attack launched by Hamas on Saturday morning, Simchat Torah, October 7, 2023. In this attack, Hamas terrorists invaded IDF bases and civilian localities in the Gaza perimeter region, while firing thousands of missiles at Israel. About a day after the outbreak of the war, the Hezbollah terrorist organization fired missiles from Lebanon to the northern part of Israel. Iran also launched missile attacks on Israel on 04/24, 10/24 and 06/25. Ceasefire was achieved on 19/01/25.

On October 2023 there were 8500 missiles that were launched towards Israel, on November 2023 there were 2000missiles, and on December 2023 there were 2000 missiles launched towards Israel.

We chose to concentrate on the first month of the war mainly because of the surprising nature of this war, necessitating rapid adaptations of the hospitals, and the intensity of the missile attacks in that month. This data was added to the Introduction, methods and Discussion sections.

Indeed, additional adaptations were made during the missile attacks from Iran, including transfer of the delivery rooms to underground secure locations. However, the main focus of the present study was to assess the shortening of the duration of hospitalization following delivery, that was relevant mainly in the first month of the war.

Methods: Kindly define low-risk patients

Methods: The primary exposure of this study is war ( a binary indicator, before coded 0 and after war coded 1). Please include a section that outlines the outcome measures of interest and their corresponding measurement scales (e.g., continuous, binary, count, nominal, multinomial). Another section, called 'confounders, mediators,' should be clearly labeled. This will help assess whether the statistical methods chosen were appropriate. The variables are lumped together, including the sociodemographic factors which I believe are potential confounding factors.

Reply to comment: The section about the variables assessed in the study was changed as requested.

Methods: The section titled “Descriptive statistics and analysis” are all analyses. Kindly title that section “Statistical Analysis”. Descriptive and inferential statistics are part of statistical analysis.

Methods: The statement “In order to compare post-partum visits to the GER in the two periods while controlling for potential confounders, a multivariable logistic regression model was fitted to the data, with age at delivery, gestational week (below or above 37), fetus 137 count (one or more), number of previous births (1,2,3,4, 5+)”. The statement looks incomplete. That is ...... with age at delivery, gestational week (below or above 37), fetus 137 count (one or more), number of previous births (1,2,3,4, 5+) as what? Confounders

Reply to comment: Correction was made. Thank you.

?. Kindly check

Methods: The methods sound a bit confusing. Some outcomes were treated as time to an event, “time until a post-partum referral,” and the authors compared the distribution of survival time (time until a post-partum referral) using the log-rank test after the Kaplan-Meier curve. Ideally, the Cox proportional hazard model would have been fitted to determine the hazard of war on postpartum referral, controlling for other potential confounders. The odds ratio from the multivariable logistic regression model could then serve as a sensitivity analysis. But the Cox proportional hazard model was not fitted.

Reply to comment: We thank the reviewer. Changes were made in the Method section as requested.

Results: Table 1: Some of the expected frequencies were less than one (No. of fetuses vs wartime status) but reported p-values from the Chi-square test of independence instead of Fisher’s exact test.

Reply to comment: A footnote was added to Table 1 about the comparison for number of fetuses as requested.

Table 2: Must be restructured. Very confusing. Is it 70(8.7%) and 80(8.8%). Creating another column for the percentages makes it difficult to understand. No need.

Reply to comment: We thank the reviewer. Changes were made in the Results section as requested. The columns in Table 2 were changed.

Reviewer #8: (No Response)

Reviewer #9: Comments.

*Title

* Running title

*Abstract

- All abbreviations should have been provided in full on first mention and this applies to the title, running (short) title, abstract, impact statement, main text and each table / figure independently as they will be read independently. Please use the abbrevations correctly and effectively so all the abbrevations should be checked.

Reply to comment: We thank the reviewer. We explained the abbreviations in the Abstract, Declaration and Results sections as requested.

- The sstudy groups should be defined and also should be given the number of the participants for each group.

Reply to comment: 801 women delivered in the first month of the war in 2023 and 913 delivered in the parallel period in the previous year.

- Please do not to start to sentences with figures or numbers.

Reply to comment: Changes were made as requested.

- Please determine the statistically significant of p values (p<0.05 or p<0.001 or p<0.0001).

- The real p values should be given.

Reply to comment: Due to limitations of the number of words in the Abstract section we cannot add the sentence that p value of <0.05 was considered statistically significant. This sentence appears in the method section.

- The abstract must contain enough data (figures) to enable the reader (researcher) to judge its content and therefore relevance. It can not include undefined terms or contradictory statements.

Reply to comment: Tables and Figure are embedded in the manuscript itself. If the Editor wishes we provide an Abstract with Figures we can changes the entire Abstract as requested.

- The manuscript should have ended with a clear conclusion that sums up the value of the study within its limitations.

Reply to comment: The Abstract ends with the conclusions of the manuscript. Due to limitations of the word count of the Journal the Limitations were specified in the Discussion section.

*Keywords

- The keywords should be added according to alphabetical order and they should be compatible with MeSH.

Reply to comment: We arranged the Key Words in alphabetical morder.

* Introduction

- An introduction should include information on why the subject under investigation is important, what is currently known on the topic and how the current investigation will improve our knowledge.

Reply to comment: The Introduction section was editted as requested.

*Materials and Methods

- Statistical analysis method should be detailed. "Which test used for to determine distribution?, which test used for normal distribution and non-normal distribution. (Ideally, the distribution type and the statistical test used should be specified for each parameter). Kolmogorov-Smirnov test should be used to determine the distribution of the variables.

- The parameters distributed with normally distribution t test should be used, the parameters distributed with non-normally distribution should be used Mann-Whitney U test should be used.

- Which tests were used for categorical variables? The conditions for a valid chi-squared test and the circumstances when Fisher’s exact test can be used should have been specified in the method as well.

- The parameters distributed with normally distribution should be explained Mean+SD, the parameters distributed with non-normally distribution should be explained Median (min-max) or ideally median (25th-75th percentiles).

- Multivariate analysis should be performed to exclude possible confounding fctors.

*Results

- This section must contain enough data (figures) to enable the reader (researcher) to judge its content and therefore relevance. It can not include undefined terms or contradictory statements.

Reply to comment: The Result section contains data of the results of the study including statements and Tables and Figure.

*Tables and Figures

* Discussion

- Practical implications and future research direction are not mentioned.

- In the discussion the authors should summarise and qualify their findings, compare outcomes with other publications and draw conclusion. The implications of the study and the future direction of study should be discussed. Importantly the study’s limitations should be considered.

- The manuscript should have ended with a clear conclusion that sums up the value of the study within its limitations.

Reply to comment: Discussion and conclusion sections were edited and changed as requested, including English corrections.

- Post-hoc power analysis should be performed to exclude small sample size as a limitation.

Reply to comment: Due to the retrospective nature of the study we did not perform a power analysis. Explanations about why the first 2 months of the war were chosen are explained in details in both manuscript and in the response to the reviewer's previous questions.

*References

- The references should be cited the articles published in the last five years to progress the science.

Reply to comment: There is lack of data regarding the subject of the manuscript, therefore references before 2020 were also added to the manuscript.

***** All abbreviations should have been provided in full on first mention and this applies to the title, running (short) title, abstract, impact statement, main text and each table / figure independently as they will be read independently. Please use the abbrevations correctly and effectively so all the abbrevations should be checked.

Reply to comment: Abbreviations were specified as requested.

***** Language and presentation require improvement.

***** Need several grammar/wording corrections (There are a lot of words with no space between them, there are missing spaces after commasin the manuscript and also, there are a lot of gramatical error. Language could be improved).

Reply to comment: Changes were made as suggested.

________________________________________

Attachment Submitted filename: 4th responce to reviewers.docx

# Peer review: Decision Letter 4

**DOI:** 10.1371/journal.pone.0335688.r009

**Authors:** Ahmed Maged

12 Feb 2026

Dear Dr. Weissmann-Brenner,

Thank you for submitting your manuscript to PLOS ONE. After careful consideration, we feel that it has merit but does not fully meet PLOS ONE’s publication criteria as it currently stands. Therefore, we invite you to submit a revised version of the manuscript that addresses the points raised during the review process.

==============================

**ACADEMIC EDITOR: Please respond to all reviewers comments**

Please submit your revised manuscript by Mar 29 2026 11:59PM. If you will need more time than this to complete your revisions, please reply to this message or contact the journal office at plosone@plos.org. When you're ready to submit your revision, log on to https://www.editorialmanager.com/pone/ and select the 'Submissions Needing Revision' folder to locate your manuscript file.

- A letter that responds to each point raised by the academic editor and reviewer(s). You should upload this letter as a separate file labeled 'Response to Reviewers'.
- A marked-up copy of your manuscript that highlights changes made to the original version. You should upload this as a separate file labeled 'Revised Manuscript with Track Changes'.
- An unmarked version of your revised paper without tracked changes. You should upload this as a separate file labeled 'Manuscript'.

If you would like to make changes to your financial disclosure, please include your updated statement in your cover letter. Guidelines for resubmitting your figure files are available below the reviewer comments at the end of this letter.

If applicable, we recommend that you deposit your laboratory protocols in protocols.io to enhance the reproducibility of your results. Protocols.io assigns your protocol its own identifier (DOI) so that it can be cited independently in the future. For instructions see: https://journals.plos.org/plosone/s/submission-guidelines#loc-laboratory-protocols. Additionally, PLOS ONE offers an option for publishing peer-reviewed Lab Protocol articles, which describe protocols hosted on protocols.io. Read more information on sharing protocols at https://plos.org/protocols?utm_medium=editorial-email&utm_source=authorletters&utm_campaign=protocols.

We look forward to receiving your revised manuscript.

Kind regards,

Ahmed Mohamed Maged, MD

Academic Editor

PLOS One

Journal Requirements:

If the reviewer comments include a recommendation to cite specific previously published works, please review and evaluate these publications to determine whether they are relevant and should be cited. There is no requirement to cite these works unless the editor has indicated otherwise.

[Note: HTML markup is below. Please do not edit.]

Reviewers' comments:

Reviewer's Responses to Questions

**Comments to the Author**

Reviewer #7: All comments have been addressed

Reviewer #8: All comments have been addressed

Reviewer #9: All comments have been addressed

**********

2. Is the manuscript technically sound, and do the data support the conclusions?

Reviewer #7: Yes

Reviewer #8: Yes

Reviewer #9: No

**********

3. Has the statistical analysis been performed appropriately and rigorously?

Reviewer #7: Yes

Reviewer #8: Yes

Reviewer #9: No

**********

4. Have the authors made all data underlying the findings in their manuscript fully available?

The PLOS Data policy

Reviewer #7: Yes

Reviewer #8: Yes

Reviewer #9: No

**********

5. Is the manuscript presented in an intelligible fashion and written in standard English?

Reviewer #7: Yes

Reviewer #8: Yes

Reviewer #9: No

**********

Reviewer #7: The authors have comprehensively addressed all the comments and concerns raised in my previous review of the manuscript

Reviewer #8: (No Response)

Reviewer #9: The artcile was not revised accordingly.

- Post-hoc power analysis should be performed to exclude small sample size as a limitation.

**********

what does this mean?). If published, this will include your full peer review and any attached files.

If you choose “no”, your identity will remain anonymous but your review may still be made public.

**Do you want your identity to be public for this peer review?**  For information about this choice, including consent withdrawal, please see our Privacy Policy

Reviewer #7: No

Reviewer #8: **Yes:** Precious L Hajison

Reviewer #9: No

**********

[NOTE: If reviewer comments were submitted as an attachment file, they will be attached to this email and accessible via the submission site. Please log into your account, locate the manuscript record, and check for the action link "View Attachments". If this link does not appear, there are no attachment files.]

To ensure your figures meet our technical requirements, please review our figure guidelines: https://journals.plos.org/plosone/s/figures

You may also use PLOS’s free figure tool, NAAS, to help you prepare publication quality figures: https://journals.plos.org/plosone/s/figures#loc-tools-for-figure-preparation.

NAAS will assess whether your figures meet our technical requirements by comparing each figure against our figure specifications.

# Author comment: Author response to Decision Letter 5

**DOI:** 10.1371/journal.pone.0335688.r010

4 Mar 2026

Date: Thursday, February 19, 2026

To: Ahmed Mohamed Maged, MD

Academic Editor

PLOS ONE

Re: PONE-D-24-57057 entitled:

Early Hospital Discharge Following Delivery During Wartime is not Associated with Increased Rates of Postpartum Hospital Referrals

PLOS ONE

Dear Dr. Ahmed Mohamed Maged,

We would like to thank you, the editor of PLOS ONE Journal, and the reviewers again for considering our manuscript for PLOS ONE Journal and for your time and efforts to review this manuscript.

We have addressed each comment in the following reply and have revised the manuscript accordingly. We hope that the 5th revised version of the manuscript is considered suitable for publication in PLOS ONE Journal.

An itemized reply to the comments of the Reviewers is provided below.

Yours sincerely,

Prof. Alina Weissmann-Brenner.

Reviewers' comments:

1. If the authors have adequately addressed your comments raised in a previous round of review and you feel that this manuscript is now acceptable for publication, you may indicate that here to bypass the “Comments to the Author” section, enter your conflict of interest statement in the “Confidential to Editor” section, and submit your "Accept" recommendation.

Reviewer #7: All comments have been addressed

Reviewer #8: All comments have been addressed

Reviewer #9: All comments have been addressed

Reply to comment: We thank the reviewers.

________________________________________

2. Is the manuscript technically sound, and do the data support the conclusions?

The manuscript must describe a technically sound piece of scientific research with data that supports the conclusions. Experiments must have been conducted rigorously, with appropriate controls, replication, and sample sizes. The conclusions must be drawn appropriately based on the data presented.

Reviewer #7: Yes

Reviewer #8: Yes

Reviewer #9: No

Reply to comment: We thank the reviewers.

________________________________________

3. Has the statistical analysis been performed appropriately and rigorously?

Reviewer #7: Yes

Reviewer #8: Yes

Reviewer #9: No

Reply to comment: We thank the reviewers. A post hoc power analysis was made. Data was added to the Method and the Discussion sections.

________________________________________

4. Have the authors made all data underlying the findings in their manuscript fully available?

The PLOS Data policy requires authors to make all data underlying the findings described in their manuscript fully available without restriction, with rare exception (please refer to the Data Availability Statement in the manuscript PDF file). The data should be provided as part of the manuscript or its supporting information, or deposited to a public repository. For example, in addition to summary statistics, the data points behind means, medians and variance measures should be available. If there are restrictions on publicly sharing data—e.g. participant privacy or use of data from a third party—those must be specified.

Reviewer #7: Yes

Reviewer #8: Yes

Reviewer #9: No

Reply to comment: We thank the reviewers.

________________________________________

5. Is the manuscript presented in an intelligible fashion and written in standard English?

PLOS ONE does not copyedit accepted manuscripts, so the language in submitted articles must be clear, correct, and unambiguous. Any typographical or grammatical errors should be corrected at revision, so please note any specific errors here.

Reviewer #7: Yes

Reviewer #8: Yes

Reviewer #9: No

Reply to comment: We thank the reviewers.

________________________________________

6. Review Comments to the Author

Please use the space provided to explain your answers to the questions above. You may also include additional comments for the author, including concerns about dual publication, research ethics, or publication ethics. (Please upload your review as an attachment if it exceeds 20,000 characters)

Reviewer #7: The authors have comprehensively addressed all the comments and concerns raised in my previous review of the manuscript

Reviewer #8: (No Response)

Reviewer #9: The artcile was not revised accordingly.

- Post-hoc power analysis should be performed to exclude small sample size as a limitation.

Reply to comment: We thank the reviewers. A post hoc power analysis was made. Data was added to the Method and the Discussion sections.

________________________________________

Attachment Submitted filename: 5th responce to reviewers.docx

# Peer review: Decision Letter 5

**DOI:** 10.1371/journal.pone.0335688.r011

**Authors:** Danish Ahmad

11 May 2026

Dear Dr. Weissmann-Brenner,

Thank you for submitting your manuscript to PLOS ONE. After careful consideration, we feel that it has merit but does not fully meet PLOS ONE’s publication criteria as it currently stands. Therefore, we invite you to submit a revised version of the manuscript that addresses the points raised during the review process.

We recognise that this manuscript has now been through five rounds of peer review, with a mixed pattern of recommendations across prior rounds  some reviewers having recommended acceptance, others rejection  and considerable variation in the depth of engagement across reviews. In my capacity as handling editor since April 26, I have therefore consolidated the outstanding matters into the single structured set of comments that follows. These are intended to cover both unresolved points raised by previous reviewers and new issues that emerge from a careful reading of the current version. In the interest of bringing this review process to a timely and decisive resolution, subsequent rounds will be handled directly between the handling editor(& the journal) and the authors as adequate revisions are completed, rather than returning the manuscript to external reviewers. This is intended to spare the authors further protracted iteration and to allow the editorial process to conclude on the merits of a substantively revised manuscript.

Please submit your revised manuscript by Jun 25 2026 11:59PM. If you will need more time than this to complete your revisions, please reply to this message or contact the journal office at plosone@plos.org. When you're ready to submit your revision, log on to https://www.editorialmanager.com/pone/ and select the 'Submissions Needing Revision' folder to locate your manuscript file.

- A letter that responds to each point raised by the academic editor and reviewer(s). You should upload this letter as a separate file labeled 'Response to Reviewers'.
- A marked-up copy of your manuscript that highlights changes made to the original version. You should upload this as a separate file labeled 'Revised Manuscript with Track Changes'.
- An unmarked version of your revised paper without tracked changes. You should upload this as a separate file labeled 'Manuscript'.

If you would like to make changes to your financial disclosure, please include your updated statement in your cover letter. Guidelines for resubmitting your figure files are available below the reviewer comments at the end of this letter.

If applicable, we recommend that you deposit your laboratory protocols in protocols.io to enhance the reproducibility of your results. Protocols.io assigns your protocol its own identifier (DOI) so that it can be cited independently in the future. For instructions see: https://journals.plos.org/plosone/s/submission-guidelines#loc-laboratory-protocols. Additionally, PLOS ONE offers an option for publishing peer-reviewed Lab Protocol articles, which describe protocols hosted on protocols.io. Read more information on sharing protocols at https://plos.org/protocols?utm_medium=editorial-email&utm_source=authorletters&utm_campaign=protocols.

As the corresponding author, your ORCID iD is verified in the submission system and will appear in the published article. PLOS supports the use of ORCID, and we encourage all coauthors to register for an ORCID iD and use it as well. Please encourage your coauthors to verify their ORCID iD within the submission system before final acceptance, as unverified ORCID iDs will not appear in the published article. *Only*  the individual author can complete the verification step; PLOS staff *cannot*  verify ORCID iDs on behalf of authors.

We look forward to receiving your revised manuscript.

Kind regards,

Danish Ahmad, MBBS,MSc,MNAMS,PhD,IP-FPH(UK),FRCP(Edin),FRCP(Lon)

Academic Editor

PLOS One

Journal Requirements:

If the reviewer comments include a recommendation to cite specific previously published works, please review and evaluate these publications to determine whether they are relevant and should be cited. There is no requirement to cite these works unless the editor has indicated otherwise.

Additional Editor Comments:

**Overview**

The manuscript addresses a clinically and policy-relevant question whether the introduction of an early-discharge protocol during wartime altered the rate or pattern of postpartum gynaecological emergency room (GER) presentations. The study design is sound in its core elements: the holiday-shifted control window is a thoughtful bias control, the mediator/confounder distinction in the model specification is correctly handled in principle, and the analytical strategy of logistic regression with a Cox proportional-hazards sensitivity analysis is appropriate for fixed-window follow-up data. The authors should be commended on these elements.

That said, several issues remain after the previous round. The comments below identify gaps in framing, language, cohort description, confounder selection, interpretation of borderline findings, and reporting standards. A number of these are substantive and, in our view, must be addressed before the manuscript is suitable for publication.

Introduction

1. Lines 75–93 focus the opening and disambiguate "war" from "disaster". These lines read as a broad scene-setting passage and do not introduce the study's obstetric/maternal-health focus early enough. Please open with a more focused topic sentence, and delete lines 75–83. A related issue runs through the manuscript: war and disaster are not interchangeable terms  they have distinct definitions and produce different health system impacts. As the study is set in a conflict context rather than a natural-disaster context, please use war / armed conflict terminology consistently throughout.

2. Lines 110–112 relocate or delete. These lines sit awkwardly in the introduction. Their generalising claim that the findings may be relevant to conflicts and disasters more broadly belongs in the discussion or conclusion, after the specific findings have been presented. Please consider deleting these lines from the introduction or relocating them.

3. Introduce Israel’s health-system context early, not in the limitations. A point currently raised only in the limitations should be introduced in the introduction or early methods: Israel is a high-income setting with mature emergency and trauma care infrastructure, shaped both by repeated exposure to armed conflict and by substantial domestic investment in trauma medicine. Establishing this context early frames the generalisability of the findings appropriately for an international readership.

4. Lines 94–98, 119–120, and 298–299 adopt objective scientific language and add specific contextual anchors. These lines describe the events of 7 October and the resulting loss of life. While the gravity of those events is fully acknowledged, a scientific manuscript for an international audience should present this background in objective language, without evaluative adjectives and without details that do not bear directly on the study aim or methods. Elements such as the regional evacuation are worth retaining because they describe a population-level shift relevant to the analysis. The authors should also state explicitly that, following the attack on 7 October, Israel formally declared a state of war on 8 October, and that the present study covers only the first month of that period.

5. Clarify the scope of the early-discharge protocol. Please clarify whether the early-discharge protocol described in the methods is specific to the study centre, or whether it reflects a national directive issued in response to the war declaration. This affects how readers will interpret generalisability.

6. Outcome ascertainment and post-discharge follow-up. Reviewer 1 raised this in the previous round and the response was not, in our view, sufficient. Please clarify (a) whether discharged women received structured follow-up for example, telephone contact or written instructions identifying warning signs and points of presentation — and (b) how the authors are confident that women experiencing complications were captured by the study, given they could plausibly have presented to emergency departments outside the study site (particularly relevant in the context of regional evacuations). If outside-site presentations cannot be ascertained from the available data, this must be stated explicitly as a limitation affecting outcome ascertainment.

Methods and Results

7. Table 2 interpretation of time from delivery to GER referral. The reported time from delivery to GER referral appears shorter in the wartime cohort (median 5.9 [IQR 4.5–10.6]) than in the control (median 7.5 [IQR 4.5–16.0]), with a Wilcoxon p-value of 0.16, yet the text states there was no significant difference in the time elapsed from delivery to postpartum referral. The point estimate is approximately 1.6 days earlier presentation in the wartime cohort, with a meaningfully wider tail in the control cohort. This is precisely the pattern that would be expected if early-discharged women re-present sooner because they are sent home before the natural window for early postpartum complications has closed. Characterising this as "no significant difference" obscures a clinically interpretable signal. Please:

(a) confirm whether the values reported are means or medians, with corresponding SD or IQR, and report the exact test used and p-value;

(b) discuss whether the apparent ~1.6-day earlier presentation, even if not statistically significant, is clinically meaningful  that is, whether it represents a signal that women discharged earlier under the wartime protocol required postpartum care sooner than the control cohort.

A non-significant p-value with a clinically suggestive point estimate warrants discussion rather than dismissal.

8. Confounders and mediators  terminology. The terms confounder and mediator are used inconsistently across the manuscript. These are distinct epidemiological constructs and should not be used interchangeably. The methods correctly identify delivery characteristics and length-of-stay as mediators on the causal pathway, but elsewhere in the text the language slips. Please audit the manuscript for consistency and tighten it throughout.

09. Discuss the shift in the elective caesarean fraction (Table 1). Table 1 reports that 57% of caesarean sections in the wartime cohort were classified as elective, compared with 92% in the control period (p < 0.0001). This 35-percentage-point shift is the single largest difference between the two cohorts and is currently mentioned only in passing. Three interpretations are possible and have substantially different implications for how the null finding on GER referral should be read:

(a) Elective caesareans were rationally deferred during wartime for resource or scheduling reasons, a parallel intervention, separate from early discharge.

(b) The wartime cohort experienced a genuine increase in obstetric complications, driving non-elective and urgent caesareans  an effect of war that should be reflected in other outcomes;

(c) The population delivering during wartime differed from the control population due to regional evacuations or altered referral patterns,  a case-mix shift that the current confounder set does not capture.

The authors are asked to discuss this finding explicitly in the manuscript

10. Cohort derivation is not described and should be reported in STROBE-compliant form. The methods state that all deliveries within each study window were included, but the manuscript provides no inclusion/exclusion algorithm, no flow diagram, and no information on exclusions for fetal demise, antenatal transfer, incomplete electronic medical record data, or other reasons. STROBE adherence is not stated. For an observational cohort study using administrative data, this is a substantial reporting gap. Please provide a STROBE flow diagram and a clear inclusion/exclusion statement, and add a STROBE adherence statement to the methods.

11. Specify the operational definition of "low-risk" and consider an as-treated analysis. The definition of patients eligible for early discharge "generally healthy women, with no delivery complications such as profound vaginal bleeding, hypertension or infection"  is clinical rather than operational. The manuscript does not specify (a) the criteria that determined eligibility for early discharge in practice, (b) whether pre-existing maternal conditions (diabetes, mental health history, BMI) affected eligibility, or (c) what proportion of the wartime cohort actually received the abbreviated stay. This matters because the analysis treats the entire wartime cohort as exposed to the early-discharge intervention, when in reality only a subset would have been eligible. Please specify the operational criteria, report the proportion of each cohort that received early discharge, and present an as-treated sensitivity analysis restricted to women who actually received the abbreviated stay.

12. Reasons-for-GER analysis classifies only a minority of referrals. The analysis of indications for postpartum GER referral classifies only infection (11 + 13 = 24 cases) and bleeding (0 + 4 = 4 cases), totalling 28 of the 150 GER referrals  approximately 19% of all cases. The remaining ~120 referrals are not categorised. The statement that "there were also no significant differences in the reasons for the postpartum referrals" cannot be supported when the majority of referrals have not been classified. Please either (a) provide a complete categorisation of all GER referrals by indication, with appropriate adjustment for multiple comparisons, or (b) remove this analysis from the manuscript.

13. The confounder set is thin for a wartime/peacetime comparison. The multivariable models adjust for age at delivery, gestational week (<37 vs ≥37), fetus count, and parity. The exclusion of delivery characteristics and length of stay on the grounds that they are mediators is appropriate. However, several variables that are plausibly associated with both calendar period and the outcome, and which are not on the mediator pathway, have been omitted without justification:

(a) Maternal comorbidities eg. pre-existing hypertension, diabetes, obesity, thyroid disease, and mental health history. These conditions pre-date the exposure window and are established predictors of postpartum complications and ED re-presentation.

(b) Geographic and socioeconomic variables area of residence, distance to the index hospital, and any indicator of displacement status. Specifically in the wartime cohort, internal displacement following regional evacuations is a likely source of confounding that the current model cannot address.

Please explicitly acknowledge in the limitations the residual confounding that remains if these variables are not available in the dataset.

14. "No difference" framing overstates the precision of the estimates. The manuscript repeatedly characterises the findings as showing "no difference" between the wartime and control periods. This phrasing edges toward an equivalence claim that the data do not support. The 95% confidence interval for the multivariable odds ratio (0.72–1.41) is compatible with up to a 28% reduction or a 41% increase in the odds of postpartum GER referral. The study has ruled out only large effects; clinically and policy-relevant moderate effects in either direction remain plausible. The post hoc power statement implicitly acknowledges this, yet the language of the discussion is not consistent with it. A more accurate framing throughout, particularly in the abstract, results, and conclusion, would be: "We found no evidence of a difference between the wartime and control periods, with confidence intervals compatible with moderate increases or decreases in postpartum GER risk." Equivalence cannot be claimed without a pre-specified equivalence margin and the corresponding test, which were not performed.

15. Smaller methodological and reporting points. A number of additional points should be addressed:

(a) The methods (line 142) define the postpartum period as 42 days, while Figure 1 plots 45 days. Please reconcile.

(b) Missing data handling is not described. Please specify whether complete-case analysis was used and report the extent of missingness for each variable in the analytical model.

(d) p-values reported as exactly "1" in Table 2 should be reported as ">0.99" to avoid the misleading impression of perfect agreement.

(e) The single-centre, tertiary-hospital design limits generalisability to other Israeli hospitals — particularly those serving evacuated regions — and to other conflict settings. This should be acknowledged more prominently in the limitations.

Reviewers' comments:

Reviewer's Responses to Questions

**Comments to the Author**

Reviewer #7: All comments have been addressed

**********

2. Is the manuscript technically sound, and do the data support the conclusions?

Reviewer #7: Yes

**********

3. Has the statistical analysis been performed appropriately and rigorously?

Reviewer #7: Yes

**********

4. Have the authors made all data underlying the findings in their manuscript fully available?

The PLOS Data policy

Reviewer #7: Yes

**********

5. Is the manuscript presented in an intelligible fashion and written in standard English?

Reviewer #7: Yes

**********

Reviewer #7: The authors have comprehensively addressed all the comments and concerns raised in my previous review of the manuscript

**********

what does this mean?). If published, this will include your full peer review and any attached files.

If you choose “no”, your identity will remain anonymous but your review may still be made public.

**Do you want your identity to be public for this peer review?**  For information about this choice, including consent withdrawal, please see our Privacy Policy

Reviewer #7: No

**********

[NOTE: If reviewer comments were submitted as an attachment file, they will be attached to this email and accessible via the submission site. Please log into your account, locate the manuscript record, and check for the action link "View Attachments". If this link does not appear, there are no attachment files.]

To ensure your figures meet our technical requirements, please review our figure guidelines: https://journals.plos.org/plosone/s/figures

You may also use PLOS’s free figure tool, NAAS, to help you prepare publication quality figures: https://journals.plos.org/plosone/s/figures#loc-tools-for-figure-preparation.

NAAS will assess whether your figures meet our technical requirements by comparing each figure against our figure specifications.

# Author comment: Author response to Decision Letter 6

**DOI:** 10.1371/journal.pone.0335688.r012

3 Jun 2026

Date: Wednesday, June 3, 2026

To: Danish Ahmad, , MBBS,MSc,MNAMS,PhD,IP-H(UK),FRCP(Edin),FRCP(Lon)

Academic Editor

PLOS ONE

Re: PONE-D-24-57057 entitled:

Early Hospital Discharge Following Delivery During Wartime is not Associated with Increased Rates of Postpartum Hospital Referrals,

Dear Dr. Danish Ahmad,

We would like to thank you, the editor of PLOS ONE Journal for considering our manuscript for PLOS ONE Journal and for your time and efforts to review this manuscript.

We have addressed each comment in the following reply and have revised the manuscript accordingly. We hope that the 6h revised version of the manuscript is considered suitable for publication in PLOS ONE Journal.

An itemized reply to the comments of the Reviewers is provided below.

Yours sincerely,

Prof. Alina Weissmann-Brenner.

Reviewers' comments:

Overview

The manuscript addresses a clinically and policy-relevant question whether the introduction of an early-discharge protocol during wartime altered the rate or pattern of postpartum gynaecological emergency room (GER) presentations. The study design is sound in its core elements: the holiday-shifted control window is a thoughtful bias control, the mediator/confounder distinction in the model specification is correctly handled in principle, and the analytical strategy of logistic regression with a Cox proportional-hazards sensitivity analysis is appropriate for fixed-window follow-up data. The authors should be commended on these elements.

That said, several issues remain after the previous round. The comments below identify gaps in framing, language, cohort description, confounder selection, interpretation of borderline findings, and reporting standards. A number of these are substantive and, in our view, must be addressed before the manuscript is suitable for publication.

Introduction

1. Lines 75–93 focus the opening and disambiguate "war" from "disaster". These lines read as a broad scene-setting passage and do not introduce the study's obstetric/maternal-health focus early enough. Please open with a more focused topic sentence, and delete lines 75–83. A related issue runs through the manuscript: war and disaster are not interchangeable terms they have distinct definitions and produce different health system impacts. As the study is set in a conflict context rather than a natural-disaster context, please use war / armed conflict terminology consistently throughout.

Reply to comment: We thank the reviewers. These lines were deleted as requested. Anew paragraph was added to the Introduction section as requested:

Armed conflicts place exceptional strain on healthcare infrastructures, compelling both institutions and practitioners to swiftly adapt in order to continue providing effective care amid uncertainty and limited resources. Common responses to these pressures include reallocating medical staff, modifying patterns of patient referral and hospitalization to accommodate surges of injured military personnel and civilians, and reorganizing hospital operations to balance urgent demands with routine healthcare services [1–11]. One frequent strategy employed during wartime is the reduction of hospital length of stay for select groups, such as low-risk postpartum women. This approach is intended to maximize bed availability and minimize patients’ exposure to conflict-related risks. However, the potential impacts of shortened hospitalization on patient outcomes within this context remain insufficiently understood.

2. Lines 110–112 relocate or delete. These lines sit awkwardly in the introduction. Their generalising claim that the findings may be relevant to conflicts and disasters more broadly belongs in the discussion or conclusion, after the specific findings have been presented. Please consider deleting these lines from the introduction or relocating them.

Reply to comment: These lines were deleted as requested.

3. Introduce Israel’s health-system context early, not in the limitations. A point currently raised only in the limitations should be introduced in the introduction or early methods: Israel is a high-income setting with mature emergency and trauma care infrastructure, shaped both by repeated exposure to armed conflict and by substantial domestic investment in trauma medicine. Establishing this context early frames the generalisability of the findings appropriately for an international readership.

Reply to comment: The data was added to the Introduction as requested.

4. Lines 94–98, 119–120, and 298–299 adopt objective scientific language and add specific contextual anchors. These lines describe the events of 7 October and the resulting loss of life. While the gravity of those events is fully acknowledged, a scientific manuscript for an international audience should present this background in objective language, without evaluative adjectives and without details that do not bear directly on the study aim or methods. Elements such as the regional evacuation are worth retaining because they describe a population-level shift relevant to the analysis. The authors should also state explicitly that, following the attack on 7 October, Israel formally declared a state of war on 8 October, and that the present study covers only the first month of that period.

Reply to comment: Changes were made as requested:

On October 7, 2023, Hamas organization launched a war against Israel including massive missile attacks and territory invasion, resulting in over 1,400 lives lost and the abduction of 251 hostages. On October 8th 2023 Hezbollah subsequently joined, launching rockets at Israel, including attacks targeting civilian populations, leading to declaration of war by Israel.

5. Clarify the scope of the early-discharge protocol. Please clarify whether the early-discharge protocol described in the methods is specific to the study centre, or whether it reflects a national directive issued in response to the war declaration. This affects how readers will interpret generalisability.

Reply to comment: During that period the Israeli Ministry of Health revised its guidelines and permitted hospitals to significantly shorten the length of postpartum hospitalization. This data was added as requested to the Method section.

6. Outcome ascertainment and post-discharge follow-up. Reviewer 1 raised this in the previous round and the response was not, in our view, sufficient. Please clarify (a) whether discharged women received structured follow-up for example, telephone contact or written instructions identifying warning signs and points of presentation — and (b) how the authors are confident that women experiencing complications were captured by the study, given they could plausibly have presented to emergency departments outside the study site (particularly relevant in the context of regional evacuations). If outside-site presentations cannot be ascertained from the available data, this must be stated explicitly as a limitation affecting outcome ascertainment.

Reply to comment: a) Each postpartum woman received detailed explanations from both an obstetrician and a maternity nurse, as well as a discharge letter specifying when to seek medical attention (for example, in cases of fever, bleeding, abnormal discharge, unusual appearance of the surgical scar, etc).

b) We do not have data regarding whether women who were discharged sought care at other medical centers following delivery. However, not all clinics were operational during the war. Given that our hospital is a tertiary care center located in central Israel—and not in the north or south, where missile attacks were significantly more frequent—we assume that if a postpartum woman required medical attention, she would have presented to our facility. This data was added to the Limitation section in the Discussion section.

Methods and Results

7. Table 2 interpretation of time from delivery to GER referral. The reported time from delivery to GER referral appears shorter in the wartime cohort (median 5.9 [IQR 4.5–10.6]) than in the control (median 7.5 [IQR 4.5–16.0]), with a Wilcoxon p-value of 0.16, yet the text states there was no significant difference in the time elapsed from delivery to postpartum referral. The point estimate is approximately 1.6 days earlier presentation in the wartime cohort, with a meaningfully wider tail in the control cohort. This is precisely the pattern that would be expected if early-discharged women re-present sooner because they are sent home before the natural window for early postpartum complications has closed. Characterising this as "no significant difference" obscures a clinically interpretable signal. Please:

(a) confirm whether the values reported are means or medians, with corresponding SD or IQR, and report the exact test used and p-value;

(b) discuss whether the apparent ~1.6-day earlier presentation, even if not statistically significant, is clinically meaningful that is, whether it represents a signal that women discharged earlier under the wartime protocol required postpartum care sooner than the control cohort.

A non-significant p-value with a clinically suggestive point estimate warrants discussion rather than dismissal.

Reply to comment: We thank you for your comment.

a) The values reported are indeed median.

b) We added the information regarding the 1.6 days difference between early discharge to the control group to the Result section and the Discussion section.

8. Confounders and mediators terminology. The terms confounder and mediator are used inconsistently across the manuscript. These are distinct epidemiological constructs and should not be used interchangeably. The methods correctly identify delivery characteristics and length-of-stay as mediators on the causal pathway, but elsewhere in the text the language slips. Please audit the manuscript for consistency and tighten it throughout.

Reply to comment: thank you for the useful comment. We double checked the phrasing and made sure that delivery characteristics are referred to as mediators,

and mother's characteristics (age at delivery, gestational week, fetus count, and number of previous births) are referred to as confounders and therefore are adjusted for in the multivariable model.

9. Discuss the shift in the elective caesarean fraction (Table 1). Table 1 reports that 57% of caesarean sections in the wartime cohort were classified as elective, compared with 92% in the control period (p < 0.0001). This 35-percentage-point shift is the single largest difference between the two cohorts and is currently mentioned only in passing. Three interpretations are possible and have substantially different implications for how the null finding on GER referral should be read:

(a) Elective caesareans were rationally deferred during wartime for resource or scheduling reasons, a parallel intervention, separate from early discharge.

(b) The wartime cohort experienced a genuine increase in obstetric complications, driving non-elective and urgent caesareans an effect of war that should be reflected in other outcomes;

(c) The population delivering during wartime differed from the control population due to regional evacuations or altered referral patterns, a case-mix shift that the current confounder set does not capture.

The authors are asked to discuss this finding explicitly in the manuscript

Reply to comment: The following paragraph was added to the Discussion section:

An interesting finding in the present study is the significant reduction in elective cesarean sections during wartime (57% vs. 92% of total cesareans, p < 0.0001). This sharp decline may reflect significant shifts in obstetric decision-making, perhaps influenced by patients' concerns over extended hospitalization and potential disruptions in postnatal care. Another possible explanation is a problem of resources or scheduling reasons. However, our hospital is a tertiary medical center, and the changes in personnel did not result in cancellations of elective cesarean sections. Another possibility may be that the population delivering during wartime differed from the control population due to regional evacuations or altered referral patterns. A nationwide study is required. An alternative explanation may be that the war caused an increase in obstetrical complications increasing the rates of non-elective and urgent cesarean sections. Despite the decline in elective cesarean sections, there were no significant differences in neonatal outcomes, including rates of meconium-stained amniotic fluid, low Apgar scores or rate of admissions to the Newborn Intensive Care Unit indicating that this strategic change did not adversely affect neonates.

10. Cohort derivation is not described and should be reported in STROBE-compliant form. The methods state that all deliveries within each study window were included, but the manuscript provides no inclusion/exclusion algorithm, no flow diagram, and no information on exclusions for fetal demise, antenatal transfer, incomplete electronic medical record data, or other reasons. STROBE adherence is not stated. For an observational cohort study using administrative data, this is a substantial reporting gap. Please provide a STROBE flow diagram and a clear inclusion/exclusion statement, and add a STROBE adherence statement to the methods.

Reply to comment:

The present study included all deliveries during the study period. No exclusions were made. Since all data regarding deliveries in our medical center is stored in a computerized database, we have complete and comprehensive information about each delivery, with no missing data. This data was added to the Method section.

The study was conducted in accordance with the Strengthening the Reporting of Observational Studies in Epidemiology (STROBE) guidelines. This information was added to the Method section.

11. Specify the operational definition of "low-risk" and consider an as-treated analysis. The definition of patients eligible for early discharge "generally healthy women, with no delivery complications such as profound vaginal bleeding, hypertension or infection" is clinical rather than operational. The manuscript does not specify (a) the criteria that determined eligibility for early discharge in practice, (b) whether pre-existing maternal conditions (diabetes, mental health history, BMI) affected eligibility, or (c) what proportion of the wartime cohort actually received the abbreviated stay. This matters because the analysis treats the entire wartime cohort as exposed to the early-discharge intervention, when in reality only a subset would have been eligible. Please specify the operational criteria, report the proportion of each cohort that received early discharge, and present an as-treated sensitivity analysis restricted to women who actually received the abbreviated stay.

Reply to comment:

We thank you for these thoughtful and detailed comments.

We would like to clarify that the primary aim of our study was to evaluate the overall impact of reduced postpartum hospitalization duration on GER visits, rather than focusing on specific groups, providing a comprehensive overview of the clinical outcomes following policy implementation. Our intension was to evaluate whether the system-wide change in discharge policy was associated with a significant increase in postpartum GER visits in the general population. As our results demonstrate, we did not observe an increase in the frequency of such visits across the entire cohort, suggesting that the policy did not adversely affect this outcome in the population scale. We recognize the value of a more granular analysis. Indeed, future studies are warranted to explore the effects of shortened postpartum hospitalization on emergency department visits among various sub populations, such as women with elevated BMI, diabetes, mental health disorders and other relevant comorbidities. Such research could provide important insight into the different impact of early discharge policies on maternal health outcomes. We recommended in the Discussion section to perform in a future study a comparison between low and high-risk pregnancies.

Here are three articles addressing the shortening of hospitalization among postpartum women during the COVID-19 pandemic. Similar to our study, these articles also examined the entire population within their respective hospitals:

• Early postpartum discharge during the COVID-19 pandemic.

Bornstein E, Gulersen M, Husk G, Grunebaum A, Blitz MJ, Rafael TJ, Rochelson BL, Schwartz B, Nimaroff M, Chervenak FA.J Perinat Med. 2020 Nov 26;48(9):1008-1012. doi: 10.1515/jpm-2020-0337.PMID: 32845868.

• Length of Postpartum Hospital Stays During COVID-19: Findings From a Convergent Parallel Mixed-Methods Study.

Correia RH, Greyson D, Carruthers A, Kuyvenhoven C, Kirkwood D, Jones A, Howard M, Darling E, Davis A, McDonald SD, Mniszak C, Kandasamy S, Vanstone M.J Obstet Gynaecol Can. 2024 Oct;46(10):102637. doi: 10.1016/j.jogc.2024.102637. Epub 2024 Aug 21.PMID: 39154662.

• The Risk of Readmission after Early Postpartum Discharge during the COVID-19 Pandemic.

Gulersen M, Husk G, Lenchner E, Blitz MJ, Rafael TJ, Rochelson B, Chakravarthy S, Grunebaum A, Chervenak FA, Fruhman G, Jones MF, Schwartz B, Nimaroff M, Bornstein E.Am J Perinatol. 2022 Mar;39(4):354-360. doi: 10.1055/s-0041-1740061. Epub 2021 Dec 10.PMID: 34891201.

12. Reasons-for-GER analysis classifies only a minority of referrals. The analysis of indications for postpartum GER referral classifies only infection (11 + 13 = 24 cases) and bleeding (0 + 4 = 4 cases), totalling 28 of the 150 GER referrals approximately 19% of all cases. The remaining ~120 referrals are not categorised. The statement that "there were also no significant differences in the reasons for the postpartum referrals" cannot be supported when the majority of referrals have not been classified. Please either (a) provide a complete categorisation of all GER referrals by indication, with appropriate adjustment for multiple comparisons, or (b) remove this analysis from the manuscript.

Reply to comment: Table 2 was erased. A paragraph about the postpartum visits and hospitalization was added to the Result section.

13. The confounder set is thin for a wartime/peacetime comparison. The multivariable models adjust for age at delivery, gestational week (<37 vs ≥37), fetus count, and parity. The exclusion of delivery characteristics and length of stay on the grounds that they are mediators is appropriate. However, several variables that are plausibly associated with both calendar period and the outcome, and which are not on the mediator pathway, have been omitted without justification:

(a) Maternal comorbidities eg. pre-existing hypertension, diabetes, obesity, thyroid disease, and mental health history. These conditions pre-date the exposure window and are established predictors of postpartum complications and ED re-presentation.

(b) Geographic and socioeconomic variables area of residence, distance to the index hospital, and any indicator of displacement status. Specifically in the wartime cohort, internal displacement following regional evacuations is a likely source of confounding that the current model cannot address.

Please explicitly acknowledge in the limitations the residual confounding that remains if these variables are not available in the dataset.

Reply to comment: We thank you for these insightful comments. As clarified, the primary objective of our study was to evaluate the overall impact of the early discharge policy on postpartum visits to the GER, taking a population-level approach akin to prior studies conducted during the COVID-19 pandemic. While we agree that maternal comorbidities and geographic or socioeconomic factors are important potential confounders, the capacity to adjust for these variables was limited in the context of this unique wartime event.

Specifically, large-scale evacuations from both northern and southern regions of Israel during the war rendered residential address and related geographic or socioeconomic indicators largely unreliable for analysis, as most evacuees lost their homes and ceased employment, greatly challenging the accurate assessment of these variables.

As we wrote in comment no. 11, future studies are warranted to explore the effects of shortened postpartum hospitalization on emergency department visits among various sub populations, such as women with elevated BMI, diabetes, mental health disorders and other relevant comorbidities. Such research could provide important insight into the different impact of early discharge policies on maternal health outcomes. We recommended in the Discussion section to perform in a future study a comparison between low and high-risk pregnancies.

14. "No difference" framing overstates the precision of the estimates. The manuscript repeatedly characterises the findings as showing "no difference" between the wartime and control periods. This phrasing edges toward an equivalence claim that the data do not support. The 95% confidence interval for the multivariable odds ratio (0.72–1.41) is compatible with up to a 28% reduction or a 41% increase in the odds of postpartum GER referral. The study has ruled out only large effects; clinically and policy-relevant moderate effects in either direction remain plausible. The post hoc power statement implicitly acknowledges this, yet the language of the discussion is not consistent with it. A more accurate framing throughout, particularly in the abstract, results, and conclusion, would be: "We found no evidence of a difference between the wartime and control periods, with confidence intervals compatible with moderate increases or decreases in postpartum GER risk." Equivalence cannot be claimed without a pre-specified equivalence margin and the corresponding test, which were not performed.

Reply to comment: We agree with the comment and added the sentence suggested.

15. Smaller methodological and reporting points. A number of additional points should be addressed:

(a) The methods (line 142) define the postpartum period as 42 days, while Figure 1 plots 45 days. Please reconcile.

(b) Missing data handling is not described. Please specify whether complete-case analysis was used and report the extent of missingness for each variable in the analytical model.

(c) p-values reported as exactly "1" in Table 2 should be reported as ">0.99" to avoid the misleading impression of perfect agreement.

(d) The single-centre, tertiary-hospital design limits generalisability to other Israeli hospitals — particularly those serving evacuated regions — and to other conflict settings. This should be acknowledged more prominently in the limitations.

Reply to comment:

a) Legend of Figure 1 was changed, thank you.

b) There was no missing data.

c) Table 2 was erased as suggested in comment no. 12.

d) We thank the editor for their comment. As Chaim Sheba medical Center is a large, tertiary medical center, caring for highly diverse maternal population, including many who were evacuated during the war, we believe that this increases the potential generalizability of our findings, and is one of the study's main strength.

Reviewers' comments:

Reviewer's Responses to Questions

Comments to the Author

1. If the authors have adequately addressed your comments raised in a previous round of review and you feel that this manuscript is now acceptable for publication, you may indicate that here to bypass the “Comments to the Author” section, enter your conflict of interest statement in the “Confidential to Editor” section, and submit your "Accept" recommendation.

Reviewer #7: All comments have been addressed

________________________________________

2. Is the manuscript technically sound, and do the data support the conclusions?

The manuscript must describe a technically sound piece of scientific research with data that supports the conclusions. Experiments must have been conducted rigorously, with appropriate controls, replication, and sample sizes. The conclusions must be drawn appropriately based on the data presented.

Reviewer #7: Yes

________________________________________

3. Has the statistical analysis been performed appropriately and rigorously?

Reviewer #7: Yes

________________________________________

4. Have the authors made all data underlying the findings in their manuscript fully available?

The PLOS Data policy requires authors to make all data underlying the findings described in their manuscript fully available without restriction, with rare exception (please refer to the Data Availability Statement in the manuscript PDF file). The data should be provided as part of the manuscript or its supporting information, or deposited to a public repository. For example, in addition to summary statistics, the data points behind means, medians and variance measures should be available. If there are restrictions on publicly sharing data—e.g. participant privacy or use of data from a third party—those must be specified.

Reviewer #7: Yes

________________________________________

5. Is the manuscript presented in an intelligible fashion and written in standard English?

PLOS ONE does not copyedit accepted manuscripts, so the language in submitted articles must be clear, correct, and unambiguous. Any typographical or grammatical errors should be corrected at revision, so please note any specific errors here.

Reviewer #7: Yes

________________________________________

6. Review Comments to the Author

Please use the space provided to explain your answers to the questions above. You may also include additional comments for the author, including concerns about dual publication, research ethics, or publication ethics. (Please upload your review as an attachment if it exceeds 20,000 characters)

Reviewer #7: The authors have comprehensively addressed all the comments and concerns raised in my previous review of the manuscript

________________________________________

7. PLOS authors have the option to publish the peer review history of their article (what does this mean?). If published, this will include your full peer review and any attached files.

If you choose “no”, your identity will remain anonymous but your review may still be made public.

Do you want your identity to be public for this peer review? For information about this choice, including consent withdrawal, please see our Privacy Policy.

Reviewer #7: No

Attachment Submitted filename: 6th responce to reviewers.docx

# Editor report: Decision Letter 6

**DOI:** 10.1371/journal.pone.0335688.r013

**Authors:** Danish Ahmad

7 Jul 2026

Early Hospital Discharge Following Delivery During Wartime is not Associated with Increased Rates of Postpartum Hospital Referrals

PLOS One

Dear Dr. Weissmann-Brenner,

Thank you for submitting your manuscript to PLOS ONE. After careful consideration, we feel that it has merit but does not fully meet PLOS ONE’s publication criteria as it currently stands. Therefore, we invite you to submit a revised version of the manuscript that addresses the points raised during the review process.

Please submit your revised manuscript by Aug 21 2026 11:59PM. If you will need more time than this to complete your revisions, please reply to this message or contact the journal office at plosone@plos.org. When you're ready to submit your revision, log on to https://www.editorialmanager.com/pone/ and select the 'Submissions Needing Revision' folder to locate your manuscript file.

Please include the following items when submitting your revised manuscript:

- A letter that responds to each point raised by the academic editor and reviewer(s). You should upload this letter as a separate file labeled 'Response to Reviewers'.
- A marked-up copy of your manuscript that highlights changes made to the original version. You should upload this as a separate file labeled 'Revised Manuscript with Track Changes'.
- An unmarked version of your revised paper without tracked changes. You should upload this as a separate file labeled 'Manuscript'.

If you would like to make changes to your financial disclosure, please include your updated statement in your cover letter. Guidelines for resubmitting your figure files are available below the reviewer comments at the end of this letter.

If applicable, we recommend that you deposit your laboratory protocols in protocols.io to enhance the reproducibility of your results. Protocols.io assigns your protocol its own identifier (DOI) so that it can be cited independently in the future. For instructions see: https://journals.plos.org/plosone/s/submission-guidelines#loc-laboratory-protocols. Additionally, PLOS ONE offers an option for publishing peer-reviewed Lab Protocol articles, which describe protocols hosted on protocols.io. Read more information on sharing protocols at https://plos.org/protocols?utm_medium=editorial-email&utm_source=authorletters&utm_campaign=protocols.

As the corresponding author, your ORCID iD is verified in the submission system and will appear in the published article. PLOS supports the use of ORCID, and we encourage all coauthors to register for an ORCID iD and use it as well. Please encourage your coauthors to verify their ORCID iD within the submission system before final acceptance, as unverified ORCID iDs will not appear in the published article. *Only*  the individual author can complete the verification step; PLOS staff *cannot*  verify ORCID iDs on behalf of authors.

We look forward to receiving your revised manuscript.

Kind regards,

Alejandro Torrado Pacheco, PhD

Associate Editor

PLOS One

with

Danish Ahmad, MBBS,MSc,MNAMS,PhD,IP-FPH(UK),FRCP(Edin),FRCP(Lon)

Academic Editor

PLOS One

Journal Requirements:

If the reviewer comments include a recommendation to cite specific previously published works, please review and evaluate these publications to determine whether they are relevant and should be cited. There is no requirement to cite these works unless the editor has indicated otherwise.

Please review your reference list to ensure that it is complete and correct. If you have cited papers that have been retracted, please include the rationale for doing so in the manuscript text, or remove these references and replace them with relevant current references. Any changes to the reference list should be mentioned in the rebuttal letter that accompanies your revised manuscript. If you need to cite a retracted article, indicate the article’s retracted status in the References list and also include a citation and full reference for the retraction notice.

Additional Editor Comments:

Thank you for the revised manuscript and the detailed response to revision comments. The authors are commended for undertaking multiple revisions to incorporate reviewers' comments as evidenced through the multiple reviewer rounds.

Before we proceed to acceptance, please address the following final minor comments:

Please ensure your Methods section includes the date when data were accessed for research purposes, in DD/MM/YYYY format.

Please also address the following typographical/grammatical errors, and review the text for any other errors:

- Line 27: replace "=" with "-"

- Line 32: replace "and 913 delivered..." with "and 913 who delivered"

- Line 49: remove duplicate ethics statement

- Line 94: replace "deranged" with more accurate wording (e.g. "impacted")

- Line 122: replace "health" with "healthy"

- Line 316: remove stray period

- Line 321: replace "import" with "important"

[Note: HTML markup is below. Please do not edit.]

[NOTE: If reviewer comments were submitted as an attachment file, they will be attached to this email and accessible via the submission site. Please log into your account, locate the manuscript record, and check for the action link "View Attachments". If this link does not appear, there are no attachment files.]

To ensure your figures meet our technical requirements, please review our figure guidelines: https://journals.plos.org/plosone/s/figures

You may also use PLOS’s free figure tool, NAAS, to help you prepare publication quality figures: https://journals.plos.org/plosone/s/figures#loc-tools-for-figure-preparation.

NAAS will assess whether your figures meet our technical requirements by comparing each figure against our figure specifications.

# Author comment: Author response to Decision Letter 7

**DOI:** 10.1371/journal.pone.0335688.r014

7 Jul 2026

Date: Tuesday, July 7, 2026

To:

Alejandro Torrado Pacheco, PhD

Danish Ahmad, MBBS,MSc,MNAMS,PhD,IP-H(UK),FRCP(Edin),FRCP(Lon)

Academic Editor

PLOS ONE

Re: PONE-D-24-57057 entitled:

Early Hospital Discharge Following Delivery During Wartime is not Associated with Increased Rates of Postpartum Hospital Referrals,

Dear Dr. Alejandro Torrado Pacheco, and Dr. Danish Ahmad,

We would like to thank you, the editors of PLOS ONE Journal for considering our manuscript for PLOS ONE Journal and for your time and efforts to review this manuscript.

We have addressed each comment in the following reply and have revised the manuscript accordingly. We hope that the 7h revised version of the manuscript is considered suitable for publication in PLOS ONE Journal.

An itemized reply to the comments of the Reviewers is provided below.

Yours sincerely,

Prof. Alina Weissmann-Brenner.

Reviewers' comments:

Please also address the following typographical/grammatical errors, and review the text for any other errors:

- Line 27: replace "=" with "-"

Response to comment: Thank you, replacement performed as requested.

- Line 32: replace "and 913 delivered..." with "and 913 who delivered"

Response to comment: Thank you, replacement performed as requested.

- Line 49: remove duplicate ethics statement

Response to comment: Thank you, removal performed as requested.

- Line 94: replace "deranged" with more accurate wording (e.g. "impacted")

Response to comment: Thank you, replacement performed as requested.

- Line 122: replace "health" with "healthy"

Response to comment: Thank you, replacement performed as requested.

- Line 316: remove stray period

Response to comment: Thank you, removal performed as requested.

- Line 321: replace "import" with "important"

Response to comment: Thank you, replacement performed as requested.

Attachment Submitted filename: 7th responce to reviewers.docx

# Editor report: Decision Letter 7

**DOI:** 10.1371/journal.pone.0335688.r015

**Authors:** Danish Ahmad

28 Jul 2026

Early Hospital Discharge Following Delivery During Wartime is not Associated with Increased Rates of Postpartum Hospital Referrals

PLOS One

Dear Dr.  Weissmann-Brenner,

Thank you for submitting your manuscript to PLOS ONE. After careful consideration, we feel that it has merit but does not fully meet PLOS ONE’s publication criteria as it currently stands. Therefore, we invite you to submit a revised version of the manuscript that addresses the points raised during the review process.

- The journal has identified that the last revision included one new co-author (Nirit Aagay) who was added. However, in the revised paper, there is no author contribution linked to added co-author. Please add a brief author contribution relevant to them. Information on authorship guidelines for Plos are available at:Authorship | PLOS One

plosone@plos.org. When you're ready to submit your revision, log on to https://www.editorialmanager.com/pone/ and select the 'Submissions Needing Revision' folder to locate your manuscript file.

- A letter that responds to each point raised by the academic editor and reviewer(s). You should upload this letter as a separate file labeled 'Response to Reviewers'.
- A marked-up copy of your manuscript that highlights changes made to the original version. You should upload this as a separate file labeled 'Revised Manuscript with Track Changes'.
- An unmarked version of your revised paper without tracked changes. You should upload this as a separate file labeled 'Manuscript'.

If you would like to make changes to your financial disclosure, please include your updated statement in your cover letter. Guidelines for resubmitting your figure files are available below the reviewer comments at the end of this letter.

If applicable, we recommend that you deposit your laboratory protocols in protocols.io to enhance the reproducibility of your results. Protocols.io assigns your protocol its own identifier (DOI) so that it can be cited independently in the future. For instructions see: https://journals.plos.org/plosone/s/submission-guidelines#loc-laboratory-protocols. Additionally, PLOS ONE offers an option for publishing peer-reviewed Lab Protocol articles, which describe protocols hosted on protocols.io. Read more information on sharing protocols at https://plos.org/protocols?utm_medium=editorial-email&utm_source=authorletters&utm_campaign=protocols.

As the corresponding author, your ORCID iD is verified in the submission system and will appear in the published article. PLOS supports the use of ORCID, and we encourage all coauthors to register for an ORCID iD and use it as well. Please encourage your coauthors to verify their ORCID iD within the submission system before final acceptance, as unverified ORCID iDs will not appear in the published article. *Only*  the individual author can complete the verification step; PLOS staff *cannot*  verify ORCID iDs on behalf of authors.

We look forward to receiving your revised manuscript.

Kind regards,

Alejandro Torrado Pacheco, PhD

Associate Editor

PLOS One

with

Danish Ahmad, MBBS,MSc,MNAMS,PhD,IP-FPH(UK),FRCP(Edin),FRCP(Lon)

Academic Editor

PLOS One

Journal Requirements:

If the reviewer comments include a recommendation to cite specific previously published works, please review and evaluate these publications to determine whether they are relevant and should be cited. There is no requirement to cite these works unless the editor has indicated otherwise.

Please review your reference list to ensure that it is complete and correct. If you have cited papers that have been retracted, please include the rationale for doing so in the manuscript text, or remove these references and replace them with relevant current references. Any changes to the reference list should be mentioned in the rebuttal letter that accompanies your revised manuscript. If you need to cite a retracted article, indicate the article’s retracted status in the References list and also include a citation and full reference for the retraction notice.

Additional Editor Comments:

Dear Authors

Thank you for submitting your revised manuscript. The previous decision letter included a request to add the date when data were accessed for research purposes in the Methods section. We note this date was included in earlier versions of the manuscript but has since been removed. Could you please update your Methods section accordingly?

Thank you for the revised manuscript. The journal has identified the addition of a new co-author(see below) at the last revision. The authorship contribution section does not acknowledge the added co-authors specific contribution. Either add those details or please revise the authorship to only those whose contribution fullfills ICJME coauthorship criterion.

Nirit Aagay- Chaim Sheba Medical Center: Sheba Medical Center at Tel Hashomer - nirita@gertner.health.gov.il

[Note: HTML markup is below. Please do not edit.]

[NOTE: If reviewer comments were submitted as an attachment file, they will be attached to this email and accessible via the submission site. Please log into your account, locate the manuscript record, and check for the action link "View Attachments". If this link does not appear, there are no attachment files.]

To ensure your figures meet our technical requirements, please review our figure guidelines: https://journals.plos.org/plosone/s/figures

You may also use PLOS’s free figure tool, NAAS, to help you prepare publication quality figures: https://journals.plos.org/plosone/s/figures#loc-tools-for-figure-preparation.

NAAS will assess whether your figures meet our technical requirements by comparing each figure against our figure specifications.

# Author comment: Author response to Decision Letter 8

**DOI:** 10.1371/journal.pone.0335688.r016

12 Aug 2026

Date: Tuesday, August 11, 2026

To:

Alejandro Torrado Pacheco, PhD

Danish Ahmad, MBBS,MSc,MNAMS,PhD,IP-H(UK),FRCP(Edin),FRCP(Lon)

Academic Editor

PLOS ONE

Re: PONE-D-24-57057 entitled:

Early Hospital Discharge Following Delivery During Wartime is not Associated with Increased Rates of Postpartum Hospital Referrals,

Dear Dr. Alejandro Torrado Pacheco, and Dr. Danish Ahmad,

We would like to thank you, the editors of PLOS ONE Journal, and the reviewers for considering our manuscript for PLOS ONE Journal and for your time and efforts to review this manuscript.

We added the contribution of Mrs. Agay Nirit to the manuscript. We hope that the 8th revised version of the manuscript is considered suitable for publication in PLOS ONE Journal.

An Exel file with the data is attached.

Why this manuscript is suitable for publication in PLOS ONE:

Our study addresses a timely and relevant issue in maternal health by examining the effects of early postpartum discharge protocols during wartime conditions. PLOS ONE’s interdisciplinary and inclusive scope welcomes research that provides rigorous evidence across diverse settings, and our findings contribute valuable insights into postpartum care management under conflict-related healthcare constraints. The study's methodology and data are robust, and the results have broad implications for healthcare providers and policymakers operating in challenging environments.

How our paper provides a worthwhile addition to the scientific literature:

While previous research has explored postpartum discharge practices under normal circumstances, there is a paucity of data examining their impact during wartime or conflict scenarios. Our study fills this gap by demonstrating that early discharge during such times does not negatively affect subsequent referrals and admissions, thus supporting flexible postpartum care strategies in crisis settings. This contributes to the growing body of literature on adaptive healthcare practices during emergencies, informing evidence-based decision-making to optimize maternal outcomes.

How our paper relates to previously published work:

To our knowledge, this is the first study to examine the feasibility of early postpartum discharge of uncomplicated deliveries in the setting of wartime, providing valuable insights from a large, tertiary medical center with diverse maternal demographics, enhancing the generalizability of our findings.

Which types of scientists will find our study most interesting:

We believe that obstetricians, midwives, global health researchers, healthcare policy makers, and humanitarian aid organizations will find our study particularly relevant. Scholars interested in maternal health in resource-constrained or emergency settings, as well as those studying healthcare delivery during conflicts, will benefit from our findings and insights.

Yours sincerely,

Prof. Alina Weissmann-Brenner.

Attachment Submitted filename: 8th responce to reviewers.docx

# Editor report: Decision Letter 8

**DOI:** 10.1371/journal.pone.0335688.r017

**Authors:** Danish Ahmad

18 Aug 2026

Early Hospital Discharge Following Delivery During Wartime is not Associated with Increased Rates of Postpartum Hospital Referrals

PONE-D-24-57057R8

Dear Dr.Alina Weissmann-Brenner

We’re pleased to inform you that your manuscript has been judged scientifically suitable for publication and will be formally accepted for publication once it meets all outstanding technical requirements.

Within one week, you’ll receive an e-mail detailing the required amendments. When these have been addressed, you’ll receive a formal acceptance letter and your manuscript will be scheduled for publication.

**Comment from staff editor**

Please include the following sentence, as stated in an earlier version of the paper, in the Methods section of your final manuscript files: "Data was retrieved on 22/02/24 only after an approval was given by our institutional review board.". Thank you.

An invoice will be generated when your article is formally accepted. Please note, if your institution has a publishing partnership with PLOS and your article meets the relevant criteria, all or part of your publication costs will be covered. Please make sure your user information is up-to-date by logging into Editorial Manager at Editorial Manager® and clicking the ‘Update My Information' link at the top of the page. For questions related to billing, please contact billing support.

If your institution or institutions have a press office, please notify them about your upcoming paper to help maximize its impact. If they’ll be preparing press materials, please inform our press team as soon as possible -- no later than 48 hours after receiving the formal acceptance. Your manuscript will remain under strict press embargo until 2 pm Eastern Time on the date of publication. For more information, please contact onepress@plos.org.

Kind regards,

Alejandro Torrado Pacheco, PhD

Associate Editor

PLOS One

with,

Danish Ahmad, MBBS,MSc,MNAMS,PhD,IP-FPH(UK),FRCP(Edin),FRCP(Lon)

Academic Editor

PLOS One

Additional Editor Comments (optional):

Thank you, Dr Alina, for working with the journal to address the multiple reviewers rounds and for bringing this work to fruition.

# Editor report: Acceptance letter

**DOI:** 10.1371/journal.pone.0335688.r018

**Authors:** Danish Ahmad

PONE-D-24-57057R8

PLOS One

Dear Dr. Weissmann-Brenner,

I'm pleased to inform you that your manuscript has been deemed suitable for publication in PLOS One. Congratulations! Your manuscript is now being handed over to our production team.

At this stage, our production department will prepare your paper for publication. This includes ensuring the following:

* All references, tables, and figures are properly cited

* All relevant supporting information is included in the manuscript submission,

* There are no issues that prevent the paper from being properly typeset

You will receive further instructions from the production team, including instructions on how to review your proof when it is ready. Please keep in mind that we are working through a large volume of accepted articles, so please give us a few days to review your paper and let you know the next and final steps.

Lastly, if your institution or institutions have a press office, please let them know about your upcoming paper now to help maximize its impact. If they'll be preparing press materials, please inform our press team within the next 48 hours. Your manuscript will remain under strict press embargo until 2 pm Eastern Time on the date of publication. For more information, please contact onepress@plos.org.

You will receive an invoice from PLOS for your publication fee after your manuscript has reached the completed accept phase. If you receive an email requesting payment before acceptance or for any other service, this may be a phishing scheme. Learn how to identify phishing emails and protect your accounts at https://explore.plos.org/phishing.

If we can help with anything else, please email us at customercare@plos.org.

Thank you for submitting your work to PLOS One and supporting open access.

Kind regards,

PLOS One Editorial Office Staff

on behalf of

Dr. Danish Ahmad

Academic Editor

PLOS One
